# A multi-scale unified model of human mobility in urban agglomerations

**DOI:** 10.1016/j.patter.2023.100862

**Published:** 2023-10-17

**Authors:** Yong Chen, Haoge Xu, Xiqun (Michael) Chen, Ziyou Gao

**Affiliations:** 1 Institute of Intelligent Transportation Systems, College of Civil Engineering and Architecture, Zhejiang University, Hangzhou 310058, China; 2 Zhejiang University/University of Illinois Urbana-Champaign (ZJU-UIUC) Institute, Haining 314400, China; 3 School of Systems Science, Beijing Jiaotong University, Beijing 100044, China

**Keywords:** human mobility, urban agglomeration, human behavior, generative adversarial network, deep learning, convolutional neural network, hierarchical travel choice, multi-scale travel

## Abstract

Understanding human mobility patterns is vital for the coordinated development of cities in urban agglomerations. Existing mobility models can capture single-scale travel behavior within or between cities, but the unified modeling of multi-scale human mobility in urban agglomerations is still analytically and computationally intractable. In this study, by simulating people’s mental representations of physical space, we decompose and model the human travel choice process as a cascaded multi-class classification problem. Our multi-scale unified model, built upon cascaded deep neural networks, can predict human mobility in world-class urban agglomerations with thousands of regions. By incorporating individual memory features and population attractiveness features extracted by a graph generative adversarial network, our model can simultaneously predict multi-scale individual and population mobility patterns within urban agglomerations. Our model serves as an exemplar framework for reproducing universal-scale laws of human mobility across various spatial scales, providing vital decision support for urban settings of urban agglomerations.

## Highlights

• We propose a multi-scale unified model of human mobility in urban agglomerations
• A cascaded deep neural network is used to simulate human travel choice processes
• A generative adversarial network is used to extract population attractiveness features
• The model can predict multi-scale travel patterns for individuals and populations

## The bigger picture

As urban areas develop, neighboring cities gradually converge to form highly integrated urban spatial forms through a process known as urban agglomeration. Within urban agglomerations, travel occurs at different spatial scales, for example, within local neighborhoods, within cities, or between cities. This makes understanding human mobility in urban agglomerations inherently complex. While various models have been developed in the past to describe human mobility, they generally cannot model and predict the complex multi-scale travel that occurs within urban agglomerations. Methods that can better model mobility in complex urban agglomerations could have significant practical implications for topics such as urban resource management, disease control, and transportation hub optimization.

## Teaser

One major weakness of current human mobility models is their inability to accurately measure and quantify large-scale human movements between regions in urban agglomerations. By simulating people’s mental representations of physical space, a novel multi-scale unified model based on cascaded deep neural networks is proposed for human mobility prediction within urban agglomerations. The model simulates people’s hierarchical travel selection behavior and can reproduce the universal-scale laws of individuals and populations in urban agglomerations with thousands of regions.

## Introduction

With the urban economy’s rapid development, megacities, metropolitan areas, and their neighboring cities are gradually converging to form highly developed integrated urban spatial forms,^1^ known as the urban agglomeration.^2^ Each integrated city is contained in a compact space and maintains close economic ties within its urban agglomeration. Furthermore, as one of the forces most behind global economic development,^3^ urban agglomerations have affected countries’ strategic layouts and global competitiveness.^1^^,^^4^ Accordingly, they have drawn increasing attention from governmental authorities worldwide, and various outlines and strategies^5^^,^^6^^,^^7^ have been formulated to promote industrial cooperation, resource management and optimization, and complementary infrastructure between cities to comprehensively integrate urban agglomerations.

Within urban agglomerations, people are driven by various travel needs to conduct activities at different spatial scales. Such inter- or intra-city movements and interactions promote economic circulation and cultural exchange between cities in urban agglomerations.^8^ Consequently, understanding human mobility in urban agglomerations is important and has significant practical implications, such as controlling the spread of disease,^9^^,^^10^ optimizing transportation hubs,^11^ and assisting emergency management.^12^ Over the past few decades, various models^13^^,^^14^^,^^15^^,^^16^^,^^17^^,^^18^^,^^19^ have been proposed to explain the universal laws of human mobility. Classical models focus on predicting human mobility at different scales, such as the gravity model (GM),^20^ radiation model^16^ (RM), population-weighted opportunities model (PWO),^21^ and related extension models.^22^^,^^23^ In addition, fine-grained models have been developed to capture individual micro-level travel behavior, such as the exploration and preferential return model (EPR),^15^ container model,^24^ and universal model of individual and population (UMIP).^25^

Human mobility within urban agglomerations encompasses a wide range of spatial scales, such as intracity, intercity, and interstate. The dynamics of travel behavior are implicitly confined by geographical boundaries, administrative divisions, and transportation facilities,^26^^,^^27^ and individuals exhibit distinct mobility patterns across different spatial scales.^28^ Notably, additional scales raise the challenge of modeling human mobility by adding, for example, higher computational dimensions, and more complicated travel choices. With a few exceptions,^24^^,^^29^ existing studies^3^^,^^30^^,^^31^ have been limited to analyzing and predicting either single-scale intercity or intracity mobility within urban agglomerations. However, when modeling human mobility between communities in different cities, which involves both city and community scales, the number of included regions dramatically expands according to the number of cities or communities. Moreover, it is challenging to characterize complex multi-scale travel patterns using traditional models (e.g., GM and RM). For example, owing to the constraints of administrative divisions, the travel probability between two neighboring communities in the same city (i.e., intra-city travel) may deviate considerably from that between two neighboring communities in different cities (i.e., inter-city travel). When an individual selects a county for travel, the subsequent selection of communities within the target county may be slightly influenced by the distance from the origin. In this multi-scale mixed travel scenario, GM’s distance decay effect and RM’s intervening opportunity effect become less effective. These inherent challenges^22^^,^^32^ underscore the necessity and complexity of multi-scale unified human mobility modeling.

From a methodological perspective, existing studies^22^^,^^23^ have mainly focused on analyzing the effect of geographical features (e.g., population, points of interest, and distance) on human travel behavior to improve mobility prediction accuracy rather than on designing and expressing human-like behavior selection mechanisms. For example, people’s mental representations of physical space show a clear hierarchical structure^33^ embodied in the characterization, judgment, and selection of spatial regions.^34^^,^^35^ We usually describe the spatial location of a place hierarchically in a nested organizational structure using typical spatial scales, such as state, county (city), community, and street information.

To address these issues, we propose a multi-scale unified model (MSUM) to achieve a unified human mobility prediction at multiple spatial scales in urban agglomerations. To characterize the human hierarchical travel choice process, MSUM embeds a cascaded multi-class classifier based on deep convolutional neural networks^36^ (CNNs), which is an extension of the nested logit (NL) model.^37^ Individual historical memory and population attractiveness features are used as classifier inputs, and by adding convolutional layers and nonlinear activation layers, complex human mobility features can be automatically extracted. Furthermore, to derive population attractiveness features, we introduce a graph generative adversarial network^38^ (GGAN) to extract the trip distribution at single spatial scales (e.g., county and community scales). Unlike traditional mobility models (e.g., GM and PWO), which are based on distance or intervening opportunity, we formulate trip distribution prediction as a missing data imputation problem. In GGAN, the generator is used to impute the missing travel probabilities between locations with unknown travel volumes. The discriminator is used to discriminate between observable and missing components. Through adversarial training between the two, the nonlinear dependencies between travel probability and urban indicators (e.g., distance and population) can be extracted. We thereby calculate the population attractiveness of the target location by summing its probabilities of being visited by other locations, as generated by GGAN. Finally, by training MSUM using empirical human mobility data, the model can simultaneously capture the scaling laws of individuals and populations across multiple spatial scales within urban agglomerations, aligning well with empirical findings.

## Results

### MSUM for human mobility prediction in urban agglomerations

**Figure 1:**
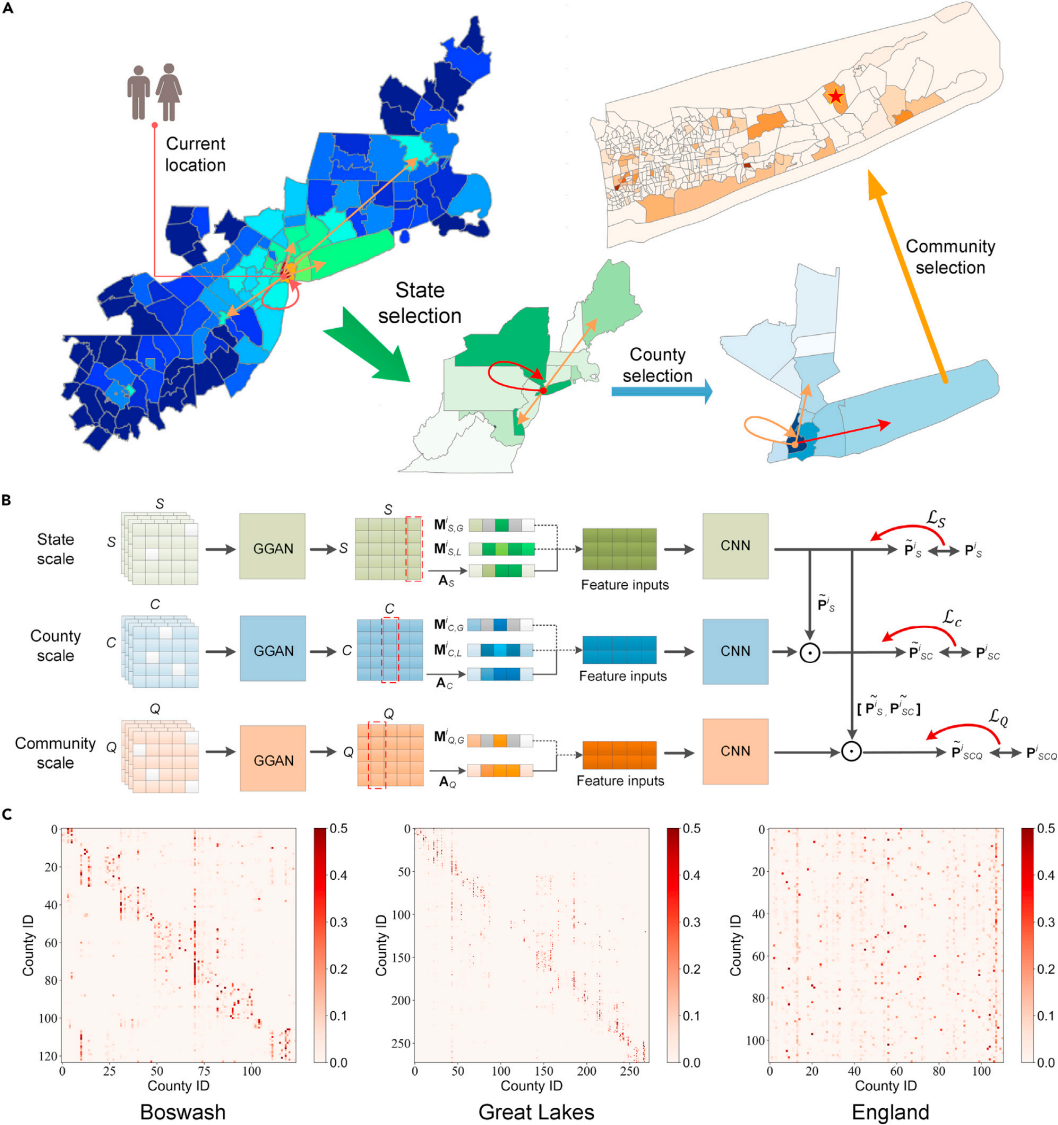
Multi-scale unified model of human mobility in urban agglomerations (A) Individual’s hierarchical travel choice process. Individuals make travel choices hierarchically from the large (e.g., state) to the small scale (e.g., community) following the corresponding choice probability. The color of each region indicates the corresponding selection probability. (B) Multi-scale unified model architecture. The model’s input includes individual memory features ($M_{L}^{i}$ and $M_{G}^{i}$) and population attractiveness features (A). The population attractiveness feature is obtained by the graph generative adversarial network (GGAN) (see Note S3 for details). Based on the feature extraction of a deep convolutional neural network (CNN), the model outputs the classification probability of different spatial scales layer by layer. The classification probability of the upper layer of the model multiplies the probability of the lower layer to achieve conditional constraints. The classification error of the lower layer propagates to the upper layer to participate in updating CNN parameters. (C) Travel probabilities between counties in three urban agglomerations predicted by GGAN. Darker colors indicate greater inter-county travel probability.

MSUM is inspired by the classical NL model^37^ based on the random utility theory to model individual choice behavior. The NL model creates a nested structure (see Figure S2 and Note S1 for details) to depict the correlation between alternatives and to avoid independence of irrelevant alternatives in the traditional logit model.^39^ Each layer of the NL model can be regarded as a multinomial logit (MNL) model^40^ with different attributes linked by conditional probability and utility feedback. Studies^41^ have shown that an MNL model is equivalent to a CNN with X as the feature input, β as the filter parameter, and *softmax*^42^ as the nonlinear activation function. This finding led us to extend the original NL model to a deep learning-based cascaded multi-class classification model (the MSUM, Figure 1) to achieve deep extraction of nonlinear mobility features and unified prediction of multi-scale human mobility in urban agglomerations.

In MSUM, individual travel choice at multiple spatial scales within urban agglomerations is regarded as the process of hierarchically selecting the correct class with the greatest utility from candidate locations (Figure 1A). That is, the urban agglomeration can be divided into different spatial scales (e.g., state, county, and community, depending on dataset granularity; see data description and preprocessing for details). The individual first selects a class (state) in the large-scale (e.g., state scale) regional candidate set, and then selects a medium-scale class (e.g., county) under the selected region to travel. Meanwhile, the large-scale region nests all corresponding small-scale regions. The selection (classification) process at different spatial scales is completed using different deep learning-based classifiers (Figure 1B; see implementation of MSUM and Note S2 for details), which are mutually constrained by the classification probability and classification error. The feature input of the classifier includes the individual memory and population attractiveness features. The memory feature indicates that individuals strongly prefer to return to locations they have visited before, and the degree of this preference is proportional to the frequency of visits.^15^ The attractiveness feature indicates that a location’s popularity is proportional to the overall probability of being visited—an attractive location is visited by more people from other locations. We divide individual memory features into global memory features ($M_{G}^{i}$) and local memory features ($M_{L}^{i}$). Taking the county scale (i.e., scale C) as an example, the global memory feature $M_{C,G}^{i}$ represents the visit frequency of individual i to other counties, regardless of the origin county. The local memory feature $M_{C,L}^{i}$ represents the visit frequency of individual i to other counties from the current county. To obtain the population attractiveness feature vector $A_{C}$, we propose a GGAN (see Note S3 and Figure S1 for details) to predict human trip distribution at different spatial scales. GGAN adversarially learns the parameters of the graph neural network through the two core tasks of data imputation and missing discrimination, and outputs the travel probability between each county (Figure 1C). The generator aims to impute missing travel probabilities between counties based on existing observation data, whereas the discriminator aims to distinguish which travel probabilities are generated and which are observed. Finally, the county’s attractiveness can be obtained by accumulating the travel probabilities from other counties to the target county.

### Population attractiveness feature extraction

**Figure 2:**
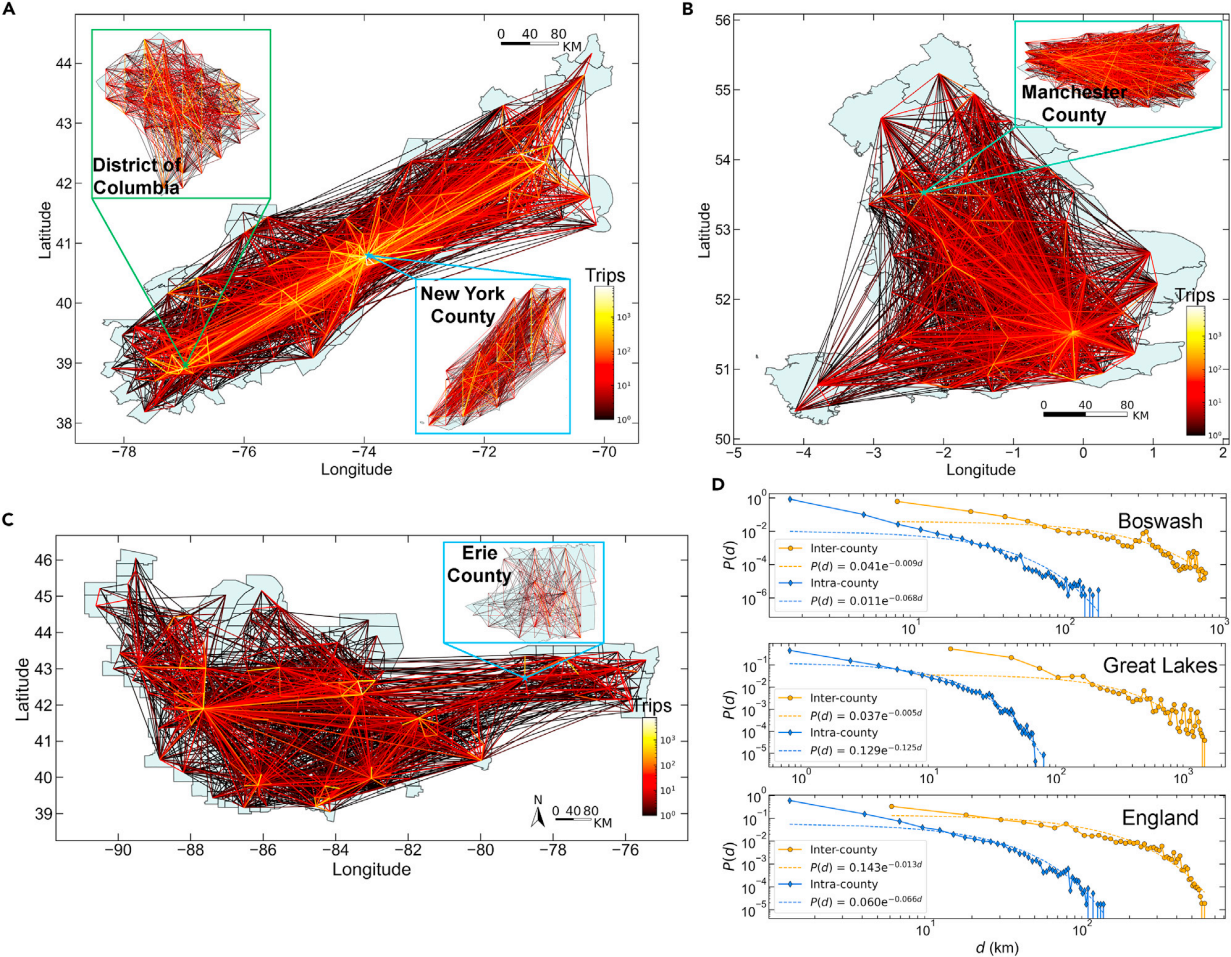
Human mobility networks of three urban agglomerations (A) Distribution of human mobility among 124 counties in the Boston-Washington (Boswash) urban agglomeration, USA. (B) Distribution of human mobility among 111 counties in the England urban agglomeration of the UK. (C) Distribution of human mobility among 273 counties in the Great Lakes urban agglomeration, USA. Line color indicates the intensity of the travel flows between regions, with brighter (darker) colors indicating stronger (weaker) travel flows. (D) Travel distance distribution of inter-county and intra-county trips in the three urban agglomerations.

GGAN is proposed to accurately and robustly predict the single-scale (i.e., inter-county and intra-county) human trip distribution in urban agglomerations, and it can provide more reliable population attractiveness features for MSUM compared with benchmark models (i.e., GM, RM, and PWO; see supplemental experimental procedures for model details). We conduct a series of comparison experiments in three world-class urban agglomerations: Boston-Washington (Boswash) (USA), Great Lakes (USA), and England (UK) urban agglomerations. These three urban agglomerations encompass different historical, cultural, educational, and economic contexts, which can provide diverse and comprehensive perspectives for understanding mobility patterns within urban agglomerations. They cover 124 (Boswash) and 273 (Great Lakes) counties in the USA and 111 (England) in the UK. We regard *county* as the primary spatial scale in the urban agglomeration, and further divide the corresponding counties into 7,731, 15,156, and 10,114 non-overlapping square regions according to their area to represent the secondary spatial scale (i.e., *community* scale). The mobility data for the Boswash and Great Lakes urban agglomerations are user check-in data from the website Weeplaces,^43^ which consists of travel traces of 4,952 and 2,033 individuals, respectively. The mobility data for the England urban agglomeration are the user check-in data from the location-based social networking website Gowalla,^44^ which consists of travel traces of 3,193 individuals (see data description and preprocessing for details). Because of the coverage of the mobile data used, we selected three of the top 5 world-class urban agglomerations^45^^,^^46^ as the research areas. Nonetheless, our proposed model can be generalized to urban agglomerations worldwide. The human mobility networks in the three urban agglomerations are shown in Figure 2. People’s mobility patterns at different spatial scales have obvious heterogeneity and hierarchy. The intra-county travel network (i.e., inter-community travel) is more intensive than the inter-county travel network. Travel distance characteristics at different scales are shown in Figure 2D.

**Figure 3:**
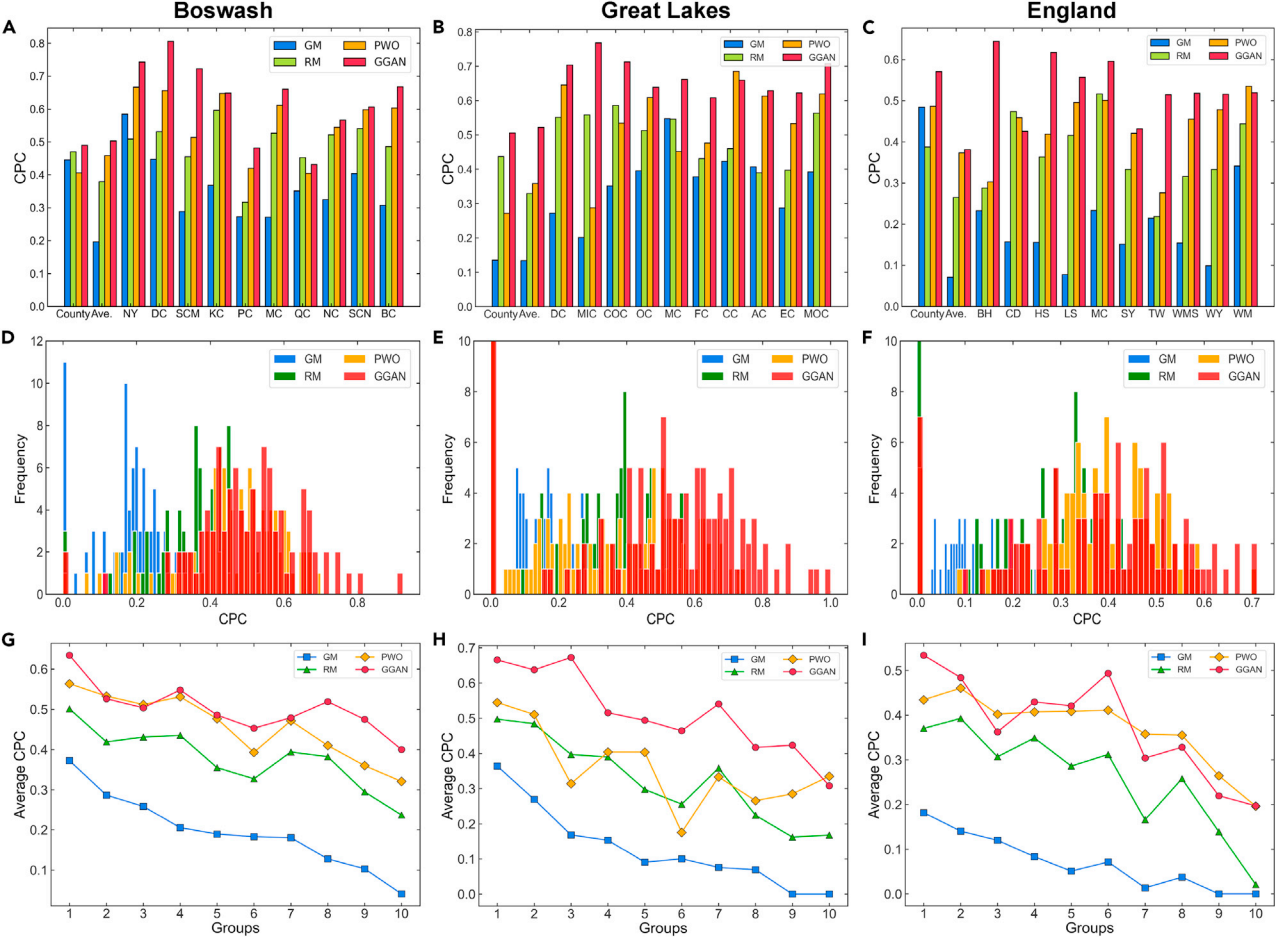
Performance comparison of single-scale human mobility prediction in terms of common part of commuters (A–C) Performance comparison of the gravity model (GM), radiation model (RM), population-weighted opportunities model (PWO), and graph generative adversarial network (GGAN) at the county and community scales. The first column (labeled “County”) indicates the model’s accuracy in predicting travel flows between counties. The second column (labeled “Ave.”) indicates the average accuracy of the model in predicting inter-community travel flows for all counties within the urban agglomeration. The last 10 columns (labeled by county abbreviation) represent the inter-community travel flow prediction accuracy of GM, RM, PWO, and GGAN in the 10 counties with the highest travel volume. (D–F) Distribution of common part of commuters values for travel flow prediction at the community scale. (G–I) Prediction accuracy of GM, RM, PWO, and GGAN in different active regions. In each urban agglomeration, all counties are evenly divided into 10 groups and ranked in descending order based on travel volume. Each point indicates the average prediction accuracy of the corresponding model under each group.

We use the random missing method to construct the training and test sets for model comparison (see model training and evaluation for details). Figure 1C shows the travel probabilities between counties in the three urban agglomerations as predicted by GGAN. We find that travel between counties in the Boswash and Great Lakes urban agglomerations is greatly affected by state borders, and there is a clear hierarchical organization. For the England urban agglomeration, counties are the first-level administrative divisions, so travel between counties is more uniform. Using the common part of commuters^22^^,^^25^ (CPC) to quantify the similarity between real and predicted flows, Figure 3 shows that GGAN has the best prediction performance at different spatial scales in the three urban agglomerations. Note that CPC values range between 0 and 1, with higher values indicating a better match between the predicted and actual flows. For a more comprehensive model comparison, we use the mean absolute error and root mean-square error (RMSE) to evaluate model performance (see supplemental experimental procedures and Tables S1–S3 for details). As shown in Figures 3A–3C, the first column (labeled “County”) shows the performance evaluation results of travel flow prediction between counties in urban agglomerations. For the Great Lakes urban agglomeration, the CPC value of GGAN (CPC = 0.505) is 0.370, 0.069, and 0.234 higher than those of GM, RM, and PWO, respectively (see Table S2 for details).

**Figure 4:**
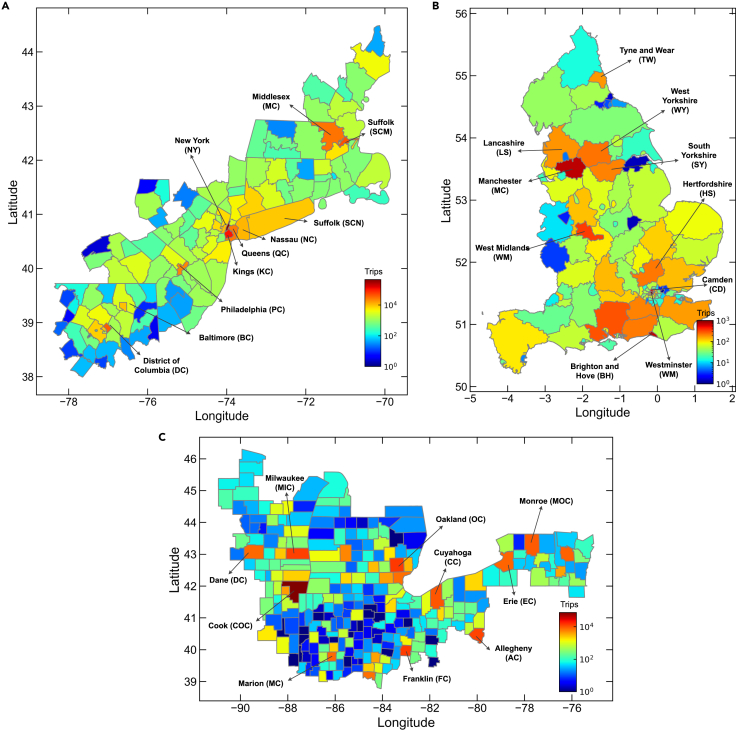
Selection of the top 10 counties in the three urban agglomerations regarding travel volume Top 10 counties in (A) Boswash, (B) England, and (C) Great Lakes urban agglomerations. Letters in parentheses indicate county name abbreviations. The color of each region indicates the number of trips originating from the corresponding region, including intra- and inter-county trips.

The second column (labeled “Ave.”) shows the average performance evaluation results of the inter-community travel flow predictions for all counties. Similar to the county scale, the overall prediction performance of GGAN is better than that of the other two baseline models. The distribution of CPC values for each model at the community scale is shown in Figures 3D–3F. As shown in the last 10 columns (labeled by county abbreviation) of Figures 3A–3C, we select the top 10 counties (Figure 4) in the three urban agglomerations in terms of travel volume to visualize each model’s prediction performance at the community scale. Most selected counties (e.g., New York, Cook, Manchester, and Birmingham) are core political, economic, and cultural areas in the USA and the UK. The free flow of various production factors in these counties has promoted the rapid development of urban agglomerations. For a performance comparison, GGAN has the highest prediction accuracy, and is significantly better than the baseline models. For instance, in the District of Columbia (labeled “DC”) of the Boswash urban agglomeration (see Table S1 for details), the CPC value of GGAN (CPC = 0.806) is 0.358, 0.275, and 0.150 higher than those of GM, RM, and PWO, respectively.

To investigate the model’s prediction performance in different active regions, we divide the counties in each urban agglomeration into 10 groups based on county travel volume and rank them in descending order. We then calculate the prediction performance of the trip distribution at the community scale for each group (see Figures 3G–3I and Table S4 for details). In the three urban agglomerations, the prediction performance of all models degrades with a decrease in travel volume. This is because trip distribution becomes sparse and random in low-activity counties, making it difficult for the model to accurately capture mobility features based on current travel information. Nevertheless, GGAN maintains remarkable prediction accuracy across groups and significantly outperforms the baseline models.

### Multi-scale human mobility prediction

MSUM can predict multi-scale mobility patterns within urban agglomerations. We use a portion of actual individual traces to train the model (see model training and evaluation for details) and simulate the subsequent continuous individual traces (see simulation of individual traces for details). At each step, an individual selects a community within a specific county for travel based on the MSUM classification results. The degree of authenticity of the individual and population mobility patterns captured by MSUM can be verified by comparing the simulated traces with unseen data. To explore the accuracy of the model, the EPR model,^15^ three EPR variant models,^17^^,^^47^^,^^48^ and the UMIP model are selected for performance comparison (see supplemental experimental procedures for details).

**Figure 5:**
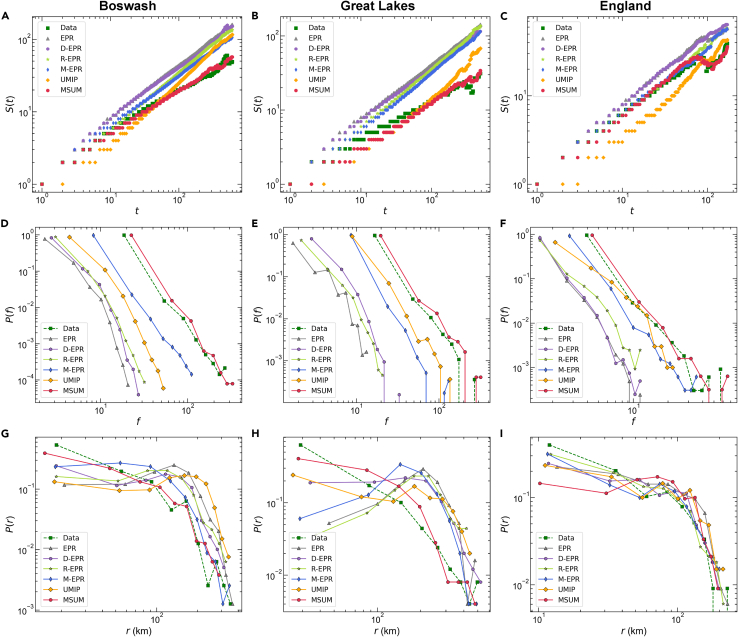
Multi-scale human mobility prediction in urban agglomerations at the individual level (A–C) Distribution of the total number of locations visited in t trips. (D–F) Frequency distribution of individuals visiting a location. (G–I) Radius of gyration distribution. All distributions are calculated from the simulated (see simulation of individual traces for details) and actual individual traces.

At the individual level, we focus on three essential scaling laws (Figure 5): the total number of locations visited in t trips, the frequency distribution of individuals visiting a location, and the distribution of the radius of gyration.^14^ For the three urban agglomerations, the empirical data (green) in Figures 5A–5C show that the number of locations visited by individuals increases algebraically rather than exponentially over time owing to the influence of the individual memory effect. In a travel environment involving multiple spatial scales, our model (red) precisely matches the empirical results, whereas the other baseline models overestimate them even though all consider the individual’s historical memory. Accordingly, Figures 5D–5F show the frequency distribution of visits to a location, which is power-law decreasing. Consistent with empirical research,^17^ people visit only a few locations regularly, such as home and work. Again, MSUM provides a scale description that is more consistent with the empirical distribution, whereas the baseline models underestimate the visiting frequency. Among the baseline models, UMIP performs best for the Great Lakes and England urban agglomerations, demonstrating the necessity of considering population travel information in multi-scale mobility modeling in addition to individual memory features. Figures 5G–5I show an exponentially decreasing trend in the radius of gyration. A comparison of the different models shows that our model can more accurately describe individual travel preferences, whether short or long distance. Moreover, by aggregating the simulation traces of all individuals, individual mobility patterns at a single scale (e.g., inter-county travel) can be quantified (see supplemental experimental procedures, Figures S5–S7, and Table S6 for details).

**Figure 6:**
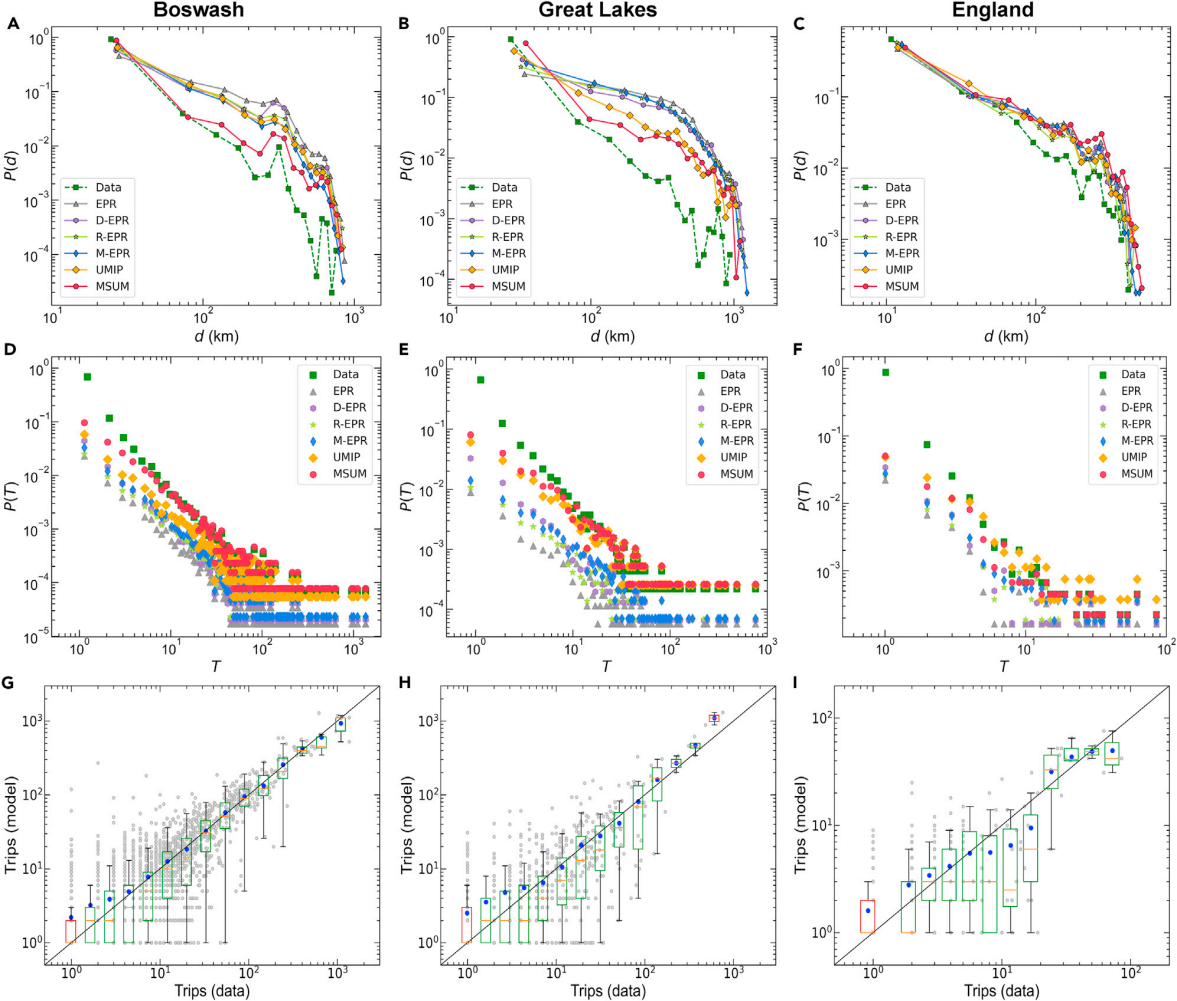
Multi-scale human mobility prediction in urban agglomerations at the population level (A–C) Predicted and real distributions of travel distance. (D–F) Predicted and real distributions of the number of trips between two locations. (G–I) Paired comparison of predicted and real trips. Gray points indicate observed and predicted location pairs. Blue points indicate the average number of predicted trips in different bins. The boxplot indicates the distribution of the number of predicted trips in different bins of the number of observed trips. A box is green if the black line y=x is between the 5th and 95th percentiles of the box, and red otherwise. To characterize the model’s prediction performance at the population level, all distributions are calculated by aggregating simulated and actual individual traces.

At the population level, we quantify the prediction performance of MSUM using two scaling laws (Figure 6): the travel distance distribution of the population, and the trip number distribution between two locations. Figures 6A–6C show the probability distribution of people traveling different distances, which decreases exponentially. Our model has good prediction performance for short trips and a slight overestimation for long trips, but it is a significant improvement over the baseline models. Figures 6D–6F show another important scaling law describing the probability distribution of the number of trips between two locations. Compared with the baseline model, our model can robustly predict the scaling laws of algebraic decay in the three urban agglomerations and is in excellent agreement with the empirical distribution. The baseline model significantly underestimates the number of trips between locations, except for UMIP. In addition, as shown in Figures 6G–6I, a pairwise comparison of the predicted trips with the observed trips shows that the model-predicted and actual trips are statistically indistinguishable (see Figure S4 for details). The effectiveness measures show that the CPC values of MSUM in the three urban agglomerations are 0.294 (Boswash), 0.098 (Great Lakes), and 0.098 (England) higher than that of the optimal baseline model (see Table S5 for details). For instance, compared with the best-performing memory EPR (M-EPR) model for the Boswash urban agglomeration, the CPC and RMSE values of MSUM increased by 77.78% and 44.55%, respectively. Based on the above scale laws and model performance analysis, our model can effectively predict multi-scale travel patterns within urban agglomerations by simulating the hierarchical travel choice behavior of human beings, and coupling individual memory and population attractiveness features, thereby improving the accuracy and robustness of travel prediction.

## Discussion

We propose MSUM to predict human mobility in urban agglomerations at multiple spatial scales. By considering the hierarchical nature of human travel choice behavior, our model effectively reproduces the scaling laws of individual and population mobility between communities in different counties in urban agglomerations. In our model, GGAN can accurately and robustly predict the single-scale (i.e., inter- and intra-county) mobility patterns of different active regions compared with state-of-the-art population mobility models, and it provides a reliable characterization of location attractiveness. For the mobility prediction at multiple spatial scales, taking the Great Lakes urban agglomeration as an example, the human activity space increases from 273 to 15,156 regions. This increase of spatial scale leads to sparsity of data and uncertainty of human activities, amplifying the difficulty of accurate mobility prediction. By simulating the human behavior selection mechanism (i.e., hierarchical mental representation of physical spaces) to construct a cascaded multi-class classification framework, and leveraging the powerful feature extraction capability of deep neural networks, our MSUM can implicitly reduce the solution space of travel choice layer by layer, thereby accurately capturing various scaling laws in accordance with empirical data, such as the radius of gyration distribution, trip distance distribution, and trip number distribution between two locations.

As the main form of settlement in modern human society, understanding the multi-scale mobility patterns of individuals and populations within urban agglomerations can support decision-making for various urban settings. In particular, our model can precisely describe travel behavior at the individual level, unlocking unprecedented possibilities for refined realistic simulation, epidemic prevention and control prediction, regional development, urban planning, transportation planning, etc. For example, the quantitative evaluation results demonstrate that our model can predict travel flow between large-scale regions (e.g., over 10,000 regions) more accurately than state-of-the-art individual mobility models. Regarding infrastructure construction, urban decision-makers can identify underdeveloped areas within the urban agglomeration based on the frequency of travel visits between regions and make direct investments to promote balanced growth and development. Simultaneously, the multi-scale mobility prediction results help identify popular tourist destinations and routes for tourism-driven cities. By understanding where individuals spend the most time, tourism managers can focus on improving infrastructure and providing better services, such as providing more passenger corridors or strengthening measures in regions with frequent travel to avoid congestion caused by a large number of trips. Regarding transportation planning, trip distribution prediction results at different spatial scales can assist managers in building intelligent transportation systems. The prediction results of short-distance trips can help optimize the design and operation of public transportation lines within urban agglomerations, ensure that public transportation services are close to citizens' actual needs, and improve public transportation system efficiency and convenience. With long-distance trip distribution prediction results, managers can formulate and optimize transportation networks based on travel needs and recommend multi-modal travel services^49^ to improve travel efficiency. Moreover, by analyzing the multi-scale travel flow patterns in urban agglomerations, urban planners can anticipate regions likely to experience significant population growth and urban sprawl. This information is vital for making informed decisions on zoning, land-use regulations, and urban expansion plans, ensuring that new developments align with the growing population’s needs.

Our study has some limitations that should be addressed in future research. MSUM extends the classical NL model by constructing a cascaded deep CNN framework to simulate and express the mechanism of human hierarchical travel choices. The interpretability of deep neural networks remains a key challenge in advancing the understanding of probabilistic relationships between input features (e.g., individual memory and population attractiveness) and corresponding travel choices. The abstract feature information extracted by GGAN and deep CNN can be analyzed by future studies using game theory, agnostic,^50^^,^^51^ and other techniques. In addition, we did not consider the effects of finer administrative boundaries and geographical contextual features (e.g., points of interest) on human travel. However, these features are closely related to people’s daily movement and can be used as the feature input of the prediction model in future research.

## Experimental procedures

### Resource availability

#### Lead contact

Further information and requests for resources and reagents should be directed to and will be fulfilled by the lead contact, Xiqun (Michael) Chen (chenxiqun@zju.edu.cn).

#### Materials availability

This study did not generate new unique materials.

### Data description and preprocessing

Regional boundary data for the Boswash, Great Lakes, and England urban agglomerations were provided by the American Regional Planning Association^53^ and the European Union.^54^ The Boswash and Great Lakes urban agglomerations cover 124 and 273 counties in the USA, respectively. The England urban agglomeration covers 111 counties. County-scale administrative boundary data were downloaded from the GADM open-source platform (https://gadm.org/index.html). To represent the finer scale regions (i.e., community scale), we used the Python library scikit-mobility^55^ to divide each county into multiple non-overlapping square regions. Assuming that the area of a county is A, the side length of each square region is set to $\sqrt{A}/5$, which ensures uniformity of the fine-scale boundary division. Thus, the three urban agglomerations contain 7,731 (Boswash), 15,156 (Great Lakes), and 10,114 (England) communities.

All experimental analyses were based on two check-in datasets collected from publicly available location-based social networks. The first dataset was collected by Gowalla^44^ and consists of 36,001,959 check-ins generated by 319,063 users worldwide from February 2009 to October 2010. The second dataset was collected by Weeplaces,^43^ which integrates the application programming interfaces of multiple location-based social networking services (e.g., Foursquare and Gowalla) and consists of 7,658,368 check-ins of 15,799 users from November 2003 to June 2011. We filter successive points that repeatedly stay at the same location and users who travel too little to avoid data interference. After mapping check-in coordinates to the corresponding urban agglomerations, Boswash contains 1,016,454 trips from 4,952 users, Great Lakes contains 285,089 trips from 2,033 users, and England contains 111,772 trips from 3,193 users. The trip distribution characteristics of the individual traces are shown in Figure S3. Moreover, population data are publicly available from the United States Census Bureau^56^ and the Office for National Statistics.^57^

### MSUM implementation

The specific implementation process of MSUM includes the following three steps (Figure 1B).

(1) Individual memory and population attractiveness features are concatenated as feature inputs of MSUM for feature extraction. Specifically, to consider an individual’s current location dynamically, we divide the individual memory features into global ($M_{G}^{i}$) and local memory features ($M_{L}^{i}$). The global memory feature represents the historical visit frequency of individual i to other locations regardless of the origin. The local memory feature represents the historical visit frequency of individual i from the current location to other locations. To obtain the population attractiveness feature vector $A_{C}$, a GGAN is proposed to generate a trip distribution matrix at different scales. The location’s attractiveness can be obtained by accumulating the travel probabilities from other locations to the target location.
(2) The concatenation vectors are input into CNN (see Note S2 for details) without bias parameters. As the input feature is a two-dimensional matrix, the one-dimensional convolution filter is used to construct CNN for feature extraction. At different spatial scales, CNN has a similar network framework, but has different network parameters.
(3) The model output is a vector representing the classification results at the corresponding scale. The vector length equals the total number of classes under the corresponding scale; the smaller the scale, the larger the vector length. To associate the selection results of different scales, the classification probability output of the upper CNN is multiplied by that of the lower CNN to achieve the constraint of hierarchical selection. For example, when an individual has a low probability of selecting a county, s/he is less likely to select a community within that county. Moreover, the classification error of the lower-layer CNN propagates to the upper layer, affecting and participating in updating the network parameters of the upper-layer CNN.

### Simulation of individual traces

Given the trained MSUM, we can simulate individual travel traces using the following three steps.

(1) Assuming that the total number of movement steps of individual i in the empirical test dataset is $L^{i}$, we use the first ${2L}^{i}/3$ movement steps to calculate the historical memory features of the individual at different spatial scales, including global $M_{G}^{i}$ and local memory features $M_{L}^{i}$. Based on the trained GGAN, population attractiveness feature A at different spatial scales can also be obtained.
(2) The location after ${2L}^{i}/3$ movement steps is taken as the initial location of individual i. The local memory feature vector starting from the current location, global memory feature vector, and population attractiveness feature vector are concatenated and input into MSUM. Individual i chooses a location to travel in the multi-scale space of urban agglomerations by referring to the multi-scale selection probability distribution output by MSUM.
(3) The individual’s historical memory feature vector is updated. Repeat step (2) to complete the individual trace simulation with ${2L}^{i}/3$ movement steps.

### Model training and evaluation

For GGAN, we construct a test set by setting a random missing rate of 20% to mask the travel volumes between some locations as missing data. For the non-missing 80% of the data, we generate sufficient training samples by repeatedly performing random masking of 10% of the travel volume information. GGAN is trained for 1,000 epochs using the Adam optimizer^58^ with a learning rate of 0.001. To compare the model performance, state-of-the-art GM,^22^ RM,^16^ and PWO^21^ are implemented as baseline models (see supplemental experimental procedures for details). For MSUM, we use a stratified sampling method to construct the training and test sets with a ratio of 8:2 based on the individual’s movement steps. For each individual, we use the first two-thirds of the data to calculate the individual’s initial memory features, and sequentially update the memory features based on the empirical data to generate training samples, during which the real destination is used as the training label of the model. The entire MSUM is trained for 30 epochs using the Adam optimizer, with a learning rate of 0.001. The cross-entropy function is used as the loss function to evaluate the performance of the network parameters. For a more comprehensive comparison, four individual mobility models are also implemented as baseline models (see supplemental experimental procedures for details): EPR, gravity EPR^17^ (D-EPR), recency EPR^47^ (R-EPR), M-EPR^48^, and UMIP.^25^

## Data Availability

The mobility data are publicly available at http://snap.stanford.edu/data/loc-gowalla.html and https://www.yongliu.org/datasets/. National administrative area data for the USA and UK are publicly available at https://gadm.org/maps.html. Population data for the USA and UK are publicly available at https://www.census.gov/quickfacts/fact/table/US/POP010210 and https://www.ons.gov.uk/visualisations/censuspopulationchange/, respectively. The MSUM source code is available from Zenodo (https://doi.org/10.5281/zenodo.8175023).^52^ The other data relevant to this study are available upon request.

## Supplemental information

Document S1. Notes S1–S3, Tables S1–S6, Figures S1–S7, and supplemental experimental procedures

Document S2. Article plus supplemental information

## References

[1] Fang C. (2014). Progress and the future direction of research into urban agglomeration in China. Acta Geograph. Sin..

[2] Gottmann J. (1964).

[3] He Z. (2020). Spatial-temporal fractal of urban agglomeration travel demand. Physica A.

[4] Fang C., Yu D. (2017). Urban agglomeration: An evolving concept of an emerging phenomenon. Landsc. Urban Plann..

[5] MAGNO R.A. (1972). Metropolitan region planning and development in Japan. Roy. Aust. Plann. Inst. J..

[6] Lang R.E., Nelson A.C. (2007).

[7] Fang C. (2015). Important progress and future direction of studies on China's urban agglomerations. J. Geogr. Sci..

[8] Paasi A. (2004). Place and region: Looking through the prism of scale. Prog. Hum. Geogr..

[9] Ferreira C.P., Marcondes D., Melo M.P., Oliva S.M., Peixoto C.M., Peixoto P.S. (2021). A snapshot of a pandemic: The interplay between social isolation and covid-19 dynamics in Brazil. Patterns.

[10] Liu L., Wang H., Zhang Z., Zhang W., Zhuang S., Wang S., Silva E.A., Lv T., Chio C.O., Wang Y. (2022). Infectiousness of places–Impact of multiscale human activity places in the transmission of COVID-19. NPJ Urban Sustain..

[11] Dong L., Li R., Zhang J., Di Z. (2016). Population-weighted efficiency in transportation networks. Sci. Rep..

[12] Fan Z., Song X., Shibasaki R. (2020). In Big Data in Emergency Management: Exploitation Techniques for Social and Mobile Data.

[13] Brockmann D., Hufnagel L., Geisel T. (2006). The scaling laws of human travel. Nature.

[14] González M.C., Hidalgo C.A., Barabási A.L. (2008). Understanding individual human mobility patterns. Nature.

[15] Song C., Koren T., Wang P., Barabási A.L. (2010). Modelling the scaling properties of human mobility. Nat. Phys..

[16] Simini F., González M.C., Maritan A., Barabási A.L. (2012). A universal model for mobility and migration patterns. Nature.

[17] Pappalardo L., Simini F., Rinzivillo S., Pedreschi D., Giannotti F., Barabási A.L. (2015). Returners and explorers dichotomy in human mobility. Nat. Commun..

[18] Reia S.M., Rao P.S.C., Ukkusuri S.V. (2022). Modeling the dynamics and spatial heterogeneity of city growth. npj Urban Sustain..

[19] Schläpfer M., Dong L., O'Keeffe K., Santi P., Szell M., Salat H., Anklesaria S., Vazifeh M., Ratti C., West G.B. (2021). The universal visitation law of human mobility. Nature.

[20] Zipf G.K. (1946). The P_1_ P_2_/D hypothesis: On the intercity movement of persons. Am. Socio. Rev..

[21] Yan X.Y., Zhao C., Fan Y., Di Z., Wang W.X. (2014). Universal predictability of mobility patterns in cities. J. R. Soc. Interface.

[22] Barbosa H., Barthelemy M., Ghoshal G., James C.R., Lenormand M., Louail T., Menezes R., Ramasco J.J., Simini F., Tomasini M. (2018). Human mobility: Models and applications. Phys. Rep..

[23] Simini F., Barlacchi G., Luca M., Pappalardo L. (2021). A deep gravity model for mobility flows generation. Nat. Commun..

[24] Alessandretti L., Aslak U., Lehmann S. (2020). The scales of human mobility. Nature.

[25] Yan X.Y., Wang W.X., Gao Z.Y., Lai Y.C. (2017). Universal model of individual and population mobility on diverse spatial scales. Nat. Commun..

[26] Cadwallader M.T. (1992).

[27] Thiemann C., Theis F., Grady D., Brune R., Brockmann D. (2010). The structure of borders in a small world. PLoS One.

[28] Berry B.J.L. (1967).

[29] Han X.P., Hao Q., Wang B.H., Zhou T. (2011). Origin of the scaling law in human mobility: Hierarchy of traffic systems. Phys. Rev. E E..

[30] Wang Z., Ye X., Lee J., Chang X., Liu H., Li Q. (2018). A spatial econometric modeling of online social interactions using microblogs. Comput. Environ. Urban Syst..

[31] Fang C., Yu X., Zhang X., Fang J., Liu H. (2020). Big data analysis on the spatial networks of urban agglomeration. Cities.

[32] Barthélemy M. (2011). Spatial networks. Phys. Rep..

[33] Hirtle S.C., Jonides J. (1985). Evidence of hierarchies in cognitive maps. Mem. Cognit..

[34] Stevens A., Coupe P. (1978). Distortions in judged spatial relations. Cognit. Psychol..

[35] Wilton R.N. (1979). Knowledge of spatial relations: The specification of the information used in making inferences. Q. J. Exp. Psychol..

[36] Goodfellow I., Bengio Y., Courville A. (2016).

[37] Williams H.C.W.L. (1977). On the formation of travel demand models and economic evaluation measures of user benefit. Environ. Plann..

[38] Goodfellow I. (2014). Generative adversarial nets.

[39] Lute R.D. (1959).

[40] Train K.E. (2009).

[41] Sifringer B., Lurkin V., Alahi A. (2020). Enhancing discrete choice models with representation learning. Transp. Res. Part B Methodol..

[42] Bishop C.M. (1995).

[43] Liu Y., Wei W., Sun A., Miao C. (2014). Proc. 23rd ACM International Conference on Information and Knowledge Management.

[44] Cho E., Myers S.A., Leskovec J. (2011). Proc. 17th ACM SIGKDD International Conference on Knowledge Discovery and Data Mining.

[45] Yang L., Zhao P., Liu B., Gao Y., Zhou H., Li Q., Jiang Y., Yang Z. (2022). Network patterns of zhongyuan urban agglomeration in China based on baidu migration data. Ann. Transl. Med..

[46] Kii M. (2021). Projecting future populations of urban agglomerations around the world and through the 21st century. npj Urban Sustain..

[47] Barbosa H., de Lima-Neto F.B., Evsukoff A., Menezes R. (2015). The effect of recency to human mobility. EPJ Data Sci..

[48] Alessandretti L., Sapiezynski P., Sekara V., Lehmann S., Baronchelli A. (2018). Evidence for a conserved quantity in human mobility. Nat. Human Behav..

[49] Meng L., Somenahalli S., Berry S. (2020). Policy implementation of multi-modal (shared) mobility: Review of a supply-demand value proposition canvas. Transport Rev..

[50] Štrumbelj E., Kononenko I. (2014). Explaining prediction models and individual predictions with feature contributions. Knowl. Inf. Syst..

[51] Lundberg S.M., Lee S.I. (2017). Proc. 30th Conference on Neural Information Processing Systems.

[52] Chen Y. (2023).

[53] American Regional Planning Association (2007). https://rpa.org/work/reports?series=america-2050.

[54] European Union (2001). https://en.wikipedia.org/wiki/ESPON_metropolitan_areas_in_the_United_Kingdom.

[55] Pappalardo L., Simini F., Barlacchi G., Pellungrini R. (2019). Scikit-mobility: A Python library for the analysis, generation and risk assessment of mobility data. arXiv.

[56] United States Census Bureau (2010). https://www.census.gov/quickfacts/fact/table/US/POP010210.

[57] Office for National Statistics (2011). https://www.ons.gov.uk/.

[58] Kingma D.P., Ba J. (2015). Adam: A method for stochastic optimization. arXiv.

