# Supplementary material for: A multi-scale unified model of human mobility in urban agglomerations
Source: Patterns (N Y). 2023 Oct 17;4(11):100862. doi: 10.1016/j.patter.2023.100862 (PMC10682749; doi:10.1016/j.patter.2023.100862)
Supplement: Document S1. Notes S1–S3, Tables S1–S6, Figures S1–S7, and supplemental experimental procedures [file mmc1.pdf]

Patterns, Volume *4*

Supplemental information

A multi-scale unified model of human mobility in urban agglomerations

Yong Chen, Haoge Xu, Xiqun (Michael) Chen, and Ziyou Gao

Supplemental Information

Supplemental Items

Supplemental Notes

Note S1. Nested logit (NL) model

The NL model [1] represents individual travel choice behavior in a nested form (see Figure S2), and the probability $P^i(c)$ of selecting alternative c at the first layer can be represented as:

$$P^i(c) = \frac{e^{\theta_1 V_c^i}}{\sum_{j\in\mathbb{C}} e^{\theta_1 V_j^i}} \tag{1}$$

$$V_c^i = \sum_{d=1}^{D} \beta_d^1 x_{dc}^i \tag{2}$$

where V_c^i represents the utility function of individual i selecting alternative c, which is calculated from D attributes x_{dc}^i (e.g., time, and cost). $\mathbb{C}$ is the set of alternatives at the first layer. θ_1 is the scale parameter of the first layer. β_d^1 is the preference parameter of each attribute at the first layer. Based on the current selection, the probability $P^i(cq)$ of selecting alternative q in nest c can be represented as follows:

$$P^i(cq) = P^i(q|c)P^i(c) \tag{3}$$

$$P^i(q|c) = \frac{e^{\theta_2 V_{cq}^i}}{\sum_{j\in\mathbb{Q}} e^{\theta_2 V_{cj}^i}} \tag{4}$$

$$V_{cq}^i = \sum_{d=1}^{D} \beta_d^2 x_{dcq}^i \tag{5}$$

where the conditional selection probability $P^i(q|c)$ is calculated by the utility function of alternatives. $\mathbb{Q}$ is the set of alternatives in nest c. θ_2 is the scale parameter of the second layer. β_d^2 is the preference parameter of attribute x_{dcq}^i in nest c. To strengthen the relationship between the upper and lower layers in the NL model, the utility expectation values of all the alternatives of the lower layer are used as an attribute of its upper layer model to realize the feedback of the lower layer to the upper layer, and Eq. (2) is modified as follows:

$$V_c^i = \sum_{d=1}^{D} \beta_d^1 x_{dc}^i + \frac{1}{\theta_c} \cdot \ln\left(\sum\nolimits_{j\in\mathbb{Q}} \theta_c \, e^{V_{cj}^i}\right) \tag{6}$$

where θ_c is the corresponding model parameter.

Note S2. Convolutional neural network (CNN)

Deep CNN without bias parameter is used as a classifier to automatically extract human travel patterns, and correctly output travel choice probabilities at different spatial scales based on individual memory and population attractiveness feature inputs. At each scale, deep CNN is constructed by four one-dimensional convolution layers, and the convolution calculation of each layer is shown below:

$$\mathbf{X}_d^{l+1} = relu(\sum_{d=1}^{D} \mathbf{X}_d^l \, \boldsymbol{\beta}_d^l) \tag{7}$$

where $\mathbf{X}_d^l$ denotes the d-th dimension feature inputs of the l-th convolutional layer, and the first layer includes original individual memory and population attractiveness features. D denotes the dimension of the feature vector. $\boldsymbol{\beta}$ denotes the parameters of the convolution filter. $relu(\cdot)$ denotes the rectified linear unit function [2]. CNN at different scales have similar model frameworks, and none use bias parameters. At the county scale, the number of filters in each layer of CNN is 16, 16, 16, and 1, respectively. At the community scale, the number of filters in each layer of CNN is 16, 32, 32, and 1, respectively. In addition, the last layer of CNN multiplies the model output of CNN at the county scale to implement conditional probability constraints. At the same time, the classification error calculated in the community scale back-propagates to the upper layer (i.e., CNN at the county scale) to update network parameters.

Note S3. Graph generative adversarial network

We model the prediction of travel probability between different locations as a process of missing data imputation, and propose graph generative adversarial networks (GGAN) to impute data. A generative adversarial network (GAN) is a deep learning framework consisting of two neural networks (i.e., generator, and discriminator) pitted against each other. It has been widely used to solve tasks such as image restoration and traffic prediction [3]. As shown in Figure S1, GGAN consists of generator G and discriminator D. Considering that the transition between locations can be expressed as a travel network, it has a graph structure with non-Euclidean characteristics. Therefore, we employ a graph convolutional network as generator G. Assumed that the trip distribution matrix between C locations is represented as $\mathbf{X} \in \mathbb{R}^{C \times C}$, and its component x_{ij} represents the travel probability between two locations, calculated by the travel volume between locations. Note that the travel volume between some locations is unknown; thus, the travel probability cannot be calculated, which we treat as missing data. The missing state of the travel probability between locations is represented by a matrix $\mathbf{Ms} \in \{0,1\}^{C \times C}$. We use generator G to automatically learn the mapping relationship between travel probability and distance, population difference between two locations, and then impute the travel probability between those locations with unknown travel volume. The imputation process is calculated as follows:

$$\mathbf{h}_d = LeakyRelu(\mathbf{D} \odot \mathbf{X} \cdot \mathbf{W}_d) \tag{8}$$

$$\mathbf{h}_p = LeakyRelu(\mathbf{PD} \odot \mathbf{X} \cdot \mathbf{W}_p) \tag{9}$$

$$\widetilde{\mathbf{X}} = LeakyRelu([\mathbf{h}_d, \mathbf{h}_p] \cdot \mathbf{W}_{dp}) \tag{10}$$

where $\mathbf{D}$ denotes the spatial distance matrix. $\mathbf{PD}$ denotes the population difference matrix. Referring to previous empirical studies [4], we use the distance between locations, and the population difference between locations as the feature matrix. The component in $\mathbf{PD}$ is calculated by $1 + \frac{\log(n_2) - \log(n_1)}{\log(n_1)}$, wherein n denotes population size. $\mathbf{h}_d$ and $\mathbf{h}_p$ denote a distance map and a population map obtained after feature extraction by graph convolution, respectively. $[\cdot]$ denotes concatenation operation. $\mathbf{W}_{dp}$, $\mathbf{W}_d$, and $\mathbf{W}_p$ denote the corresponding parameter matrix. $\odot$ denotes the Hadamard product [5] between two matrices. $LeakyRelu(\cdot)$ denotes the leaky rectified linear unit activation function. $\widetilde{\mathbf{X}}$

denotes the imputed trip distribution matrix obtained by the convolutional operation after concatenating the distance map and the feature map.

On the other hand, discriminator D is used to discriminate which components are observable and which are missing in $\widetilde{\mathbf{X}}$. The inputs of the discriminator include matrix $\widetilde{\mathbf{X}}$ and reminder matrix $\mathbf{Mr}$. Inspired by the research of Yoon et al. [6], $\mathbf{Mr} = \mathbf{Ms} \odot \mathbf{B} + 0.5(1 - \mathbf{B})$ is used to improve the convergence of the discriminator, wherein $\mathbf{B} \in \{0,1\}^{C\times C}$ denotes a random matrix. In $\mathbf{Mr}$, the element values include 0, 0.5, and 1. 0 and 1 indicate to remind the discriminator whether the corresponding element is missing. 0.5 denotes a neutral state, which means that no reminder is given to the discriminator. We use a three-layer fully connected network to represent the discriminator, and the discrimination process is calculated as follows:

$$\mathbf{h}_{dis}^{l+1} = LeakyRelu([\widetilde{\mathbf{X}}, \mathbf{Mr}] \cdot \mathbf{W}_{dis}^{l}) \tag{11}$$

$$\mathbf{Ms} = \sigma(\mathbf{h}_{dis}^{l+1}\mathbf{W}_{dis}^{l+1}) \tag{12}$$

where $\mathbf{h}_{dis}^{l+1}$ denotes the output of the l-th layer of the discrimination network, which serves as a feature input of the next layer. $\mathbf{Ms}$ is a discriminant matrix, whose components take values from zero to one, indicating the missing possibility of the corresponding travel volume. $\mathbf{W}_{dis}^{l}$ and $\mathbf{W}_{dis}^{l+1}$ represent the corresponding parameter matrix. $\sigma(\cdot)$ denotes the sigmoid function.

The generator and discriminator are trained adversarially through a minimax game [7], and their loss functions are defined as follows:

$$\mathcal{L}_D = -\mathbb{E}[(1 - \mathbf{B}) \odot \mathbf{Ms} \odot \log(\mathbf{Ms}) + (1 - \mathbf{B}) \odot (1 - \mathbf{Ms}) \odot \log(1 - \mathbf{Ms})] \tag{13}$$

$$\mathcal{L}_G = \mathcal{L}_{G1} + \beta \cdot \mathcal{L}_{G2} = -\mathbb{E}[(1 - \mathbf{B}) \odot (1 - \mathbf{Ms}) \odot \log(\mathbf{Ms})] + \beta \cdot RMSE(\mathbf{X} \odot \mathbf{Ms}, \widetilde{\mathbf{X}} \odot \mathbf{Ms}) \tag{14}$$

where $\mathcal{L}_D$ denotes the probability of correctly predicting the missing matrix $\mathbf{Ms}$, and the discriminator is trained by minimizing $\mathcal{L}_D$. The generator is trained by minimizing $\mathcal{L}_G$, which consists of the discriminant error and the reconstruction error. β denotes the weight coefficient. The generator aims to make the observable components of $\widetilde{\mathbf{X}}$ is consistent with the actual data, and the missing components discriminator is challenging to distinguish the true from the false.

Supplemental Tables

Table S1. Prediction performance comparison of different models at county and community scales in Boswash urban agglomeration

Name	Abb.	GM			RM			PWO			GGAN		
		CPC	RMSE	MAE	CPC	RMSE	MAE	CPC	RMSE	MAE	CPC	RMSE	MAE
ALL	ALL	0.446	**65.567**	10.875	0.470	164.506	9.965	0.406	191.785	17.470	**0.490**	82.201	**8.906**
Ave.	Ave.	0.197	5.236	0.787	0.379	4.902	0.545	0.459	4.206	0.592	**0.503**	**3.498**	**0.475**
New York County	NY	0.584	214.197	38.216	0.509	237.877	32.197	0.666	166.731	32.351	**0.743**	**164.708**	**25.150**
District of Columbia	DC	0.448	53.163	6.927	0.531	50.169	4.173	0.656	41.852	3.702	**0.806**	**18.889**	**2.394**
Suffolk County (Massachusetts)	SCM	0.289	48.819	4.911	0.455	35.250	2.462	0.514	30.907	2.483	**0.723**	**16.787**	**1.825**
Kings County	KC	0.368	40.489	6.025	0.596	29.489	**2.934**	0.647	29.432	3.115	**0.648**	**27.695**	3.308
Philadelphia County	PC	0.273	23.110	1.882	0.317	20.768	**1.140**	0.420	19.065	1.256	**0.481**	**16.014**	1.468
Baltimore County	BC	0.271	1.785	0.255	0.486	1.295	0.144	0.612	**1.175**	0.160	**0.668**	1.289	**0.132**
Middlesex County	MC	0.351	14.393	1.946	0.527	9.330	1.043	0.405	12.761	1.867	**0.660**	**6.671**	**0.998**
Queens County	QC	0.325	8.322	1.315	0.452	**7.465**	**0.758**	**0.545**	7.742	0.904	0.431	7.813	0.991
Nassau County	NC	0.404	4.623	0.768	0.522	**3.176**	**0.453**	**0.598**	4.497	0.698	0.567	3.599	0.625
Suffolk County (New York)	SCN	0.308	4.186	0.897	0.541	3.180	**0.442**	0.604	4.385	0.805	**0.607**	**3.134**	0.536

Note: "Abb." stands for abbreviation. Bold font represents the optimal value of each measure of effectiveness.

Table S2. Prediction performance comparison of different models at county and community scales in Great Lakes urban agglomeration

Name	Abb.	GM			RM			PWO			GGAN		
		CPC	RMSE	MAE	CPC	RMSE	MAE	CPC	RMSE	MAE	CPC	RMSE	MAE
ALL	ALL	0.135	17.412	1.304	0.437	14.293	**0.623**	0.272	16.581	0.909	**0.505**	**12.801**	0.771
Ave.	Ave.	0.134	2.174	0.243	0.329	1.737	0.128	0.358	1.888	0.163	**0.522**	**1.257**	**0.117**
Dane County	DC	0.272	5.772	0.787	0.551	3.584	0.348	0.645	3.190	0.391	**0.703**	**2.897**	**0.322**
Milwaukee County	MIC	0.201	13.000	1.207	0.559	8.736	0.468	0.288	12.272	0.702	**0.768**	**5.607**	**0.329**
Cook County	COC	0.351	31.144	3.607	0.586	20.250	1.698	0.534	27.850	2.785	**0.713**	**15.993**	**1.402**
Oakland County	OC	0.395	8.261	1.395	0.512	7.179	0.823	0.609	8.140	1.166	**0.639**	**4.863**	**0.822**
Marion County	MC	0.547	1.935	0.429	0.545	1.924	**0.323**	0.452	5.002	0.773	**0.662**	**1.478**	0.327
Franklin County	FC	0.378	24.525	2.261	0.431	21.105	1.376	0.476	19.872	1.600	**0.608**	**17.193**	**1.356**
Cuyahoga County	CC	0.423	4.367	0.668	0.460	4.281	0.467	**0.685**	**3.392**	**0.383**	0.659	4.158	0.437
Allegheny County	AC	0.407	8.212	1.199	0.389	7.787	0.796	0.613	**5.207**	0.859	**0.629**	5.617	**0.746**
Erie County	EC	0.287	8.173	1.108	0.397	7.758	0.622	0.533	6.297	0.676	**0.622**	**4.571**	**0.543**
Monroe County	MOC	0.392	12.362	1.807	0.563	10.200	0.924	0.619	8.850	1.061	**0.707**	**7.893**	**0.867**

Table S3. Prediction performance comparison of different models at county and community scales in England urban agglomeration

Names	Abb.	GM			RM			PWO			GGAN		
		CPC	RMSE	MAE	CPC	RMSE	MAE	CPC	RMSE	MAE	CPC	RMSE	MAE
ALL	ALL	0.485	**15.592**	4.861	0.387	33.848	4.772	0.487	25.451	5.072	**0.571**	17.537	**4.420**
Ave.	Ave.	0.071	0.403	0.041	0.265	0.322	**0.026**	0.373	0.364	0.039	**0.381**	**0.279**	0.028
Brighton and Hove County	BH	0.233	2.275	0.286	0.288	2.182	0.173	0.303	2.340	0.238	**0.644**	**1.728**	**0.132**
Camden County	CD	0.157	0.620	0.099	**0.474**	**0.423**	**0.062**	0.459	0.899	0.133	0.426	0.522	0.078
Hertfordshire County	HS	0.156	1.228	0.119	0.364	1.131	0.065	0.419	1.292	0.112	**0.617**	**0.601**	**0.057**
Lancashire County	LS	0.077	0.756	0.095	0.416	0.522	**0.052**	0.496	0.532	0.078	**0.557**	**0.485**	0.058
Manchester County	MC	0.233	2.462	0.387	0.516	1.740	0.195	0.501	2.141	0.289	**0.596**	**1.464**	**0.190**
South Yorkshire County	SY	0.152	0.748	0.099	0.333	0.675	0.058	0.421	0.707	0.079	**0.432**	**0.574**	**0.057**
Tyne and Wear County	TW	0.215	0.714	0.081	0.219	0.677	0.063	0.276	0.901	0.113	**0.515**	**0.466**	**0.057**
West Midlands County	WMS	0.154	1.320	0.199	0.316	1.119	0.128	0.455	0.976	0.172	**0.518**	**0.953**	**0.117**
West Yorkshire County	WY	0.099	1.904	0.160	0.333	1.369	0.099	0.478	1.132	0.128	**0.516**	**0.758**	**0.095**
Westminster County	WM	0.341	1.456	0.313	0.444	1.050	**0.217**	**0.535**	1.923	0.367	0.519	**1.009**	0.252

Table S4. CPC values of different models at different active regions

Active regions	Boswash agglomeration				Great Lakes agglomeration				England agglomeration			
	GM	RM	PWO	GGAN	GM	RM	PWO	GGAN	GM	RM	PWO	GGAN
1	0.373	0.501	0.564	**0.634**	0.364	0.497	0.545	**0.665**	0.182	0.370	0.434	**0.534**
2	0.287	0.419	**0.533**	0.526	0.269	0.484	0.511	**0.637**	0.140	0.393	0.460	**0.484**
3	0.259	0.431	**0.512**	0.504	0.169	0.397	0.314	**0.673**	0.120	0.307	**0.402**	0.363
4	0.205	0.435	0.531	**0.548**	0.154	0.390	0.404	**0.515**	0.083	0.349	0.407	**0.430**
5	0.190	0.355	0.477	**0.486**	0.090	0.298	0.404	**0.494**	0.051	0.286	0.409	**0.421**
6	0.183	0.327	0.393	**0.453**	0.100	0.256	0.176	**0.464**	0.071	0.312	0.411	**0.494**
7	0.181	0.394	0.471	**0.479**	0.076	0.358	0.333	**0.540**	0.014	0.166	**0.358**	0.304
8	0.128	0.382	0.410	**0.519**	0.069	0.224	0.266	**0.417**	0.037	0.258	**0.355**	0.328
9	0.103	0.295	0.360	**0.475**	0.000	0.163	0.285	**0.423**	0.000	0.139	**0.265**	0.220
10	0.041	0.237	0.321	**0.400**	0.000	0.168	**0.335**	0.308	0.000	0.021	0.196	**0.197**

Table S5. Performance comparison of multi-scale human mobility prediction in different agglomerations

Urban Agglomeration	Measure of effectiveness	EPR	D-EPR	R-EPR	M-EPR	UMIP	MSUM
Boswash	CPC	0.170	0.267	0.263	0.378	0.310	**0.672**
	RMSE	29.066	28.665	25.043	23.839	24.956	**13.220**
	MAE	4.285	4.150	4.141	3.706	4.903	**2.791**
Great Lakes	CPC	0.064	0.192	0.137	0.222	0.444	**0.542**
	RMSE	19.396	18.877	18.629	17.447	22.385	**14.387**
	MAE	3.918	3.679	3.782	3.594	3.601	**3.324**
England	CPC	0.085	0.128	0.125	0.175	0.221	**0.319**
	RMSE	2.991	2.956	2.863	2.516	3.647	**2.072**
	MAE	1.376	1.348	1.369	1.321	1.497	**1.268**

Table S6. Performance comparison of human mobility prediction at the county scale in different agglomerations

Urban Agglomeration	Measure of effectiveness	EPR	D-EPR	R-EPR	M-EPR	UMIP	MSUM
Boswash	CPC	0.383	0.432	0.535	0.592	0.415	**0.877**
	RMSE	533.318	510.448	392.632	337.654	506.146	**51.634**
	MAE	37.670	38.070	31.640	28.423	42.217	**9.322**
Great Lakes	CPC	0.228	0.412	0.360	0.397	0.669	**0.816**
	RMSE	123.701	109.841	111.531	106.540	55.371	**36.702**
	MAE	18.708	17.417	17.190	16.393	12.032	**7.235**
England	CPC	0.556	0.543	0.564	0.623	0.499	**0.736**
	RMSE	13.462	14.029	11.868	10.481	16.078	**4.448**
	MAE	3.238	3.541	3.412	2.906	4.303	**2.188**

Supplemental Figures

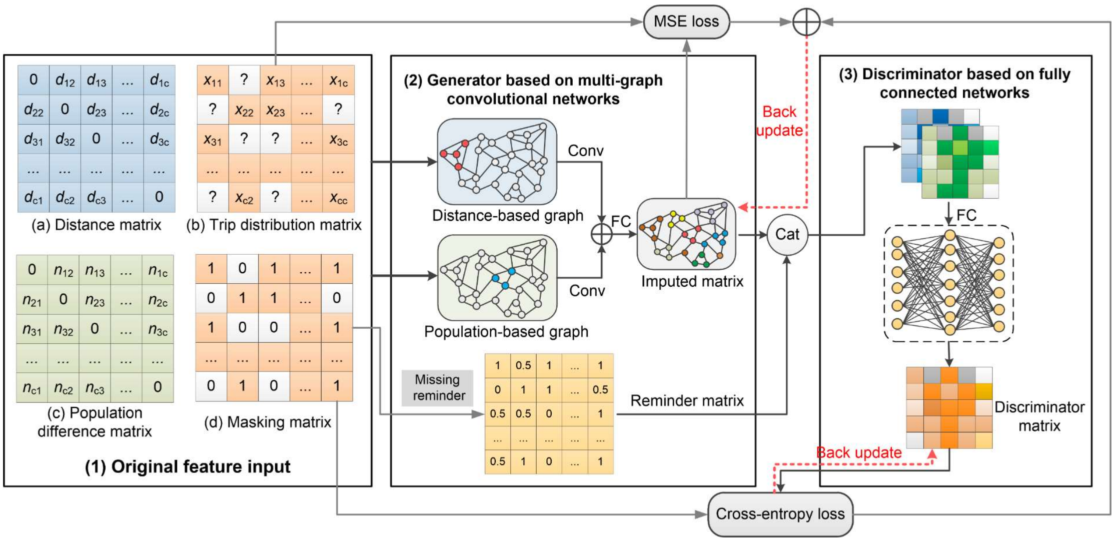

Figure S1. Architecture of graph generative adversarial networks model. The model consists of three modules: original feature input, generator, and discriminator. The feature inputs include a trip distribution matrix between locations, a masking matrix representing the missing data state, and distance and population difference matrices used to construct the virtual graph. The implementation of the generator includes two steps. First, based on the distance matrix and population difference matrix, the graph convolution operations (i.e., "Conv") based on the distance and population graphs are performed, respectively. Second, the feature matrices are concatenated after feature extraction, input into a fully connected network (i.e., "FC") for mixed feature extraction, and an imputed trip distribution matrix is output. The discriminator is represented by a fully connected network whose feature inputs include an imputed missing matrix and a missing reminder matrix, where the missing reminder matrix is used to accelerate the discriminator's convergence. "Cat" denotes the concatenate operation. The loss function of the generator consists of the discriminant error (i.e., cross-entropy loss) and reconstruction error (i.e., mean squared error loss). The loss function of the discriminator is the cross-entropy loss.

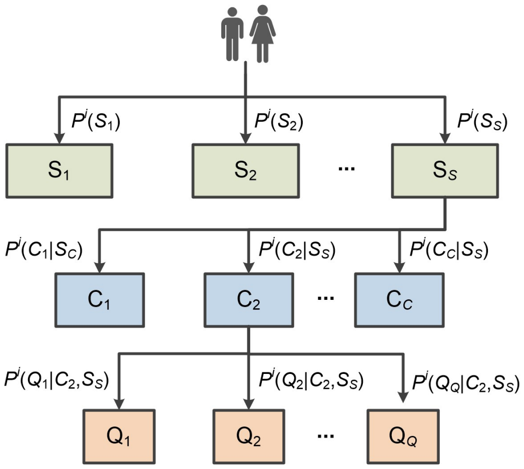

Figure S2. Schematic representation of the nested logit model. Individuals hierarchically select alternatives in a nested structure (see Note S2), and the selection process is constrained by conditional probability. At the first layer, the individual selects alternative S_S with probability $P^i(S_S)$. Then, in nest S_S, alternative C_C is selected with probability $P^i(C_C|S_S)$. Further, in the selected nest C_C, alternative Q_Q is selected with probability $P^i(Q_Q|C_C,S_S)$.

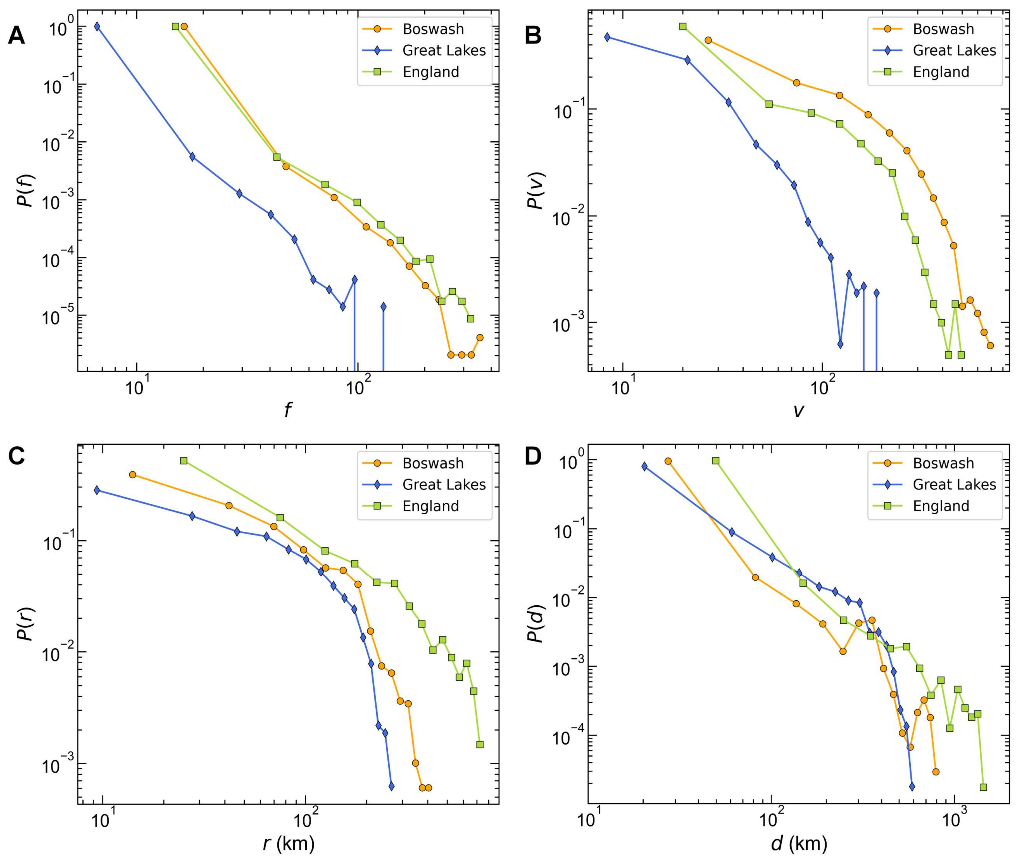

Figure S3. Empirical statistics of human mobility patterns in three urban agglomerations. (**A**) Frequency distribution of individuals visiting a location. (**B**) Probability distribution of v different locations visited in total. (**C**) Radius of gyration distribution. (**D**) Travel distance distribution at multiple spatial scales. All distributions are calculated from actual individual travel traces, and individuals move at multiple spatial scales within urban agglomerations, including intra- and inter-county.

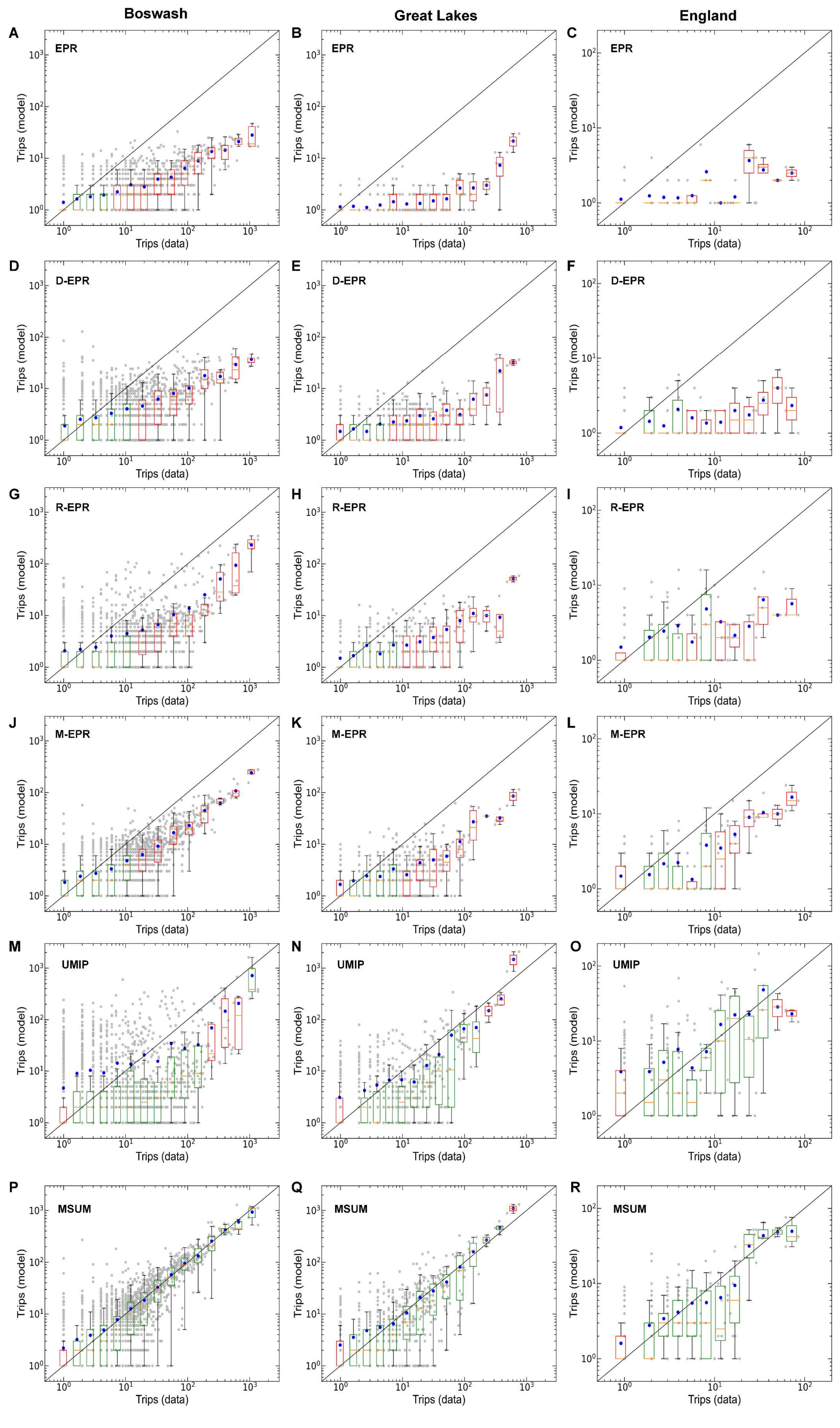

Figure S4. Paired comparisons of predicted and real trips. Gray points indicate observed and predicted location pairs. Blue points indicate the average number of predicted trips in different bins. The boxplot indicates the distribution of the number of predicted trips in different bins of the number of observed trips. A box is green if the black line $y = x$ is between the 5th and 95th percentiles of the box, and red otherwise. To characterize the model's prediction performance, all distributions are calculated by aggregating simulated and actual individual traces.

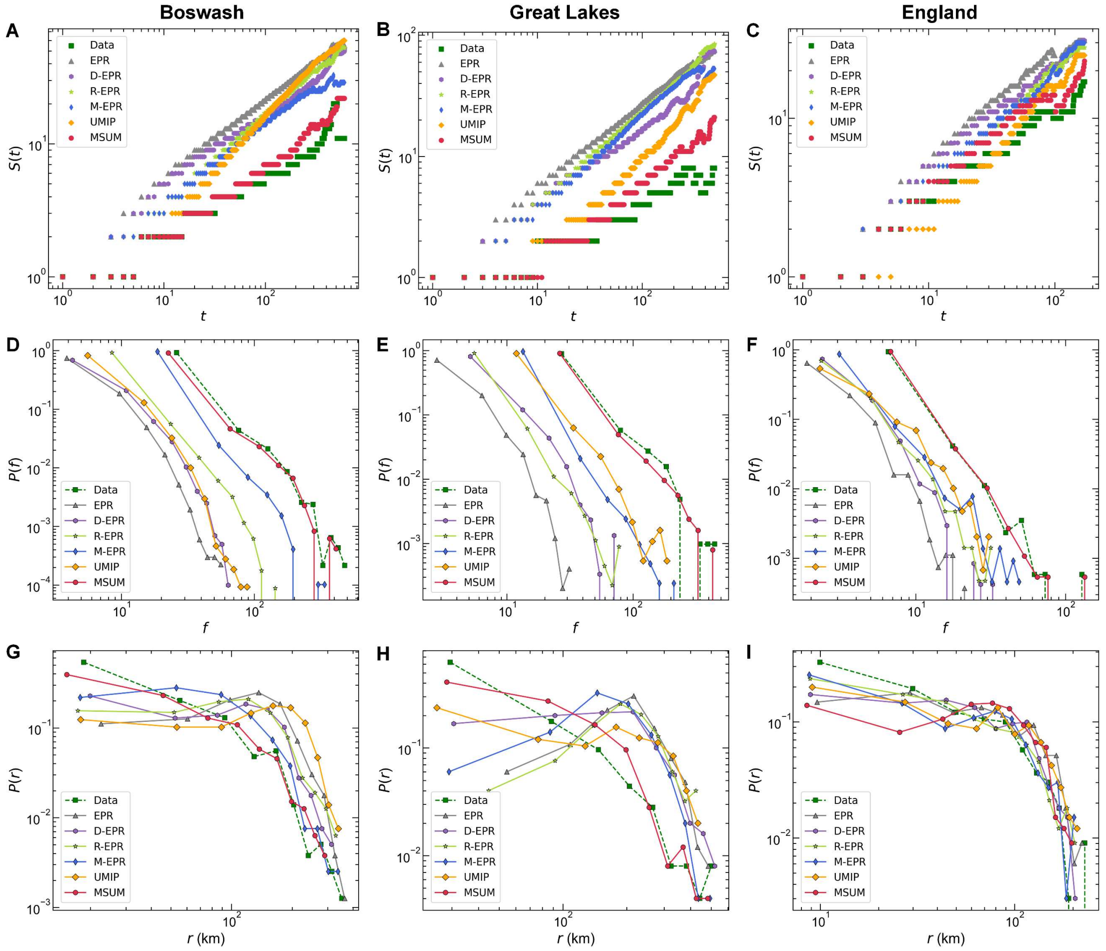

Figure S5. Single-scale human mobility prediction in urban agglomeration at the individual level. (**A–C**) Distribution of the total number of counties visited in t trips. (**D–F**) Frequency distribution of individuals visiting a county. (**G–I**) Radius of gyration distribution. All distributions are calculated from the simulated and the actual individual traces. The calculated distributions focus on the individuals' travel characteristics between counties (i.e., single spatial scale).

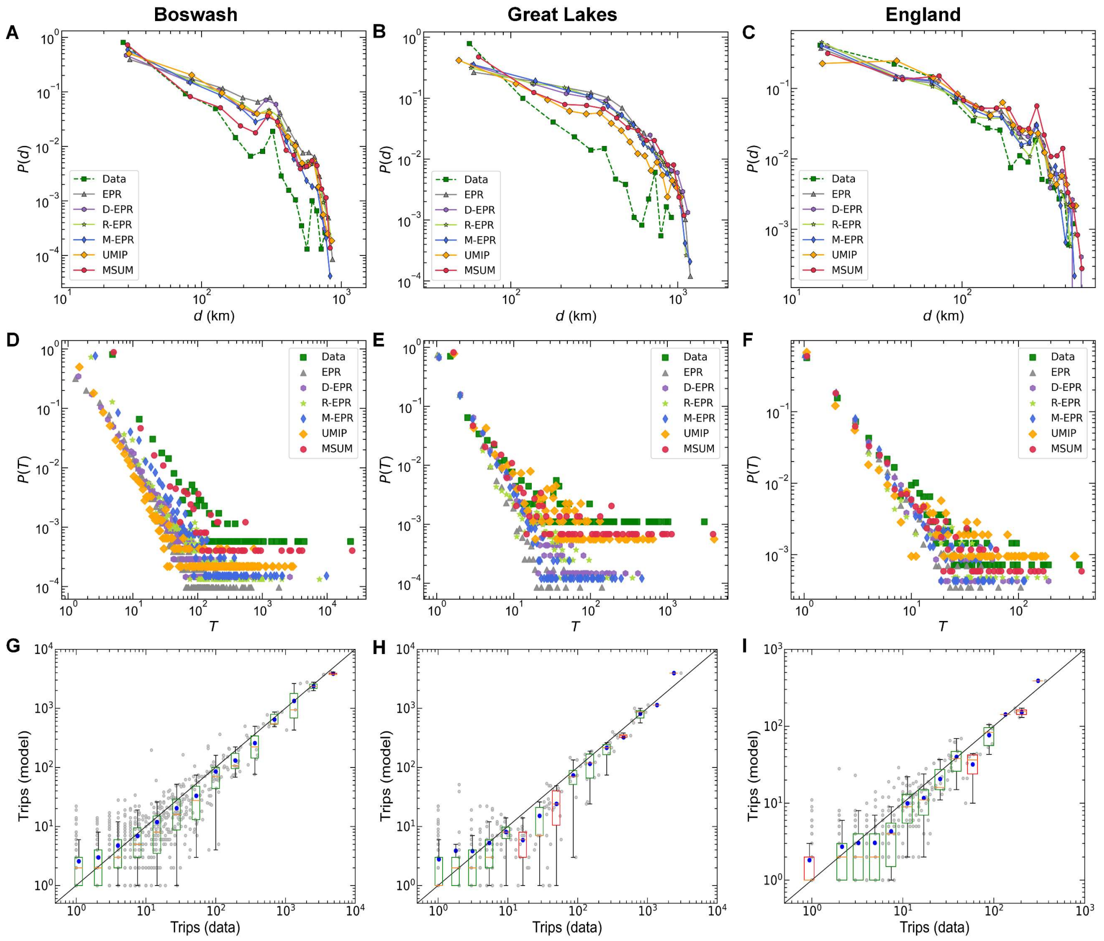

Figure S6. Single-scale human mobility prediction in urban agglomeration at the population level. (**A–C**) Predicted and real distributions of travel distance. (**D–F**) Predicted and real distributions of the number of trips between two counties. (**G–I**) Paired comparisons of predicted and real trips. Gray points indicate observed and predicted county pairs. Blue points indicate the average number of predicted trips in different bins. The boxplot indicates the distribution of the number of predicted trips in different bins of the number of observed trips. A box is green if the black line $y = x$ is between the 5th and 95th percentiles of the box, and red otherwise. To characterize the model's single-scale human mobility (i.e., inter-county travel) prediction performance at the population level, all distributions are calculated by aggregating simulated and actual individual traces, respectively.

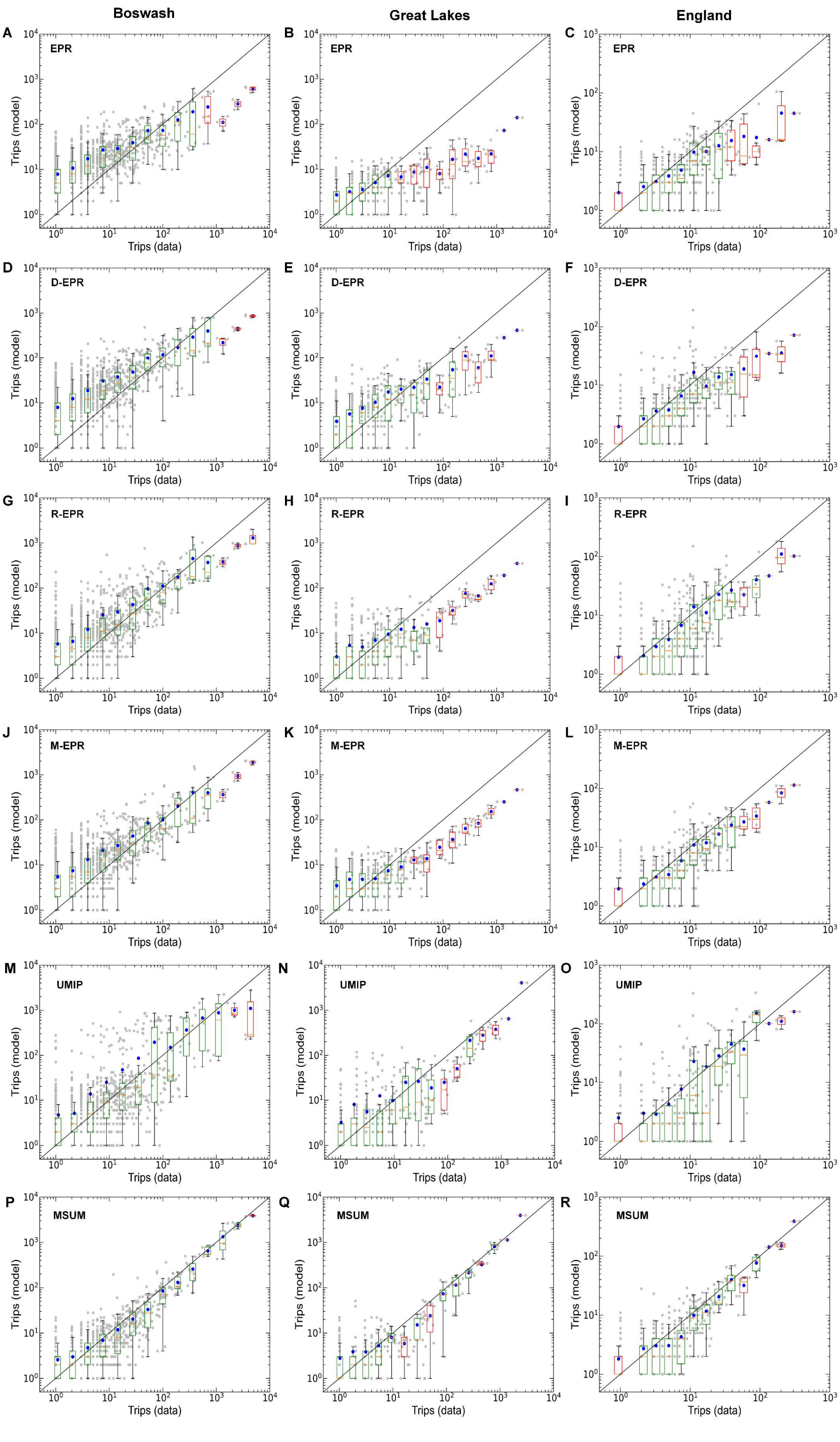

Figure S7. Paired comparisons of predicted and real trips at the county scale. Gray points indicate observed and predicted county pairs. Blue points indicate the average number of predicted trips in different bins. The boxplot indicates the distribution of the number of predicted trips in different bins of the number of observed trips. A box is green if the black line $y = x$ is between the 5th and 95th percentiles of the box, and red otherwise. To characterize the model's human mobility prediction performance at a single spatial scale, all distributions are calculated by aggregating simulated and actual individual traces, respectively.

Supplemental Experimental Procedures

Baseline models

To verify the accuracy and robustness of graph generative adversarial network (GGAN), three widely used models are selected as the baseline for performance comparison, including the gravity model (GM) [8], radiation model (RM) [9], and population-weighted opportunities model (PWO) [10].

GM model assumes that the travel volume between two locations is proportional to their population and decays as the power of the distance between them. We use the common origin-constrained form of GM, which is defined as follows:

$$T_{ij}^{GM} = T_{i,:} \cdot \frac{Pop_j^{\gamma_1} d_{ij}^{-\gamma_2}}{\sum_k Pop_k^{\gamma_1} d_{ik}^{-\gamma_2}} \tag{15}$$

where $T_{i,:}$ denotes the total travel flows from location i. Pop_j denotes the population of location j. d_{ij} denotes the distance between location i and location j. γ_1 and γ_2 are parameters to be estimated.

RM model is inspired by the radiation and absorption of particles to make trip distribution predictions based on the population between locations without parameter estimation, and is defined as follows:

$$T_{ij}^{RM} = T_{i,:} \cdot \frac{Pop_i Pop_j}{(Pop_i + s_{ij})(Pop_i + s_{ij} + Pop_j)} \tag{16}$$

where s_{ij} denotes the number of intervention opportunities (i.e., the total population) between location i and location j.

PWO model assumes that the attractiveness of the destination declines with distance, and is inversely proportional to the population S_{ji} in the circle centered at the destination with radius d_{ij}, minus a finite-size correction term:

$$T_{ij}^{PWO} = T_{i,:} \cdot \frac{Pop_j(1/S_{ji} - 1/P_{total})}{\sum_{k \neq i} Pop_k(1/S_{ki} - 1/P_{total})} \tag{17}$$

$$S_{ji} = Pop_i + s_{ji} + Pop_j \tag{18}$$

where P_{total} is the total population of all locations.

Moreover, to verify the prediction performance of the multi-scale unified model (MSUM) at multiple spatial scales in urban agglomerations, the exploration and preferential return [11] (EPR) model, three EPR variant models, i.e., gravity EPR [12] (D-EPR), recency EPR [13] (R-EPR), and memory EPR [14] (M-EPR), and universal model of individual and population (UMIP) [15] are used for performance comparison.

In EPR model, for each new movement, an individual explores a new location with probability $\rho S^{-\gamma}$, or chooses to return to a previously visited location with probability $1 - \rho S^{-\gamma}$, where S represents the total number of locations that the individual has visited. ρ and γ are the model parameters, and 0.6 and 0.21 are adopted in this paper, respectively, which is based on the empirical observations in previous study [4,11]. In the return phase, the probability of an individual visiting a location is proportional to the frequency of his/her previous visits. In the exploration phase, the individual chooses a location to travel according to the distance distribution among the locations.

D-EPR model optimizes the exploration phase of the EPR model. When exploring a new location, the individual chooses a new location to travel with selection probability $p_{ij} = \frac{1}{SP}\frac{Pop_i Pop_j}{d_{ij}^2}$ based on the gravity model, where $SP = \sum_{i,j\neq i} p_{ij}$ is a normalization factor.

R-EPR model optimizes the return phase of the EPR model by considering recency influence. R-EPR model represents the return probability of an individual through ranking, that is, people usually consider recently-visited locations to travel. In the return phase, the individual chooses to rank candidate locations based on the *frequency* with probability α or the *recency* with probability $1-\alpha$. In the first case, the individual selects a return destination with probability $Rank_f(l_i)^{-1-\gamma}$ based on the historical visit frequency, where $Rank_f(l_i)$ represents the frequency-based ranking of location l_i. In the latter case, the individual will choose the i-th last visited location to return to with probability $Rank_r(l_i)^{-\eta}$ selecting from a Zipf distribution, where $Rank_r(l_i)$ represents the recency-based ranking of location l_i. Parameters α, γ, and η are set to 0.1, 0.21, and 1.6, respectively, based on the empirical observations in the previous study [13].

M-EPR model optimizes the return phase of the EPR model by adding a memory limit to EPR. In the M-EPR model, only the historical memory of previous M days affects the individual's travel choice behavior in the return phase. In our study, we set M to be 92.17% of the total movement steps, which approximates the time ratio in the original study [14].

UMIP model combines individual memory effect and population-induced competition to achieve the unified individual and population mobility prediction.

$$p_{ij} \propto \frac{Pop_j}{S_{ji}}\left(1+\frac{\lambda}{r_j}\right) \tag{19}$$

where p_{ij} denotes the transition probability from location i to j. The individual memory effect (i.e., $1+\frac{\lambda}{r_j}$) is quantified by the rank of visits to different locations. r_j denotes that location j is the r-th newly visited location of the current individual. λ denotes the model parameter used to characterize the strength of the memory effect, which is estimated from empirical data and takes values of 30 (Boswash), 30 (Great Lakes), and 60 (England), respectively. The population-induced competition (i.e., $\frac{Pop_j}{S_{ji}}$) is quantified by the population between the origin and the destination, and the specific calculation method is the same as that of PWO.

Measures of Effectiveness

Three widely used measures of effectiveness are used to quantify the model prediction performance, i.e., common part of commuters [4] (CPC), mean absolute error (MAE), and root mean squared error (RMSE), which are defined as follows:

$$\mathrm{CPC} = \sum_{i}^{N}\sum_{j\neq i}^{N}\frac{2\times\min(T_{ij}, T'_{ij})}{T_{ij}+T'_{ij}} \tag{20}$$

$$\mathrm{MAE} = \frac{1}{N(N-1)}\sum_{i}^{N}\sum_{j\neq i}^{N}|T_{ij}-T'_{ij}| \tag{21}$$

$$\mathrm{RMSE} = \sqrt{\frac{1}{N(N-1)}\sum_{i}^{N}\sum_{j\neq i}^{N}\left(T_{ij}-T'_{ij}\right)^2} \tag{22}$$

where T_{ij} and T'_{ij} denote observed and predicted travel flows, from location i to location j, respectively. N denotes the total number of locations.

Performance comparison of single-scale mobility prediction between MSUM and baseline models

By aggregating all individual simulation traces at the county level, we can compare the mobility prediction performance of MSUM with that of baseline models (i.e., EPR, D-EPR, R-EPR, and M-EPR) at a single spatial scale. Figures S5-S7 present the model comparison results at individual and population levels. At the same time, Table S6 quantifies the prediction performance of each model using CPC, RMSE, and MAE. At the individual level, MSUM can describe individual mobility patterns consistent with empirical distributions, whereas the baseline models have poor prediction accuracy in individual mobility patterns. At the population level, MSUM can accurately predict the inter-county travel flow, and has a stable prediction performance under different travel flow intensities. Meanwhile, as shown in Figure S7, the baseline models underestimate travel flow, especially in the range of 10^2 through 10^3. Therefore, the proposed MSUM can achieve superior performance in the single-scale human mobility prediction task.

Supplemental References

1. Williams, H.C. (1977). On the formation of travel demand models and economic evaluation measures of user benefit. *Environ. Plan. A. 9*, 285–344. https://doi.org/10.1068/a090285
2. Nair, V., and Hinton, G.E. (2010). Rectified linear units improve restricted Boltzmann machines. In *Proc. 27th International Conference on Machine Learning*, 807–814. https://dl.acm.org/doi/10.5555/3104322.3104425
3. Creswell, A., White, T., Dumoulin, V., Arulkumaran, K., Sengupta, B., and Bharath, A.A. (2018). Generative adversarial networks: An overview. *IEEE Signal Process. Mag. 35*, 53–65. https://doi.org/10.1109/MSP.2017.2765202
4. Barbosa, H. et al. (2018). Human mobility: Models and applications. *Phys. Rep. 734*, 1–74. https://doi.org/10.1016/j.physrep.2018.01.001
5. Davis, C. (1962). The norm of the Schur product operation. *Numer. Math. 4*, 343–344. https://doi.org/10.1007/BF01386329
6. Yoon, J., Jordon, J., and Schaar, M. (2018). Gain: Missing data imputation using generative adversarial nets. In *Proc. International Conference on Machine Learning*, pp. 5689–5698. http://proceedings.mlr.press/v80/yoon18a.html
7. Goodfellow, I. et al. (2014). Generative adversarial nets. In *Proc. 28th Conference on Neural Information Processing Systems 2*, 2672–2680. https://dl.acm.org/doi/10.5555/2969033.2969125
8. Zipf, G.K. (1946). The P_1 P_2/D hypothesis: On the intercity movement of persons. *Am. Sociol. Rev. 11*, 677–686. https://doi.org/10.2307/2087063
9. Simini, F., González, M.C., Maritan, A., and Barabási, A.L. (2012). A universal model for mobility and migration patterns. *Nature 484*, 96–100. https://doi.org/10.1038/nature10856
10. Yan, X.Y., Zhao, C., Fan, Y., Di, Z.R., and Wang, W.X. (2014). Universal predictability of mobility patterns in cities. *J. R. Soc. Interface 11*, 20140834. https://doi.org/10.1098/rsif.2014.0834
11. Song, C., Koren, T., Wang, P., and Barabási, A.L. (2010). Modelling the scaling properties of human mobility. *Nat. Phys. 6*, 818–823. https://doi.org/10.1038/nphys1760
12. Pappalardo, L. et al. (2015). Returners and explorers dichotomy in human mobility. *Nat. Commun. 6*, 8166. https://doi.org/10.1038/ncomms9166
13. Barbosa, H., de Lima-Neto, F.B., Evsukoff, A., and Menezes, R. (2015). The effect of recency to human mobility. *EPJ Data Sci. 4*, 21. http://dx.doi.org/10.1140/epjds/s13688-015-0059-8
14. Alessandretti, L., Sapiezynski, P., Sekara, V., Lehmann, S., and Baronchelli, A. (2018). Evidence for a conserved quantity in human mobility. *Nat. Hum. Behav. 2*, 485–491. https://doi.org/10.1038/s41562-018-0364-x
15. Yan, X.Y., Wang, W.X., Gao, Z.Y., and Lai, Y.C. (2017). Universal model of individual and population mobility on diverse spatial scales. *Nat. Commun. 8*, 1639. https://doi.org/10.1038/s41467-017-01892-8
