# Supplementary material for: A multi-scale unified model of human mobility in urban agglomerations
Source: Patterns (N Y). 2023 Oct 17;4(11):100862. doi: 10.1016/j.patter.2023.100862 (PMC10682749; doi:10.1016/j.patter.2023.100862)
Supplement: Document S2. Article plus supplemental information [file mmc2.pdf]

Article

Patterns

# A multi-scale unified model of human mobility in urban agglomerations

## Highlights

- We propose a multi-scale unified model of human mobility in urban agglomerations
- A cascaded deep neural network is used to simulate human travel choice processes
- A generative adversarial network is used to extract population attractiveness features
- The model can predict multi-scale travel patterns for individuals and populations

## Authors

Yong Chen, Haoge Xu,
Xiqun (Michael) Chen, Ziyou Gao

## Correspondence

chenxiqun@zju.edu.cn (X.C.),
zygao@bjtu.edu.cn (Z.G.)

## In brief

One major weakness of current human mobility models is their inability to accurately measure and quantify large-scale human movements between regions in urban agglomerations. By simulating people's mental representations of physical space, a novel multi-scale unified model based on cascaded deep neural networks is proposed for human mobility prediction within urban agglomerations. The model simulates people's hierarchical travel selection behavior and can reproduce the universal-scale laws of individuals and populations in urban agglomerations with thousands of regions.

Check for updates

Chen et al., 2023, Patterns *4*, 100862
November 10, 2023 © 2023 The Author(s).
https://doi.org/10.1016/j.patter.2023.100862

CellPress

Patterns

CellPress
OPEN ACCESS

Article

# A multi-scale unified model of human mobility in urban agglomerations

Yong Chen,[1] Haoge Xu,[1] Xiqun (Michael) Chen,[1,2,4,*] and Ziyou Gao[3,*]
[1]Institute of Intelligent Transportation Systems, College of Civil Engineering and Architecture, Zhejiang University, Hangzhou 310058, China
[2]Zhejiang University/University of Illinois Urbana-Champaign (ZJU-UIUC) Institute, Haining 314400, China
[3]School of Systems Science, Beijing Jiaotong University, Beijing 100044, China
[4]Lead contact
*Correspondence: chenxiqun@zju.edu.cn (X.C.), zygao@bjtu.edu.cn (Z.G.)
https://doi.org/10.1016/j.patter.2023.100862

**THE BIGGER PICTURE** As urban areas develop, neighboring cities gradually converge to form highly integrated urban spatial forms through a process known as urban agglomeration. Within urban agglomerations, travel occurs at different spatial scales, for example, within local neighborhoods, within cities, or between cities. This makes understanding human mobility in urban agglomerations inherently complex. While various models have been developed in the past to describe human mobility, they generally cannot model and predict the complex multi-scale travel that occurs within urban agglomerations. Methods that can better model mobility in complex urban agglomerations could have significant practical implications for topics such as urban resource management, disease control, and transportation hub optimization.

1 2 3 4 5 **Proof-of-Concept:** Data science output has been formulated, implemented, and tested for one domain/problem

## SUMMARY

Understanding human mobility patterns is vital for the coordinated development of cities in urban agglomerations. Existing mobility models can capture single-scale travel behavior within or between cities, but the unified modeling of multi-scale human mobility in urban agglomerations is still analytically and computationally intractable. In this study, by simulating people's mental representations of physical space, we decompose and model the human travel choice process as a cascaded multi-class classification problem. Our multi-scale unified model, built upon cascaded deep neural networks, can predict human mobility in world-class urban agglomerations with thousands of regions. By incorporating individual memory features and population attractiveness features extracted by a graph generative adversarial network, our model can simultaneously predict multi-scale individual and population mobility patterns within urban agglomerations. Our model serves as an exemplar framework for reproducing universal-scale laws of human mobility across various spatial scales, providing vital decision support for urban settings of urban agglomerations.

## INTRODUCTION

With the urban economy's rapid development, megacities, metropolitan areas, and their neighboring cities are gradually converging to form highly developed integrated urban spatial forms,[1] known as the urban agglomeration.[2] Each integrated city is contained in a compact space and maintains close economic ties within its urban agglomeration. Furthermore, as one of the forces most behind global economic development,[3] urban agglomerations have affected countries' strategic layouts and global competitiveness.[1,4] Accordingly, they have drawn increasing attention from governmental authorities worldwide, and various outlines and strategies[5–7] have been formulated to promote industrial cooperation, resource management and optimization, and complementary infrastructure between cities to comprehensively integrate urban agglomerations.

Within urban agglomerations, people are driven by various travel needs to conduct activities at different spatial scales. Such inter- or intra-city movements and interactions promote economic circulation and cultural exchange between cities in urban agglomerations.[8] Consequently, understanding human mobility in urban agglomerations is important and has significant


This is an open access article under the CC BY license (http://creativecommons.org/licenses/by/4.0/).

practical implications, such as controlling the spread of disease,[9,10] optimizing transportation hubs,[11] and assisting emergency management.[12] Over the past few decades, various models[13–19] have been proposed to explain the universal laws of human mobility. Classical models focus on predicting human mobility at different scales, such as the gravity model (GM),[20] radiation model[16] (RM), population-weighted opportunities model (PWO),[21] and related extension models.[22,23] In addition, fine-grained models have been developed to capture individual micro-level travel behavior, such as the exploration and preferential return model (EPR),[15] container model,[24] and universal model of individual and population (UMIP).[25]

Human mobility within urban agglomerations encompasses a wide range of spatial scales, such as intracity, intercity, and interstate. The dynamics of travel behavior are implicitly confined by geographical boundaries, administrative divisions, and transportation facilities,[26,27] and individuals exhibit distinct mobility patterns across different spatial scales.[28] Notably, additional scales raise the challenge of modeling human mobility by adding, for example, higher computational dimensions, and more complicated travel choices. With a few exceptions,[24,29] existing studies[3,30,31] have been limited to analyzing and predicting either single-scale intercity or intracity mobility within urban agglomerations. However, when modeling human mobility between communities in different cities, which involves both city and community scales, the number of included regions dramatically expands according to the number of cities or communities. Moreover, it is challenging to characterize complex multi-scale travel patterns using traditional models (e.g., GM and RM). For example, owing to the constraints of administrative divisions, the travel probability between two neighboring communities in the same city (i.e., intra-city travel) may deviate considerably from that between two neighboring communities in different cities (i.e., inter-city travel). When an individual selects a county for travel, the subsequent selection of communities within the target county may be slightly influenced by the distance from the origin. In this multi-scale mixed travel scenario, GM's distance decay effect and RM's intervening opportunity effect become less effective. These inherent challenges[22,32] underscore the necessity and complexity of multi-scale unified human mobility modeling.

From a methodological perspective, existing studies[22,23] have mainly focused on analyzing the effect of geographical features (e.g., population, points of interest, and distance) on human travel behavior to improve mobility prediction accuracy rather than on designing and expressing human-like behavior selection mechanisms. For example, people's mental representations of physical space show a clear hierarchical structure[33] embodied in the characterization, judgment, and selection of spatial regions.[34,35] We usually describe the spatial location of a place hierarchically in a nested organizational structure using typical spatial scales, such as state, county (city), community, and street information.

To address these issues, we propose a multi-scale unified model (MSUM) to achieve a unified human mobility prediction at multiple spatial scales in urban agglomerations. To characterize the human hierarchical travel choice process, MSUM embeds a cascaded multi-class classifier based on deep convolutional neural networks[36] (CNNs), which is an extension of the nested logit (NL) model.[37] Individual historical memory and population attractiveness features are used as classifier inputs, and by adding convolutional layers and nonlinear activation layers, complex human mobility features can be automatically extracted. Furthermore, to derive population attractiveness features, we introduce a graph generative adversarial network[38] (GGAN) to extract the trip distribution at single spatial scales (e.g., county and community scales). Unlike traditional mobility models (e.g., GM and PWO), which are based on distance or intervening opportunity, we formulate trip distribution prediction as a missing data imputation problem. In GGAN, the generator is used to impute the missing travel probabilities between locations with unknown travel volumes. The discriminator is used to discriminate between observable and missing components. Through adversarial training between the two, the nonlinear dependencies between travel probability and urban indicators (e.g., distance and population) can be extracted. We thereby calculate the population attractiveness of the target location by summing its probabilities of being visited by other locations, as generated by GGAN. Finally, by training MSUM using empirical human mobility data, the model can simultaneously capture the scaling laws of individuals and populations across multiple spatial scales within urban agglomerations, aligning well with empirical findings.

## RESULTS

### MSUM for human mobility prediction in urban agglomerations

MSUM is inspired by the classical NL model[37] based on the random utility theory to model individual choice behavior. The NL model creates a nested structure (see Figure S2 and Note S1 for details) to depict the correlation between alternatives and to avoid independence of irrelevant alternatives in the traditional logit model.[39] Each layer of the NL model can be regarded as a multinomial logit (MNL) model[40] with different attributes linked by conditional probability and utility feedback. Studies[41] have shown that an MNL model is equivalent to a CNN with $\mathbf{X}$ as the feature input, $\boldsymbol{\beta}$ as the filter parameter, and *softmax*[42] as the nonlinear activation function. This finding led us to extend the original NL model to a deep learning-based cascaded multi-class classification model (the MSUM, Figure 1) to achieve deep extraction of nonlinear mobility features and unified prediction of multi-scale human mobility in urban agglomerations.

In MSUM, individual travel choice at multiple spatial scales within urban agglomerations is regarded as the process of hierarchically selecting the correct class with the greatest utility from candidate locations (Figure 1A). That is, the urban agglomeration can be divided into different spatial scales (e.g., state, county, and community, depending on dataset granularity; see data description and preprocessing for details). The individual first selects a class (state) in the large-scale (e.g., state scale) regional candidate set, and then selects a medium-scale class (e.g., county) under the selected region to travel. Meanwhile, the large-scale region nests all corresponding small-scale regions. The selection (classification) process at different spatial scales is completed using different deep learning-based classifiers (Figure 1B; see implementation of MSUM and Note S2 for details), which are mutually constrained by the classification probability and classification error. The feature input of the classifier

CellPress
OPEN ACCESS

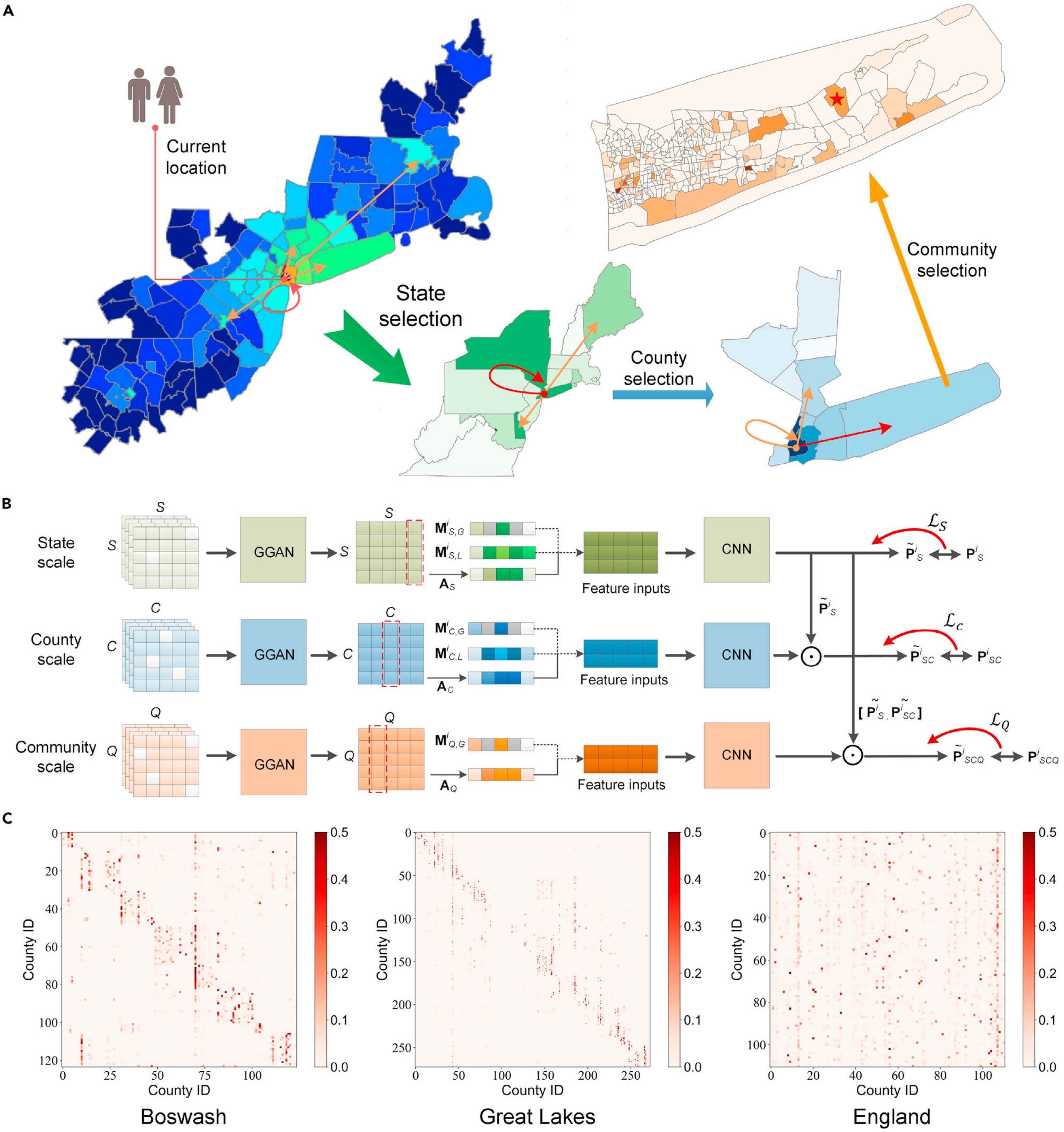


**Figure 1. Multi-scale unified model of human mobility in urban agglomerations**

(A) Individual's hierarchical travel choice process. Individuals make travel choices hierarchically from the large (e.g., state) to the small scale (e.g., community) following the corresponding choice probability. The color of each region indicates the corresponding selection probability.

(B) Multi-scale unified model architecture. The model's input includes individual memory features ($\mathbf{M}_L^i$ and $\mathbf{M}_G^i$) and population attractiveness features (**A**). The population attractiveness feature is obtained by the graph generative adversarial network (GGAN) (see Note S3 for details). Based on the feature extraction of a deep convolutional neural network (CNN), the model outputs the classification probability of different spatial scales layer by layer. The classification probability of the upper layer of the model multiplies the probability of the lower layer to achieve conditional constraints. The classification error of the lower layer propagates to the upper layer to participate in updating CNN parameters.

(C) Travel probabilities between counties in three urban agglomerations predicted by GGAN. Darker colors indicate greater inter-county travel probability.

includes the individual memory and population attractiveness features. The memory feature indicates that individuals strongly prefer to return to locations they have visited before, and the degree of this preference is proportional to the frequency of visits.[15] The attractiveness feature indicates that a location's popularity is proportional to the overall probability of being visited—an attractive location is visited by more people from other locations. We divide individual memory features into global memory features ($\mathbf{M}^i_G$) and local memory features ($\mathbf{M}^i_L$). Taking the county scale (i.e., scale $C$) as an example, the global memory feature $\mathbf{M}^i_{C,G}$ represents the visit frequency of individual $i$ to other counties, regardless of the origin county. The local memory feature $\mathbf{M}^i_{C,L}$ represents the visit frequency of individual $i$ to other counties from the current county. To obtain the population attractiveness feature vector $\mathbf{A}_C$, we propose a GGAN (see Note S3 and Figure S1 for details) to predict human trip distribution at different spatial scales. GGAN adversarially learns the parameters of the graph neural network through the two core tasks of data imputation and missing discrimination, and outputs the travel probability between each county (Figure 1C). The generator aims to impute missing travel probabilities between counties based on existing observation data, whereas the discriminator aims to distinguish which travel probabilities are generated and which are observed. Finally, the county's attractiveness can be obtained by accumulating the travel probabilities from other counties to the target county.

### Population attractiveness feature extraction

GGAN is proposed to accurately and robustly predict the single-scale (i.e., inter-county and intra-county) human trip distribution in urban agglomerations, and it can provide more reliable population attractiveness features for MSUM compared with benchmark models (i.e., GM, RM, and PWO; see supplemental experimental procedures for model details). We conduct a series of comparison experiments in three world-class urban agglomerations: Boston-Washington (Boswash) (USA), Great Lakes (USA), and England (UK) urban agglomerations. These three urban agglomerations encompass different historical, cultural, educational, and economic contexts, which can provide diverse and comprehensive perspectives for understanding mobility patterns within urban agglomerations. They cover 124 (Boswash) and 273 (Great Lakes) counties in the USA and 111 (England) in the UK. We regard *county* as the primary spatial scale in the urban agglomeration, and further divide the corresponding counties into 7,731, 15,156, and 10,114 non-overlapping square regions according to their area to represent the secondary spatial scale (i.e., *community* scale). The mobility data for the Boswash and Great Lakes urban agglomerations are user check-in data from the website Weeplaces,[43] which consists of travel traces of 4,952 and 2,033 individuals, respectively. The mobility data for the England urban agglomeration are the user check-in data from the location-based social networking website Gowalla,[44] which consists of travel traces of 3,193 individuals (see data description and preprocessing for details). Because of the coverage of the mobile data used, we selected three of the top 5 world-class urban agglomerations[45,46] as the research areas. Nonetheless, our proposed model can be generalized to urban agglomerations worldwide. The human mobility networks in the three urban agglomerations are shown in Figure 2. People's mobility patterns at different spatial scales have obvious heterogeneity and hierarchy. The intra-county travel network (i.e., inter-community travel) is more intensive than the inter-county travel network. Travel distance characteristics at different scales are shown in Figure 2D.

We use the random missing method to construct the training and test sets for model comparison (see model training and evaluation for details). Figure 1C shows the travel probabilities between counties in the three urban agglomerations as predicted by GGAN. We find that travel between counties in the Boswash and Great Lakes urban agglomerations is greatly affected by state borders, and there is a clear hierarchical organization. For the England urban agglomeration, counties are the first-level administrative divisions, so travel between counties is more uniform. Using the common part of commuters[22,25] (CPC) to quantify the similarity between real and predicted flows, Figure 3 shows that GGAN has the best prediction performance at different spatial scales in the three urban agglomerations. Note that CPC values range between 0 and 1, with higher values indicating a better match between the predicted and actual flows. For a more comprehensive model comparison, we use the mean absolute error and root mean-square error (RMSE) to evaluate model performance (see supplemental experimental procedures and Tables S1–S3 for details). As shown in Figures 3A–3C, the first column (labeled "County") shows the performance evaluation results of travel flow prediction between counties in urban agglomerations. For the Great Lakes urban agglomeration, the CPC value of GGAN (CPC = 0.505) is 0.370, 0.069, and 0.234 higher than those of GM, RM, and PWO, respectively (see Table S2 for details).

The second column (labeled "Ave.") shows the average performance evaluation results of the inter-community travel flow predictions for all counties. Similar to the county scale, the overall prediction performance of GGAN is better than that of the other two baseline models. The distribution of CPC values for each model at the community scale is shown in Figures 3D–3F. As shown in the last 10 columns (labeled by county abbreviation) of Figures 3A–3C, we select the top 10 counties (Figure 4) in the three urban agglomerations in terms of travel volume to visualize each model's prediction performance at the community scale. Most selected counties (e.g., New York, Cook, Manchester, and Birmingham) are core political, economic, and cultural areas in the USA and the UK. The free flow of various production factors in these counties has promoted the rapid development of urban agglomerations. For a performance comparison, GGAN has the highest prediction accuracy, and is significantly better than the baseline models. For instance, in the District of Columbia (labeled "DC") of the Boswash urban agglomeration (see Table S1 for details), the CPC value of GGAN (CPC = 0.806) is 0.358, 0.275, and 0.150 higher than those of GM, RM, and PWO, respectively.

To investigate the model's prediction performance in different active regions, we divide the counties in each urban agglomeration into 10 groups based on county travel volume and rank them in descending order. We then calculate the prediction performance of the trip distribution at the community scale for each group (see Figures 3G–3I and Table S4 for details). In the three urban agglomerations, the prediction performance of all models degrades with a decrease in travel volume. This is because trip

CellPress
OPEN ACCESS

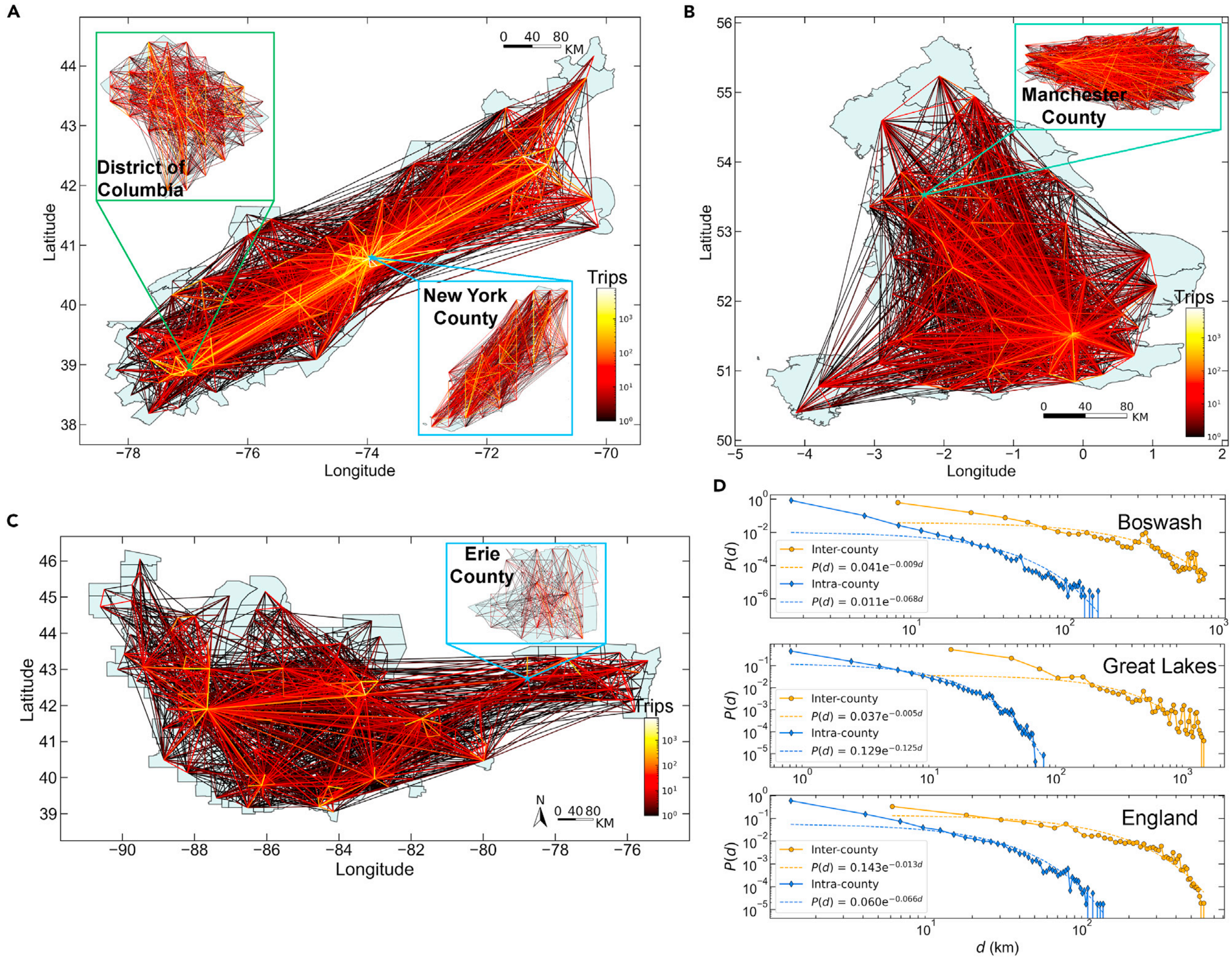


**Figure 2. Human mobility networks of three urban agglomerations**

(A) Distribution of human mobility among 124 counties in the Boston-Washington (Boswash) urban agglomeration, USA.

(B) Distribution of human mobility among 111 counties in the England urban agglomeration of the UK.

(C) Distribution of human mobility among 273 counties in the Great Lakes urban agglomeration, USA. Line color indicates the intensity of the travel flows between regions, with brighter (darker) colors indicating stronger (weaker) travel flows.

(D) Travel distance distribution of inter-county and intra-county trips in the three urban agglomerations.

distribution becomes sparse and random in low-activity counties, making it difficult for the model to accurately capture mobility features based on current travel information. Nevertheless, GGAN maintains remarkable prediction accuracy across groups and significantly outperforms the baseline models.

## Multi-scale human mobility prediction

MSUM can predict multi-scale mobility patterns within urban agglomerations. We use a portion of actual individual traces to train the model (see model training and evaluation for details) and simulate the subsequent continuous individual traces (see simulation of individual traces for details). At each step, an individual selects a community within a specific county for travel based on the MSUM classification results. The degree of authenticity of the individual and population mobility patterns captured by MSUM can be verified by comparing the simulated traces with unseen data. To explore the accuracy of the model, the EPR model,[15] three EPR variant models,[17,47,48] and the UMIP model are selected for performance comparison (see supplemental experimental procedures for details).

At the individual level, we focus on three essential scaling laws (Figure 5): the total number of locations visited in $t$ trips, the frequency distribution of individuals visiting a location, and the distribution of the radius of gyration.[14] For the three urban agglomerations, the empirical data (green) in Figures 5A–5C show that the number of locations visited by individuals increases algebraically rather than exponentially over time owing to the influence of the individual memory effect. In a travel environment involving multiple spatial scales, our model (red) precisely matches the empirical results, whereas the other baseline models overestimate them even though all consider the individual's historical memory. Accordingly, Figures 5D–5F show the frequency distribution of visits to a location, which is power-law decreasing. Consistent with empirical research,[17]

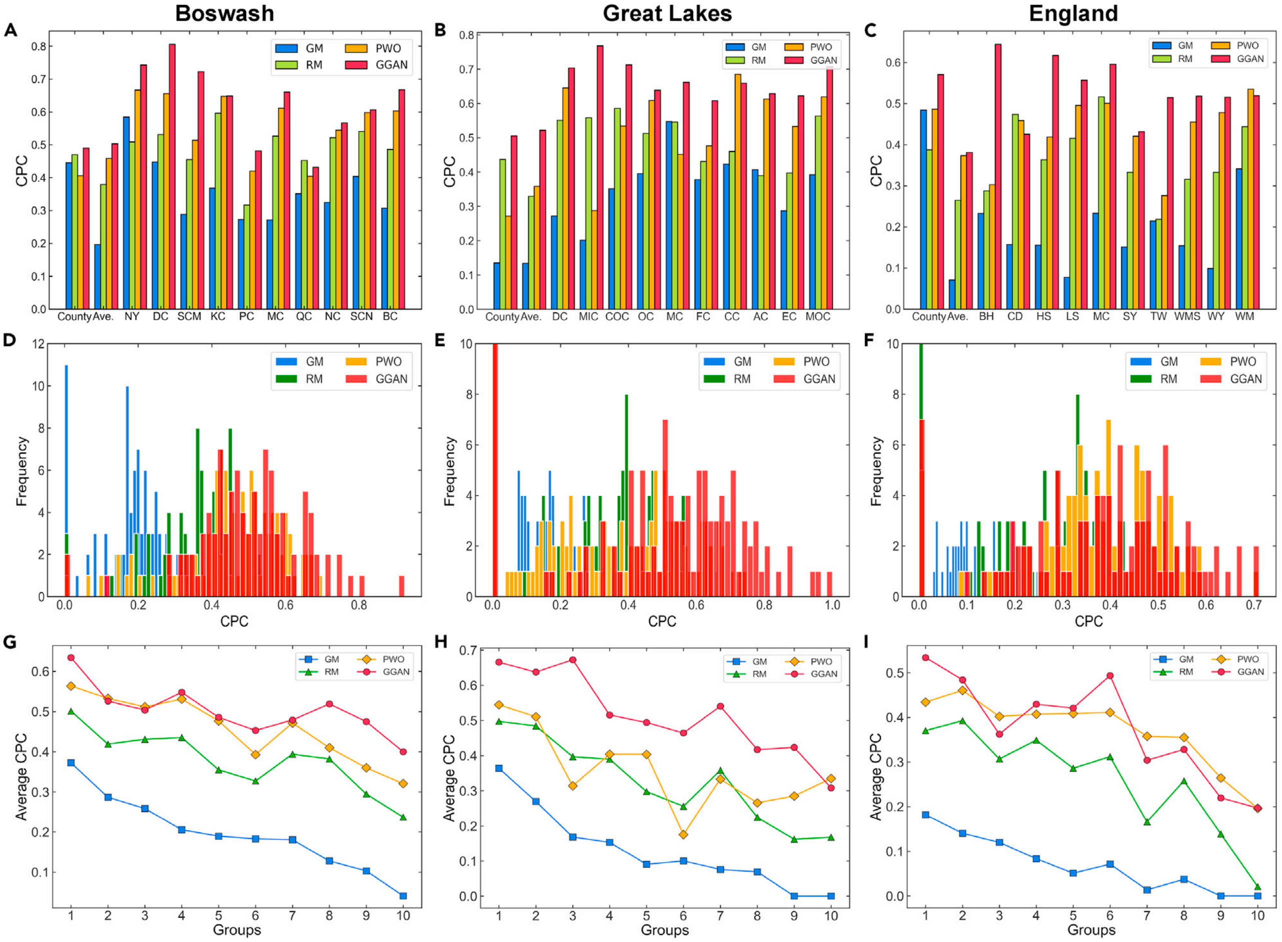


**Figure 3. Performance comparison of single-scale human mobility prediction in terms of common part of commuters**

(A–C) Performance comparison of the gravity model (GM), radiation model (RM), population-weighted opportunities model (PWO), and graph generative adversarial network (GGAN) at the county and community scales. The first column (labeled "County") indicates the model's accuracy in predicting travel flows between counties. The second column (labeled "Ave.") indicates the average accuracy of the model in predicting inter-community travel flows for all counties within the urban agglomeration. The last 10 columns (labeled by county abbreviation) represent the inter-community travel flow prediction accuracy of GM, RM, PWO, and GGAN in the 10 counties with the highest travel volume.

(D–F) Distribution of common part of commuters values for travel flow prediction at the community scale.

(G–I) Prediction accuracy of GM, RM, PWO, and GGAN in different active regions. In each urban agglomeration, all counties are evenly divided into 10 groups and ranked in descending order based on travel volume. Each point indicates the average prediction accuracy of the corresponding model under each group.

people visit only a few locations regularly, such as home and work. Again, MSUM provides a scale description that is more consistent with the empirical distribution, whereas the baseline models underestimate the visiting frequency. Among the baseline models, UMIP performs best for the Great Lakes and England urban agglomerations, demonstrating the necessity of considering population travel information in multi-scale mobility modeling in addition to individual memory features. Figures 5G–5I show an exponentially decreasing trend in the radius of gyration. A comparison of the different models shows that our model can more accurately describe individual travel preferences, whether short or long distance. Moreover, by aggregating the simulation traces of all individuals, individual mobility patterns at a single scale (e.g., inter-county travel) can be quantified (see supplemental experimental procedures, Figures S5–S7, and Table S6 for details).

At the population level, we quantify the prediction performance of MSUM using two scaling laws (Figure 6): the travel distance distribution of the population, and the trip number distribution between two locations. Figures 6A–6C show the probability distribution of people traveling different distances, which decreases exponentially. Our model has good prediction performance for short trips and a slight overestimation for long trips, but it is a significant improvement over the baseline models. Figures 6D–6F show another important scaling law describing the probability distribution of the number of trips between two locations. Compared with the baseline model, our model can robustly predict the scaling laws of algebraic decay in the three urban agglomerations and is in excellent agreement with the empirical distribution. The baseline model significantly underestimates the number of trips between locations, except for UMIP. In addition, as shown in Figures 6G–6I, a pairwise comparison of the predicted trips with the observed

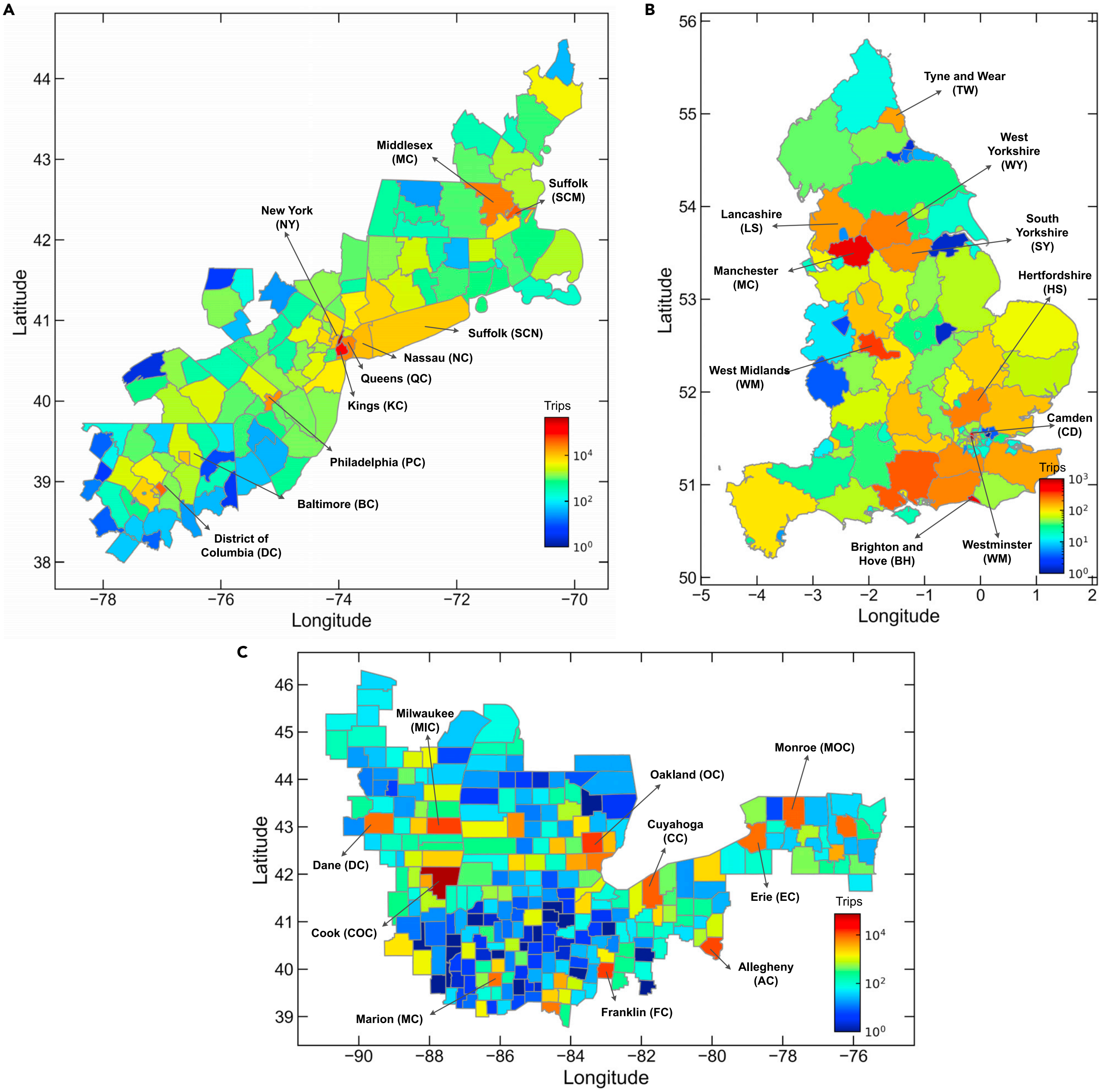


**Figure 4. Selection of the top 10 counties in the three urban agglomerations regarding travel volume**

Top 10 counties in (A) Boswash, (B) England, and (C) Great Lakes urban agglomerations. Letters in parentheses indicate county name abbreviations. The color of each region indicates the number of trips originating from the corresponding region, including intra- and inter-county trips.

trips shows that the model-predicted and actual trips are statistically indistinguishable (see Figure S4 for details). The effectiveness measures show that the CPC values of MSUM in the three urban agglomerations are 0.294 (Boswash), 0.098 (Great Lakes), and 0.098 (England) higher than that of the optimal baseline model (see Table S5 for details). For instance, compared with the best-performing memory EPR (M-EPR) model for the Boswash urban agglomeration, the CPC and RMSE values of MSUM increased by 77.78% and 44.55%, respectively. Based on the above scale laws and model performance analysis, our model can effectively predict multi-scale travel patterns within urban agglomerations by simulating the hierarchical travel choice behavior of human beings, and coupling individual memory and population attractiveness features, thereby improving the accuracy and robustness of travel prediction.

## DISCUSSION

We propose MSUM to predict human mobility in urban agglomerations at multiple spatial scales. By considering the hierarchical

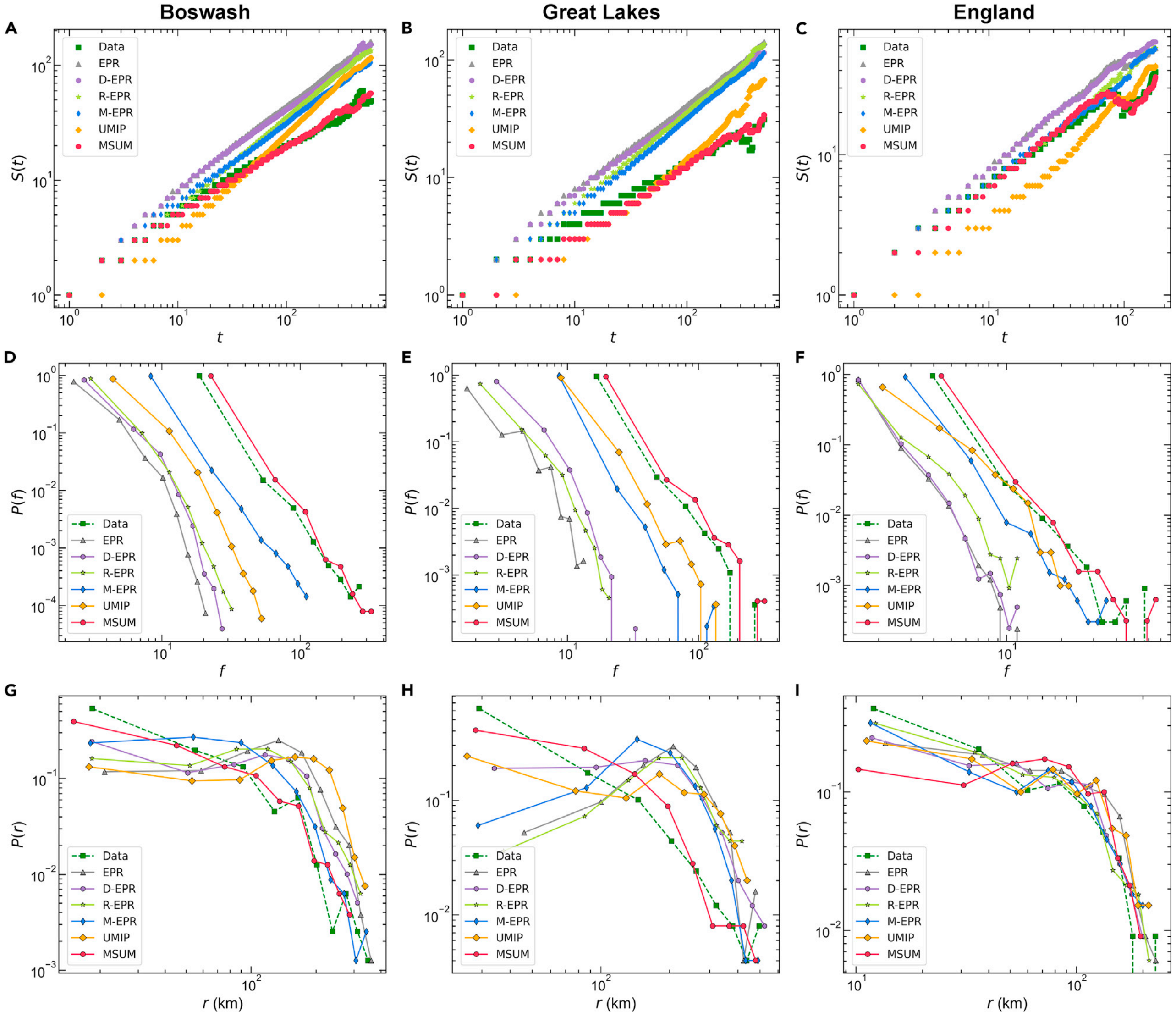


**Figure 5. Multi-scale human mobility prediction in urban agglomerations at the individual level**

(A–C) Distribution of the total number of locations visited in *t* trips.

(D–F) Frequency distribution of individuals visiting a location.

(G–I) Radius of gyration distribution. All distributions are calculated from the simulated (see simulation of individual traces for details) and actual individual traces.

nature of human travel choice behavior, our model effectively reproduces the scaling laws of individual and population mobility between communities in different counties in urban agglomerations. In our model, GGAN can accurately and robustly predict the single-scale (i.e., inter- and intra-county) mobility patterns of different active regions compared with state-of-the-art population mobility models, and it provides a reliable characterization of location attractiveness. For the mobility prediction at multiple spatial scales, taking the Great Lakes urban agglomeration as an example, the human activity space increases from 273 to 15,156 regions. This increase of spatial scale leads to sparsity of data and uncertainty of human activities, amplifying the difficulty of accurate mobility prediction. By simulating the human behavior selection mechanism (i.e., hierarchical mental representation of physical spaces) to construct a cascaded multi-class classification framework, and leveraging the powerful feature extraction capability of deep neural networks, our MSUM can implicitly reduce the solution space of travel choice layer by layer, thereby accurately capturing various scaling laws in accordance with empirical data, such as the radius of gyration distribution, trip distance distribution, and trip number distribution between two locations.

As the main form of settlement in modern human society, understanding the multi-scale mobility patterns of individuals and populations within urban agglomerations can support decision-making for various urban settings. In particular, our model can precisely describe travel behavior at the individual level, unlocking unprecedented possibilities for refined realistic simulation, epidemic prevention and control prediction, regional development, urban planning, transportation planning, etc. For example,

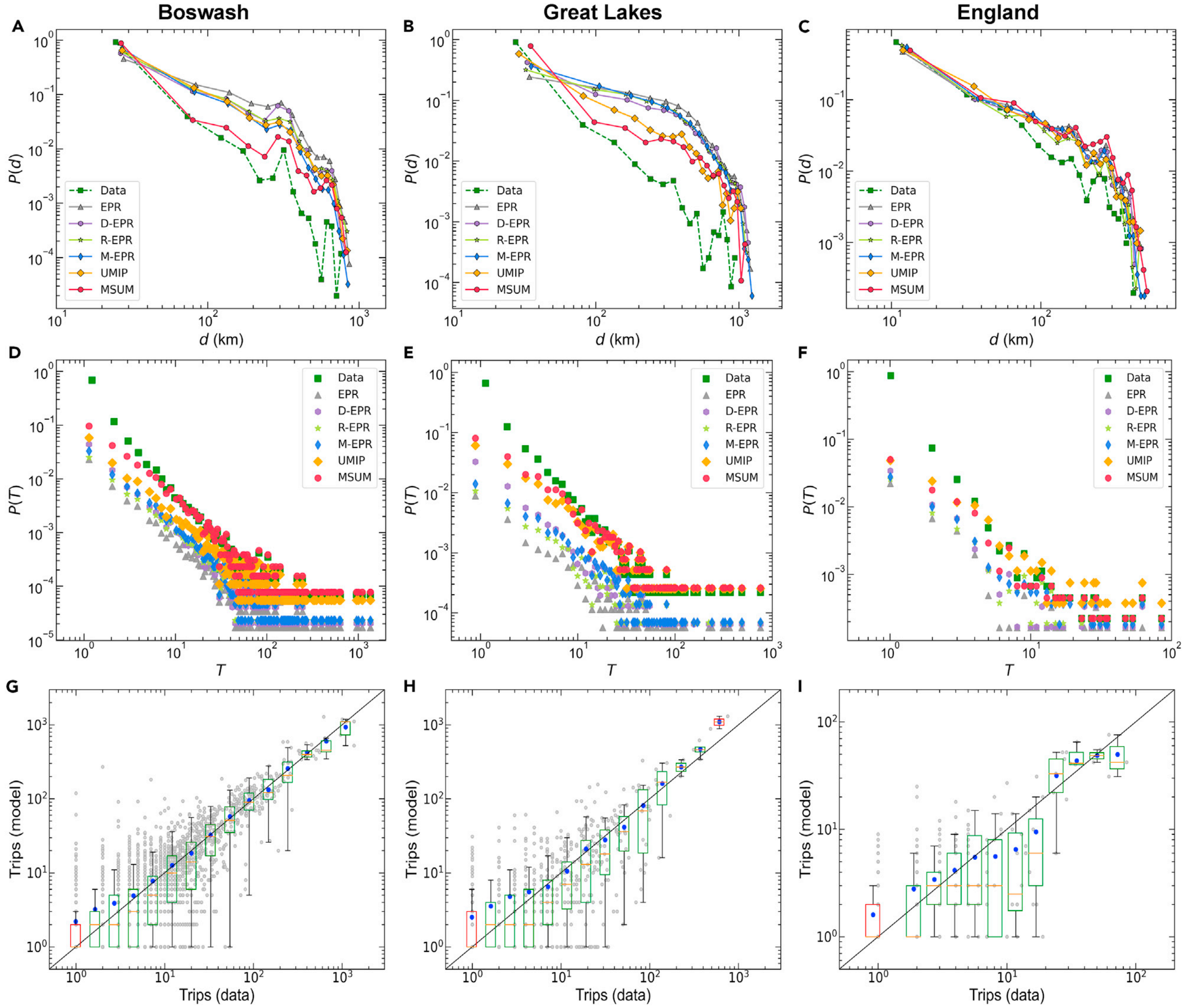


**Figure 6. Multi-scale human mobility prediction in urban agglomerations at the population level**

(A–C) Predicted and real distributions of travel distance.

(D–F) Predicted and real distributions of the number of trips between two locations.

(G–I) Paired comparison of predicted and real trips. Gray points indicate observed and predicted location pairs. Blue points indicate the average number of predicted trips in different bins. The boxplot indicates the distribution of the number of predicted trips in different bins of the number of observed trips. A box is green if the black line $y = x$ is between the 5th and 95th percentiles of the box, and red otherwise. To characterize the model's prediction performance at the population level, all distributions are calculated by aggregating simulated and actual individual traces.

the quantitative evaluation results demonstrate that our model can predict travel flow between large-scale regions (e.g., over 10,000 regions) more accurately than state-of-the-art individual mobility models. Regarding infrastructure construction, urban decision-makers can identify underdeveloped areas within the urban agglomeration based on the frequency of travel visits between regions and make direct investments to promote balanced growth and development. Simultaneously, the multi-scale mobility prediction results help identify popular tourist destinations and routes for tourism-driven cities. By understanding where individuals spend the most time, tourism managers can focus on improving infrastructure and providing better services, such as providing more passenger corridors or strengthening measures in regions with frequent travel to avoid congestion caused by a large number of trips. Regarding transportation planning, trip distribution prediction results at different spatial scales can assist managers in building intelligent transportation systems. The prediction results of short-distance trips can help optimize the design and operation of public transportation lines within urban agglomerations, ensure that public transportation services are close to citizens' actual needs, and improve public transportation system efficiency and convenience. With long-distance trip distribution prediction results, managers can formulate and optimize transportation networks based on travel needs and recommend multi-modal travel services[49] to improve travel efficiency. Moreover, by analyzing the multi-scale travel

flow patterns in urban agglomerations, urban planners can anticipate regions likely to experience significant population growth and urban sprawl. This information is vital for making informed decisions on zoning, land-use regulations, and urban expansion plans, ensuring that new developments align with the growing population's needs.

Our study has some limitations that should be addressed in future research. MSUM extends the classical NL model by constructing a cascaded deep CNN framework to simulate and express the mechanism of human hierarchical travel choices. The interpretability of deep neural networks remains a key challenge in advancing the understanding of probabilistic relationships between input features (e.g., individual memory and population attractiveness) and corresponding travel choices. The abstract feature information extracted by GGAN and deep CNN can be analyzed by future studies using game theory, agnostic,[50,51] and other techniques. In addition, we did not consider the effects of finer administrative boundaries and geographical contextual features (e.g., points of interest) on human travel. However, these features are closely related to people's daily movement and can be used as the feature input of the prediction model in future research.

## EXPERIMENTAL PROCEDURES

### Resource availability

#### *Lead contact*

Further information and requests for resources and reagents should be directed to and will be fulfilled by the lead contact, Xiqun (Michael) Chen (chenxiqun@zju.edu.cn).

#### *Materials availability*

This study did not generate new unique materials.

#### *Data and code availability*

The mobility data are publicly available at http://snap.stanford.edu/data/loc-gowalla.html and https://www.yongliu.org/datasets/. National administrative area data for the USA and UK are publicly available at https://gadm.org/maps.html. Population data for the USA and UK are publicly available at https://www.census.gov/quickfacts/fact/table/US/POP010210 and https://www.ons.gov.uk/visualisations/censuspopulationchange/, respectively. The MSUM source code is available from Zenodo (https://doi.org/10.5281/zenodo.8175023).[52] The other data relevant to this study are available upon request.

### Data description and preprocessing

Regional boundary data for the Boswash, Great Lakes, and England urban agglomerations were provided by the American Regional Planning Association[53] and the European Union.[54] The Boswash and Great Lakes urban agglomerations cover 124 and 273 counties in the USA, respectively. The England urban agglomeration covers 111 counties. County-scale administrative boundary data were downloaded from the GADM open-source platform (https://gadm.org/index.html). To represent the finer scale regions (i.e., community scale), we used the Python library scikit-mobility[55] to divide each county into multiple non-overlapping square regions. Assuming that the area of a county is $A$, the side length of each square region is set to $\sqrt{A}/5$, which ensures uniformity of the fine-scale boundary division. Thus, the three urban agglomerations contain 7,731 (Boswash), 15,156 (Great Lakes), and 10,114 (England) communities.

All experimental analyses were based on two check-in datasets collected from publicly available location-based social networks. The first dataset was collected by Gowalla[44] and consists of 36,001,959 check-ins generated by 319,063 users worldwide from February 2009 to October 2010. The second dataset was collected by Weeplaces,[43] which integrates the application programming interfaces of multiple location-based social networking services (e.g., Foursquare and Gowalla) and consists of 7,658,368 check-ins of 15,799 users from November 2003 to June 2011. We filter successive points that repeatedly stay at the same location and users who travel too little to avoid data interference. After mapping check-in coordinates to the corresponding urban agglomerations, Boswash contains 1,016,454 trips from 4,952 users, Great Lakes contains 285,089 trips from 2,033 users, and England contains 111,772 trips from 3,193 users. The trip distribution characteristics of the individual traces are shown in Figure S3. Moreover, population data are publicly available from the United States Census Bureau[56] and the Office for National Statistics.[57]

### MSUM implementation

The specific implementation process of MSUM includes the following three steps (Figure 1B).

(1) Individual memory and population attractiveness features are concatenated as feature inputs of MSUM for feature extraction. Specifically, to consider an individual's current location dynamically, we divide the individual memory features into global ($\mathbf{M}_G^i$) and local memory features ($\mathbf{M}_L^i$). The global memory feature represents the historical visit frequency of individual $i$ to other locations regardless of the origin. The local memory feature represents the historical visit frequency of individual $i$ from the current location to other locations. To obtain the population attractiveness feature vector $\mathbf{A}_C$, a GGAN is proposed to generate a trip distribution matrix at different scales. The location's attractiveness can be obtained by accumulating the travel probabilities from other locations to the target location.
(2) The concatenation vectors are input into CNN (see Note S2 for details) without bias parameters. As the input feature is a two-dimensional matrix, the one-dimensional convolution filter is used to construct CNN for feature extraction. At different spatial scales, CNN has a similar network framework, but has different network parameters.
(3) The model output is a vector representing the classification results at the corresponding scale. The vector length equals the total number of classes under the corresponding scale; the smaller the scale, the larger the vector length. To associate the selection results of different scales, the classification probability output of the upper CNN is multiplied by that of the lower CNN to achieve the constraint of hierarchical selection. For example, when an individual has a low probability of selecting a county, s/he is less likely to select a community within that county. Moreover, the classification error of the lower-layer CNN propagates to the upper layer, affecting and participating in updating the network parameters of the upper-layer CNN.

### Simulation of individual traces

Given the trained MSUM, we can simulate individual travel traces using the following three steps.

(1) Assuming that the total number of movement steps of individual $i$ in the empirical test dataset is $L^i$, we use the first $2L^i/3$ movement steps to calculate the historical memory features of the individual at different spatial scales, including global $\mathbf{M}_G^i$ and local memory features $\mathbf{M}_L^i$. Based on the trained GGAN, population attractiveness feature $\mathbf{A}$ at different spatial scales can also be obtained.
(2) The location after $2L^i/3$ movement steps is taken as the initial location of individual $i$. The local memory feature vector starting from the current location, global memory feature vector, and population attractiveness feature vector are concatenated and input into MSUM. Individual $i$ chooses a location to travel in the multi-scale space of urban agglomerations by referring to the multi-scale selection probability distribution output by MSUM.
(3) The individual's historical memory feature vector is updated. Repeat step (2) to complete the individual trace simulation with $2L^i/3$ movement steps.

### Model training and evaluation

For GGAN, we construct a test set by setting a random missing rate of 20% to mask the travel volumes between some locations as missing data. For the non-missing 80% of the data, we generate sufficient training samples by repeatedly

performing random masking of 10% of the travel volume information. GGAN is trained for 1,000 epochs using the Adam optimizer[58] with a learning rate of 0.001. To compare the model performance, state-of-the-art GM,[22] RM,[16] and PWO[21] are implemented as baseline models (see supplemental experimental procedures for details). For MSUM, we use a stratified sampling method to construct the training and test sets with a ratio of 8:2 based on the individual's movement steps. For each individual, we use the first two-thirds of the data to calculate the individual's initial memory features, and sequentially update the memory features based on the empirical data to generate training samples, during which the real destination is used as the training label of the model. The entire MSUM is trained for 30 epochs using the Adam optimizer, with a learning rate of 0.001. The cross-entropy function is used as the loss function to evaluate the performance of the network parameters. For a more comprehensive comparison, four individual mobility models are also implemented as baseline models (see supplemental experimental procedures for details): EPR, gravity EPR[17] (D-EPR), recency EPR[47] (R-EPR), M-EPR[48], and UMIP.[25]

## SUPPLEMENTAL INFORMATION

Supplemental information can be found online at https://doi.org/10.1016/j.patter.2023.100862.

## ACKNOWLEDGMENTS

This research is financially supported by the National Natural Science Foundation of China (72288101 and 72171210), Zhejiang Provincial Natural Science Foundation of China (LZ23E080002), National Key Research and Development Program of China (2020AAA0107401), and the Smart Urban Future (SURF) Laboratory, Zhejiang Province.

## AUTHOR CONTRIBUTIONS

Y.C., Z.G., and X.M.C. proposed the question. Y.C. and H.X. designed and conducted the experiments. Y.C., H.X., and X.M.C. developed the algorithms. Y.C., H.X., Z.G., and X.M.C. wrote the paper.

## DECLARATION OF INTERESTS

The authors declare no competing interests.

## INCLUSION AND DIVERSITY

We support inclusive, diverse, and equitable conduct of research.

Received: July 3, 2023
Revised: September 1, 2023
Accepted: September 19, 2023
Published: October 17, 2023

## REFERENCES

1. Fang, C. (2014). Progress and the future direction of research into urban agglomeration in China. Acta Geograph. Sin. *69*, 1130–1144. https://doi.org/10.11821/dlxb201408009.
2. Gottmann, J. (1964). Megalopolis: The Urbanized Northeastern Seaboard of the United States (MIT Press).
3. He, Z. (2020). Spatial-temporal fractal of urban agglomeration travel demand. Physica A *549*, 124503. https://doi.org/10.1016/j.physa.2020.124503.
4. Fang, C., and Yu, D. (2017). Urban agglomeration: An evolving concept of an emerging phenomenon. Landsc. Urban Plann. *162*, 126–136. https://doi.org/10.1016/j.landurbplan.2017.02.014.
5. MAGNO, R.A. (1972). Metropolitan region planning and development in Japan. Roy. Aust. Plann. Inst. J. *10*, 119–123. https://doi.org/10.1080/00049999.1972.9656380.
6. Lang, R.E., and Nelson, A.C. (2007). Beyond the Metroplex: Examining Commuter Patterns at the "Megapolitan" Scale (Lincoln Institute of Land Policy). White paper).
7. Fang, C. (2015). Important progress and future direction of studies on China's urban agglomerations. J. Geogr. Sci. *25*, 1003–1024. https://doi.org/10.1007/s11442-015-1216-5.
8. Paasi, A. (2004). Place and region: Looking through the prism of scale. Prog. Hum. Geogr. *28*, 536–546. https://doi.org/10.1191/0309132504ph502pr.
9. Ferreira, C.P., Marcondes, D., Melo, M.P., Oliva, S.M., Peixoto, C.M., and Peixoto, P.S. (2021). A snapshot of a pandemic: The interplay between social isolation and covid-19 dynamics in Brazil. Patterns *2*, 100349. https://doi.org/10.1016/j.patter.2021.100349.
10. Liu, L., Wang, H., Zhang, Z., Zhang, W., Zhuang, S., Wang, S., Silva, E.A., Lv, T., Chio, C.O., Wang, Y., et al. (2022). Infectiousness of places–Impact of multiscale human activity places in the transmission of COVID-19. NPJ Urban Sustain. *2*, 28. https://doi.org/10.1038/s42949-022-00074-w.
11. Dong, L., Li, R., Zhang, J., and Di, Z. (2016). Population-weighted efficiency in transportation networks. Sci. Rep. *6*, 26377. https://doi.org/10.1038/srep26377.
12. Fan, Z., Song, X., and Shibasaki, R. (2020). Big data-driven citywide human mobility modeling for emergency management. In In Big Data in Emergency Management: Exploitation Techniques for Social and Mobile Data (Springer), pp. 109–130. https://doi.org/10.1007/978-3-030-48099-8_6.
13. Brockmann, D., Hufnagel, L., and Geisel, T. (2006). The scaling laws of human travel. Nature *439*, 462–465. https://doi.org/10.1038/nature04292.
14. González, M.C., Hidalgo, C.A., and Barabási, A.L. (2008). Understanding individual human mobility patterns. Nature *453*, 779–782. https://doi.org/10.1038/nature06958.
15. Song, C., Koren, T., Wang, P., and Barabási, A.L. (2010). Modelling the scaling properties of human mobility. Nat. Phys. *6*, 818–823. https://doi.org/10.1038/nphys1760.
16. Simini, F., González, M.C., Maritan, A., and Barabási, A.L. (2012). A universal model for mobility and migration patterns. Nature *484*, 96–100. https://doi.org/10.1038/nature10856.
17. Pappalardo, L., Simini, F., Rinzivillo, S., Pedreschi, D., Giannotti, F., and Barabási, A.L. (2015). Returners and explorers dichotomy in human mobility. Nat. Commun. *6*, 8166. https://doi.org/10.1038/ncomms9166.
18. Reia, S.M., Rao, P.S.C., and Ukkusuri, S.V. (2022). Modeling the dynamics and spatial heterogeneity of city growth. npj Urban Sustain. *2*, 31. https://doi.org/10.1038/s42949-022-00075-9.
19. Schläpfer, M., Dong, L., O'Keeffe, K., Santi, P., Szell, M., Salat, H., Anklesaria, S., Vazifeh, M., Ratti, C., and West, G.B. (2021). The universal visitation law of human mobility. Nature *593*, 522–527. https://doi.org/10.1038/s41586-021-03480-9.
20. Zipf, G.K. (1946). The $P_1$ $P_2$/D hypothesis: On the intercity movement of persons. Am. Socio. Rev. *11*, 677–686. https://doi.org/10.2307/2087063.
21. Yan, X.Y., Zhao, C., Fan, Y., Di, Z., and Wang, W.X. (2014). Universal predictability of mobility patterns in cities. J. R. Soc. Interface *11*, 20140834. https://doi.org/10.1098/rsif.2014.0834.
22. Barbosa, H., Barthelemy, M., Ghoshal, G., James, C.R., Lenormand, M., Louail, T., Menezes, R., Ramasco, J.J., Simini, F., and Tomasini, M. (2018). Human mobility: Models and applications. Phys. Rep. *734*, 1–74. https://doi.org/10.1016/j.physrep.2018.01.001.
23. Simini, F., Barlacchi, G., Luca, M., and Pappalardo, L. (2021). A deep gravity model for mobility flows generation. Nat. Commun. *12*, 6576. https://doi.org/10.1038/s41467-021-26752-4.
24. Alessandretti, L., Aslak, U., and Lehmann, S. (2020). The scales of human mobility. Nature *587*, 402–407. https://doi.org/10.1038/s41586-020-2909-1.
25. Yan, X.Y., Wang, W.X., Gao, Z.Y., and Lai, Y.C. (2017). Universal model of individual and population mobility on diverse spatial scales. Nat. Commun. *8*, 1639. https://doi.org/10.1038/s41467-017-01892-8.

26. Cadwallader, M.T. (1992). Migration and Residential Mobility: Macro and Micro Approaches (University of Wisconsin Press).
27. Thiemann, C., Theis, F., Grady, D., Brune, R., and Brockmann, D. (2010). The structure of borders in a small world. PLoS One *5*, e15422. https://doi.org/10.1371/journal.pone.0015422.
28. Berry, B.J.L. (1967). Geography of Market Centers and Retail Distribution (Prentice-Hall Press).
29. Han, X.P., Hao, Q., Wang, B.H., and Zhou, T. (2011). Origin of the scaling law in human mobility: Hierarchy of traffic systems. Phys. Rev. E E. *83*, 036117. https://doi.org/10.1103/PhysRevE.83.036117.
30. Wang, Z., Ye, X., Lee, J., Chang, X., Liu, H., and Li, Q. (2018). A spatial econometric modeling of online social interactions using microblogs. Comput. Environ. Urban Syst. *70*, 53–58. https://doi.org/10.1016/j.compenvurbsys.2018.02.001.
31. Fang, C., Yu, X., Zhang, X., Fang, J., and Liu, H. (2020). Big data analysis on the spatial networks of urban agglomeration. Cities *102*, 102735. https://doi.org/10.1016/j.cities.2020.102735.
32. Barthélemy, M. (2011). Spatial networks. Phys. Rep. *499*, 1–101. https://doi.org/10.1016/j.physrep.2010.11.002.
33. Hirtle, S.C., and Jonides, J. (1985). Evidence of hierarchies in cognitive maps. Mem. Cognit. *13*, 208–217. https://doi.org/10.3758/BF03197683.
34. Stevens, A., and Coupe, P. (1978). Distortions in judged spatial relations. Cognit. Psychol. *10*, 422–437. https://doi.org/10.1016/0010-0285(78)90006-3.
35. Wilton, R.N. (1979). Knowledge of spatial relations: The specification of the information used in making inferences. Q. J. Exp. Psychol. *31*, 133–146. https://doi.org/10.1080/14640747908400713.
36. Goodfellow, I., Bengio, Y., and Courville, A. (2016). Deep Learning (MIT Press).
37. Williams, H.C.W.L. (1977). On the formation of travel demand models and economic evaluation measures of user benefit. Environ. Plann. *9*, 285–344. https://doi.org/10.1068/a090285.
38. Goodfellow, I., et al. (2014). Generative adversarial nets Proc. 28th Conference on Neural Information Processing Systems*2*, 2672–2680. https://doi.org/10.5555/2969033.2969125.
39. Lute, R.D. (1959). Individual Choice Behavior (John Wiley Press).
40. Train, K.E. (2009). Discrete Choice Methods with Simulation (Cambridge University Press).
41. Sifringer, B., Lurkin, V., and Alahi, A. (2020). Enhancing discrete choice models with representation learning. Transp. Res. Part B Methodol. *140*, 236–261. https://doi.org/10.1016/j.trb.2020.08.006.
42. Bishop, C.M. (1995). Neural Networks for Pattern Recognition (Oxford University Press).
43. Liu, Y., Wei, W., Sun, A., and Miao, C. (2014). Exploiting geographical neighborhood characteristics for location recommendation. In Proc. 23rd ACM International Conference on Information and Knowledge Management, pp. 739–748. https://doi.org/10.1145/2661829.2662002.
44. Cho, E., Myers, S.A., and Leskovec, J. (2011). Friendship and mobility: User movement in location-based social networks. In Proc. 17th ACM SIGKDD International Conference on Knowledge Discovery and Data Mining, pp. 1082–1090. https://doi.org/10.1145/2020408.2020579.
45. Yang, L., Zhao, P., Liu, B., Gao, Y., Zhou, H., Li, Q., Jiang, Y., and Yang, Z. (2022). Network patterns of zhongyuan urban agglomeration in China based on baidu migration data. Ann. Transl. Med. *10*, 62. https://doi.org/10.3390/ijgi11010062.
46. Kii, M. (2021). Projecting future populations of urban agglomerations around the world and through the 21st century. npj Urban Sustain. *1*, 10. https://doi.org/10.1038/s42949-020-00007-5.
47. Barbosa, H., de Lima-Neto, F.B., Evsukoff, A., and Menezes, R. (2015). The effect of recency to human mobility. EPJ Data Sci. *4*, 21. https://doi.org/10.1140/epjds/s13688-015-0059-8.
48. Alessandretti, L., Sapiezynski, P., Sekara, V., Lehmann, S., and Baronchelli, A. (2018). Evidence for a conserved quantity in human mobility. Nat. Human Behav. *2*, 485–491. https://doi.org/10.1038/s41562-018-0364-x.
49. Meng, L., Somenahalli, S., and Berry, S. (2020). Policy implementation of multi-modal (shared) mobility: Review of a supply-demand value proposition canvas. Transport Rev. *40*, 670–684. https://doi.org/10.1080/01441647.2020.1758237.
50. Štrumbelj, E., and Kononenko, I. (2014). Explaining prediction models and individual predictions with feature contributions. Knowl. Inf. Syst. *41*, 647–665. https://doi.org/10.1007/s10115-013-0679-x.
51. Lundberg, S.M., and Lee, S.I. (2017). A unified approach to interpreting model predictions. In Proc. 30th Conference on Neural Information Processing Systems, pp. 4765–4774. https://doi.org/10.5555/3295222.3295230.
52. Chen, Y. (2023). Multi-scale-unified-model. https://doi.org/10.5281/zenodo.8175023.
53. American Regional Planning Association (2007). Northeast Megaregion 2050. https://rpa.org/work/reports?series=america-2050.
54. European Union (2001). ESPON Metropolitan Areas in the United Kingdom. https://en.wikipedia.org/wiki/ESPON_metropolitan_areas_in_the_United_Kingdom.
55. Pappalardo, L., Simini, F., Barlacchi, G., and Pellungrini, R. (2019). Scikit-mobility: A Python library for the analysis, generation and risk assessment of mobility data. Preprint at arXiv. https://doi.org/10.48550/arXiv.1907.07062.
56. United States Census Bureau (2010). 2010 Census. https://www.census.gov/quickfacts/fact/table/US/POP010210.
57. Office for National Statistics (2011). 2011 Census. https://www.ons.gov.uk/.
58. Kingma, D.P., and Ba, J. (2015). Adam: A method for stochastic optimization. Preprint atProc. 3rd International Conference on Learning Representations arXiv. https://doi.org/10.48550/arXiv.1412.6980.

**Patterns, Volume *4***

## Supplemental information

# A multi-scale unified model of human mobility in urban agglomerations

**Yong Chen, Haoge Xu, Xiqun (Michael) Chen, and Ziyou Gao**

# Supplemental Information

## Supplemental Items

**Supplemental Notes**

**Note S1. Nested logit (NL) model**

The NL model [1] represents individual travel choice behavior in a nested form (see Figure S2), and the probability $P^i(c)$ of selecting alternative $c$ at the first layer can be represented as:

$$P^i(c) = \frac{e^{\theta_1 V_c^i}}{\sum_{j\in\mathbb{C}} e^{\theta_1 V_j^i}} \tag{1}$$

$$V_c^i = \sum_{d=1}^{D} \beta_d^1 x_{dc}^i \tag{2}$$

where $V_c^i$ represents the utility function of individual $i$ selecting alternative $c$, which is calculated from $D$ attributes $x_{dc}^i$ (e.g., time, and cost). $\mathbb{C}$ is the set of alternatives at the first layer. $\theta_1$ is the scale parameter of the first layer. $\beta_d^1$ is the preference parameter of each attribute at the first layer. Based on the current selection, the probability $P^i(cq)$ of selecting alternative $q$ in nest $c$ can be represented as follows:

$$P^i(cq) = P^i(q|c)P^i(c) \tag{3}$$

$$P^i(q|c) = \frac{e^{\theta_2 V_{cq}^i}}{\sum_{j\in\mathbb{Q}} e^{\theta_2 V_{cj}^i}} \tag{4}$$

$$V_{cq}^i = \sum_{d=1}^{D} \beta_d^2 x_{dcq}^i \tag{5}$$

where the conditional selection probability $P^i(q|c)$ is calculated by the utility function of alternatives. $\mathbb{Q}$ is the set of alternatives in nest $c$. $\theta_2$ is the scale parameter of the second layer. $\beta_d^2$ is the preference parameter of attribute $x_{dcq}^i$ in nest $c$. To strengthen the relationship between the upper and lower layers in the NL model, the utility expectation values of all the alternatives of the lower layer are used as an attribute of its upper layer model to realize the feedback of the lower layer to the upper layer, and Eq. (2) is modified as follows:

$$V_c^i = \sum_{d=1}^{D} \beta_d^1 x_{dc}^i + \frac{1}{\theta_c} \cdot \ln(\sum\nolimits_{j\in\mathbb{Q}} \theta_c\, e^{V_{cj}^i}) \tag{6}$$

where $\theta_c$ is the corresponding model parameter.

**Note S2. Convolutional neural network (CNN)**

Deep CNN without bias parameter is used as a classifier to automatically extract human travel patterns, and correctly output travel choice probabilities at different spatial scales based on individual memory and population attractiveness feature inputs. At each scale, deep CNN is constructed by four one-dimensional convolution layers, and the convolution calculation of each layer is shown below:

$$\mathbf{X}_d^{l+1} = relu(\sum_{d=1}^{D} \mathbf{X}_d^l \, \boldsymbol{\beta}_d^l) \tag{7}$$

where $\mathbf{X}_d^l$ denotes the $d$-th dimension feature inputs of the $l$-th convolutional layer, and the first layer includes original individual memory and population attractiveness features. $D$ denotes the dimension of the feature vector. $\boldsymbol{\beta}$ denotes the parameters of the convolution filter. $relu(\cdot)$ denotes the rectified linear unit function [2]. CNN at different scales have similar model frameworks, and none use bias parameters. At the county scale, the number of filters in each layer of CNN is 16, 16, 16, and 1, respectively. At the community scale, the number of filters in each layer of CNN is 16, 32, 32, and 1, respectively. In addition, the last layer of CNN multiplies the model output of CNN at the county scale to implement conditional probability constraints. At the same time, the classification error calculated in the community scale back-propagates to the upper layer (i.e., CNN at the county scale) to update network parameters.

**Note S3. Graph generative adversarial network**

We model the prediction of travel probability between different locations as a process of missing data imputation, and propose graph generative adversarial networks (GGAN) to impute data. A generative adversarial network (GAN) is a deep learning framework consisting of two neural networks (i.e., generator, and discriminator) pitted against each other. It has been widely used to solve tasks such as image restoration and traffic prediction [3]. As shown in Figure S1, GGAN consists of generator $G$ and discriminator $D$. Considering that the transition between locations can be expressed as a travel network, it has a graph structure with non-Euclidean characteristics. Therefore, we employ a graph convolutional network as generator $G$. Assumed that the trip distribution matrix between $C$ locations is represented as $\mathbf{X} \in \mathbb{R}^{C \times C}$, and its component $x_{ij}$ represents the travel probability between two locations, calculated by the travel volume between locations. Note that the travel volume between some locations is unknown; thus, the travel probability cannot be calculated, which we treat as missing data. The missing state of the travel probability between locations is represented by a matrix $\mathbf{Ms} \in \{0,1\}^{C \times C}$. We use generator $G$ to automatically learn the mapping relationship between travel probability and distance, population difference between two locations, and then impute the travel probability between those locations with unknown travel volume. The imputation process is calculated as follows:

$$\mathbf{h}_d = LeakyRelu(\mathbf{D} \odot \mathbf{X} \cdot \mathbf{W}_d) \tag{8}$$

$$\mathbf{h}_p = LeakyRelu(\mathbf{PD} \odot \mathbf{X} \cdot \mathbf{W}_p) \tag{9}$$

$$\tilde{\mathbf{X}} = LeakyRelu([\mathbf{h}_d, \mathbf{h}_p] \cdot \mathbf{W}_{dp}) \tag{10}$$

where $\mathbf{D}$ denotes the spatial distance matrix. $\mathbf{PD}$ denotes the population difference matrix. Referring to previous empirical studies [4], we use the distance between locations, and the population difference between locations as the feature matrix. The component in $\mathbf{PD}$ is calculated by $1 + \frac{\log(n_2) - \log(n_1)}{\log(n_1)}$, wherein $n$ denotes population size. $\mathbf{h}_d$ and $\mathbf{h}_p$ denote a distance map and a population map obtained after feature extraction by graph convolution, respectively. $[\cdot]$ denotes concatenation operation. $\mathbf{W}_{dp}$, $\mathbf{W}_d$, and $\mathbf{W}_p$ denote the corresponding parameter matrix. $\odot$ denotes the Hadamard product [5] between two matrices. $LeakyRelu(\cdot)$ denotes the leaky rectified linear unit activation function. $\tilde{\mathbf{X}}$

denotes the imputed trip distribution matrix obtained by the convolutional operation after concatenating the distance map and the feature map.

On the other hand, discriminator $D$ is used to discriminate which components are observable and which are missing in $\widetilde{\mathbf{X}}$. The inputs of the discriminator include matrix $\widetilde{\mathbf{X}}$ and reminder matrix $\mathbf{Mr}$. Inspired by the research of Yoon et al. [6], $\mathbf{Mr} = \mathbf{Ms} \odot \mathbf{B} + 0.5(1 - \mathbf{B})$ is used to improve the convergence of the discriminator, wherein $\mathbf{B} \in \{0,1\}^{C \times C}$ denotes a random matrix. In $\mathbf{Mr}$, the element values include 0, 0.5, and 1. 0 and 1 indicate to remind the discriminator whether the corresponding element is missing. 0.5 denotes a neutral state, which means that no reminder is given to the discriminator. We use a three-layer fully connected network to represent the discriminator, and the discrimination process is calculated as follows:

$$\mathbf{h}_{dis}^{l+1} = LeakyRelu([\widetilde{\mathbf{X}}, \mathbf{Mr}] \cdot \mathbf{W}_{dis}^{l}) \tag{11}$$

$$\mathbf{Ms} = \sigma(\mathbf{h}_{dis}^{l+1} \mathbf{W}_{dis}^{l+1}) \tag{12}$$

where $\mathbf{h}_{dis}^{l+1}$ denotes the output of the $l$-th layer of the discrimination network, which serves as a feature input of the next layer. $\mathbf{Ms}$ is a discriminant matrix, whose components take values from zero to one, indicating the missing possibility of the corresponding travel volume. $\mathbf{W}_{dis}^{l}$ and $\mathbf{W}_{dis}^{l+1}$ represent the corresponding parameter matrix. $\sigma(\cdot)$ denotes the sigmoid function.

The generator and discriminator are trained adversarially through a minimax game [7], and their loss functions are defined as follows:

$$\mathcal{L}_D = -\mathbb{E}[(1 - \mathbf{B}) \odot \mathbf{Ms} \odot \log(\mathbf{Ms}) + (1 - \mathbf{B}) \odot (1 - \mathbf{Ms}) \odot \log(1 - \mathbf{Ms})] \tag{13}$$

$$\mathcal{L}_G = \mathcal{L}_{G1} + \beta \cdot \mathcal{L}_{G2} = -\mathbb{E}[(1 - \mathbf{B}) \odot (1 - \mathbf{Ms}) \odot \log(\mathbf{Ms})] + \beta \cdot RMSE(\mathbf{X} \odot \mathbf{Ms}, \widetilde{\mathbf{X}} \odot \mathbf{Ms}) \tag{14}$$

where $\mathcal{L}_D$ denotes the probability of correctly predicting the missing matrix $\mathbf{Ms}$, and the discriminator is trained by minimizing $\mathcal{L}_D$. The generator is trained by minimizing $\mathcal{L}_G$, which consists of the discriminant error and the reconstruction error. $\beta$ denotes the weight coefficient. The generator aims to make the observable components of $\widetilde{\mathbf{X}}$ is consistent with the actual data, and the missing components discriminator is challenging to distinguish the true from the false.

**Supplemental Tables**

**Table S1.** Prediction performance comparison of different models at county and community scales in Boswash urban agglomeration

| Name | Abb. | GM | | | RM | | | PWO | | | GGAN | | |
|---|---|---|---|---|---|---|---|---|---|---|---|---|---|
| | | CPC | RMSE | MAE | CPC | RMSE | MAE | CPC | RMSE | MAE | CPC | RMSE | MAE |
| ALL | ALL | 0.446 | **65.567** | 10.875 | 0.470 | 164.506 | 9.965 | 0.406 | 191.785 | 17.470 | **0.490** | 82.201 | **8.906** |
| Ave. | Ave. | 0.197 | 5.236 | 0.787 | 0.379 | 4.902 | 0.545 | 0.459 | 4.206 | 0.592 | **0.503** | **3.498** | **0.475** |
| New York County | NY | 0.584 | 214.197 | 38.216 | 0.509 | 237.877 | 32.197 | 0.666 | 166.731 | 32.351 | **0.743** | **164.708** | **25.150** |
| District of Columbia | DC | 0.448 | 53.163 | 6.927 | 0.531 | 50.169 | 4.173 | 0.656 | 41.852 | 3.702 | **0.806** | **18.889** | **2.394** |
| Suffolk County (Massachusetts) | SCM | 0.289 | 48.819 | 4.911 | 0.455 | 35.250 | 2.462 | 0.514 | 30.907 | 2.483 | **0.723** | **16.787** | **1.825** |
| Kings County | KC | 0.368 | 40.489 | 6.025 | 0.596 | 29.489 | **2.934** | 0.647 | 29.432 | 3.115 | **0.648** | **27.695** | 3.308 |
| Philadelphia County | PC | 0.273 | 23.110 | 1.882 | 0.317 | 20.768 | **1.140** | 0.420 | 19.065 | 1.256 | **0.481** | **16.014** | 1.468 |
| Baltimore County | BC | 0.271 | 1.785 | 0.255 | 0.486 | 1.295 | 0.144 | 0.612 | **1.175** | 0.160 | **0.668** | 1.289 | **0.132** |
| Middlesex County | MC | 0.351 | 14.393 | 1.946 | 0.527 | 9.330 | 1.043 | 0.405 | 12.761 | 1.867 | **0.660** | **6.671** | **0.998** |
| Queens County | QC | 0.325 | 8.322 | 1.315 | 0.452 | **7.465** | **0.758** | **0.545** | 7.742 | 0.904 | 0.431 | 7.813 | 0.991 |
| Nassau County | NC | 0.404 | 4.623 | 0.768 | 0.522 | **3.176** | **0.453** | **0.598** | 4.497 | 0.698 | 0.567 | 3.599 | 0.625 |
| Suffolk County (New York) | SCN | 0.308 | 4.186 | 0.897 | 0.541 | 3.180 | **0.442** | 0.604 | 4.385 | 0.805 | **0.607** | **3.134** | 0.536 |

Note: "Abb." stands for abbreviation. Bold font represents the optimal value of each measure of effectiveness.

**Table S2.** Prediction performance comparison of different models at county and community scales in Great Lakes urban agglomeration

| Name | Abb. | GM | | | RM | | | PWO | | | GGAN | | |
|---|---|---|---|---|---|---|---|---|---|---|---|---|---|
| | | CPC | RMSE | MAE | CPC | RMSE | MAE | CPC | RMSE | MAE | CPC | RMSE | MAE |
| ALL | ALL | 0.135 | 17.412 | 1.304 | 0.437 | 14.293 | **0.623** | 0.272 | 16.581 | 0.909 | **0.505** | **12.801** | 0.771 |
| Ave. | Ave. | 0.134 | 2.174 | 0.243 | 0.329 | 1.737 | 0.128 | 0.358 | 1.888 | 0.163 | **0.522** | **1.257** | **0.117** |
| Dane County | DC | 0.272 | 5.772 | 0.787 | 0.551 | 3.584 | 0.348 | 0.645 | 3.190 | 0.391 | **0.703** | **2.897** | **0.322** |
| Milwaukee County | MIC | 0.201 | 13.000 | 1.207 | 0.559 | 8.736 | 0.468 | 0.288 | 12.272 | 0.702 | **0.768** | **5.607** | **0.329** |
| Cook County | COC | 0.351 | 31.144 | 3.607 | 0.586 | 20.250 | 1.698 | 0.534 | 27.850 | 2.785 | **0.713** | **15.993** | **1.402** |
| Oakland County | OC | 0.395 | 8.261 | 1.395 | 0.512 | 7.179 | 0.823 | 0.609 | 8.140 | 1.166 | **0.639** | **4.863** | **0.822** |
| Marion County | MC | 0.547 | 1.935 | 0.429 | 0.545 | 1.924 | **0.323** | 0.452 | 5.002 | 0.773 | **0.662** | **1.478** | 0.327 |
| Franklin County | FC | 0.378 | 24.525 | 2.261 | 0.431 | 21.105 | 1.376 | 0.476 | 19.872 | 1.600 | **0.608** | **17.193** | **1.356** |
| Cuyahoga County | CC | 0.423 | 4.367 | 0.668 | 0.460 | 4.281 | 0.467 | **0.685** | **3.392** | **0.383** | 0.659 | 4.158 | 0.437 |
| Allegheny County | AC | 0.407 | 8.212 | 1.199 | 0.389 | 7.787 | 0.796 | 0.613 | **5.207** | 0.859 | **0.629** | 5.617 | **0.746** |
| Erie County | EC | 0.287 | 8.173 | 1.108 | 0.397 | 7.758 | 0.622 | 0.533 | 6.297 | 0.676 | **0.622** | **4.571** | **0.543** |
| Monroe County | MOC | 0.392 | 12.362 | 1.807 | 0.563 | 10.200 | 0.924 | 0.619 | 8.850 | 1.061 | **0.707** | **7.893** | **0.867** |

**Table S3.** Prediction performance comparison of different models at county and community scales in England urban agglomeration

| Names | Abb. | GM | | | RM | | | PWO | | | GGAN | | |
|---|---|---|---|---|---|---|---|---|---|---|---|---|---|
| | | CPC | RMSE | MAE | CPC | RMSE | MAE | CPC | RMSE | MAE | CPC | RMSE | MAE |
| ALL | ALL | 0.485 | **15.592** | 4.861 | 0.387 | 33.848 | 4.772 | 0.487 | 25.451 | 5.072 | **0.571** | 17.537 | **4.420** |
| Ave. | Ave. | 0.071 | 0.403 | 0.041 | 0.265 | 0.322 | **0.026** | 0.373 | 0.364 | 0.039 | **0.381** | **0.279** | 0.028 |
| Brighton and Hove County | BH | 0.233 | 2.275 | 0.286 | 0.288 | 2.182 | 0.173 | 0.303 | 2.340 | 0.238 | **0.644** | **1.728** | **0.132** |
| Camden County | CD | 0.157 | 0.620 | 0.099 | **0.474** | **0.423** | **0.062** | 0.459 | 0.899 | 0.133 | 0.426 | 0.522 | 0.078 |
| Hertfordshire County | HS | 0.156 | 1.228 | 0.119 | 0.364 | 1.131 | 0.065 | 0.419 | 1.292 | 0.112 | **0.617** | **0.601** | **0.057** |
| Lancashire County | LS | 0.077 | 0.756 | 0.095 | 0.416 | 0.522 | **0.052** | 0.496 | 0.532 | 0.078 | **0.557** | **0.485** | 0.058 |
| Manchester County | MC | 0.233 | 2.462 | 0.387 | 0.516 | 1.740 | 0.195 | 0.501 | 2.141 | 0.289 | **0.596** | **1.464** | **0.190** |
| South Yorkshire County | SY | 0.152 | 0.748 | 0.099 | 0.333 | 0.675 | 0.058 | 0.421 | 0.707 | 0.079 | **0.432** | **0.574** | **0.057** |
| Tyne and Wear County | TW | 0.215 | 0.714 | 0.081 | 0.219 | 0.677 | 0.063 | 0.276 | 0.901 | 0.113 | **0.515** | **0.466** | **0.057** |
| West Midlands County | WMS | 0.154 | 1.320 | 0.199 | 0.316 | 1.119 | 0.128 | 0.455 | 0.976 | 0.172 | **0.518** | **0.953** | **0.117** |
| West Yorkshire County | WY | 0.099 | 1.904 | 0.160 | 0.333 | 1.369 | 0.099 | 0.478 | 1.132 | 0.128 | **0.516** | **0.758** | **0.095** |
| Westminster County | WM | 0.341 | 1.456 | 0.313 | 0.444 | 1.050 | **0.217** | **0.535** | 1.923 | 0.367 | 0.519 | **1.009** | 0.252 |

**Table S4.** CPC values of different models at different active regions

| Active regions | Boswash agglomeration | | | | Great Lakes agglomeration | | | | England agglomeration | | | |
|---|---|---|---|---|---|---|---|---|---|---|---|---|
| | GM | RM | PWO | GGAN | GM | RM | PWO | GGAN | GM | RM | PWO | GGAN |
| 1 | 0.373 | 0.501 | 0.564 | **0.634** | 0.364 | 0.497 | 0.545 | **0.665** | 0.182 | 0.370 | 0.434 | **0.534** |
| 2 | 0.287 | 0.419 | **0.533** | 0.526 | 0.269 | 0.484 | 0.511 | **0.637** | 0.140 | 0.393 | 0.460 | **0.484** |
| 3 | 0.259 | 0.431 | **0.512** | 0.504 | 0.169 | 0.397 | 0.314 | **0.673** | 0.120 | 0.307 | **0.402** | 0.363 |
| 4 | 0.205 | 0.435 | 0.531 | **0.548** | 0.154 | 0.390 | 0.404 | **0.515** | 0.083 | 0.349 | 0.407 | **0.430** |
| 5 | 0.190 | 0.355 | 0.477 | **0.486** | 0.090 | 0.298 | 0.404 | **0.494** | 0.051 | 0.286 | 0.409 | **0.421** |
| 6 | 0.183 | 0.327 | 0.393 | **0.453** | 0.100 | 0.256 | 0.176 | **0.464** | 0.071 | 0.312 | 0.411 | **0.494** |
| 7 | 0.181 | 0.394 | 0.471 | **0.479** | 0.076 | 0.358 | 0.333 | **0.540** | 0.014 | 0.166 | **0.358** | 0.304 |
| 8 | 0.128 | 0.382 | 0.410 | **0.519** | 0.069 | 0.224 | 0.266 | **0.417** | 0.037 | 0.258 | **0.355** | 0.328 |
| 9 | 0.103 | 0.295 | 0.360 | **0.475** | 0.000 | 0.163 | 0.285 | **0.423** | 0.000 | 0.139 | **0.265** | 0.220 |
| 10 | 0.041 | 0.237 | 0.321 | **0.400** | 0.000 | 0.168 | **0.335** | 0.308 | 0.000 | 0.021 | 0.196 | **0.197** |

**Table S5.** Performance comparison of multi-scale human mobility prediction in different agglomerations

| Urban Agglomeration | Measure of effectiveness | EPR | D-EPR | R-EPR | M-EPR | UMIP | MSUM |
|---|---|---|---|---|---|---|---|
| Boswash | CPC | 0.170 | 0.267 | 0.263 | 0.378 | 0.310 | **0.672** |
| | RMSE | 29.066 | 28.665 | 25.043 | 23.839 | 24.956 | **13.220** |
| | MAE | 4.285 | 4.150 | 4.141 | 3.706 | 4.903 | **2.791** |
| Great Lakes | CPC | 0.064 | 0.192 | 0.137 | 0.222 | 0.444 | **0.542** |
| | RMSE | 19.396 | 18.877 | 18.629 | 17.447 | 22.385 | **14.387** |
| | MAE | 3.918 | 3.679 | 3.782 | 3.594 | 3.601 | **3.324** |
| England | CPC | 0.085 | 0.128 | 0.125 | 0.175 | 0.221 | **0.319** |
| | RMSE | 2.991 | 2.956 | 2.863 | 2.516 | 3.647 | **2.072** |
| | MAE | 1.376 | 1.348 | 1.369 | 1.321 | 1.497 | **1.268** |

**Table S6.** Performance comparison of human mobility prediction at the county scale in different agglomerations

| Urban Agglomeration | Measure of effectiveness | EPR | D-EPR | R-EPR | M-EPR | UMIP | MSUM |
|---|---|---|---|---|---|---|---|
| Boswash | CPC | 0.383 | 0.432 | 0.535 | 0.592 | 0.415 | **0.877** |
| | RMSE | 533.318 | 510.448 | 392.632 | 337.654 | 506.146 | **51.634** |
| | MAE | 37.670 | 38.070 | 31.640 | 28.423 | 42.217 | **9.322** |
| Great Lakes | CPC | 0.228 | 0.412 | 0.360 | 0.397 | 0.669 | **0.816** |
| | RMSE | 123.701 | 109.841 | 111.531 | 106.540 | 55.371 | **36.702** |
| | MAE | 18.708 | 17.417 | 17.190 | 16.393 | 12.032 | **7.235** |
| England | CPC | 0.556 | 0.543 | 0.564 | 0.623 | 0.499 | **0.736** |
| | RMSE | 13.462 | 14.029 | 11.868 | 10.481 | 16.078 | **4.448** |
| | MAE | 3.238 | 3.541 | 3.412 | 2.906 | 4.303 | **2.188** |

## Supplemental Figures

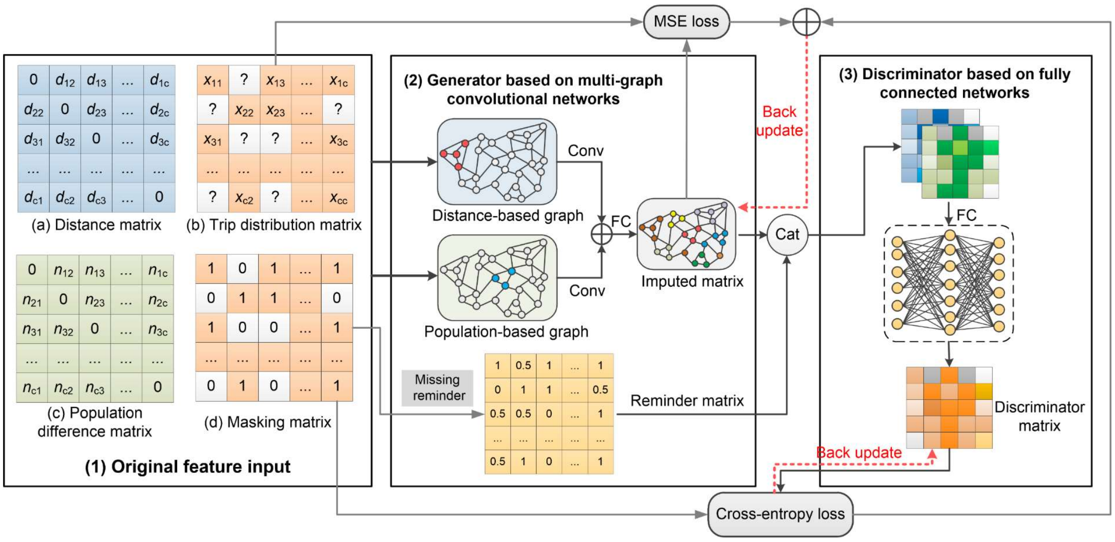


**Figure S1. Architecture of graph generative adversarial networks model.** The model consists of three modules: original feature input, generator, and discriminator. The feature inputs include a trip distribution matrix between locations, a masking matrix representing the missing data state, and distance and population difference matrices used to construct the virtual graph. The implementation of the generator includes two steps. First, based on the distance matrix and population difference matrix, the graph convolution operations (i.e., "Conv") based on the distance and population graphs are performed, respectively. Second, the feature matrices are concatenated after feature extraction, input into a fully connected network (i.e., "FC") for mixed feature extraction, and an imputed trip distribution matrix is output. The discriminator is represented by a fully connected network whose feature inputs include an imputed missing matrix and a missing reminder matrix, where the missing reminder matrix is used to accelerate the discriminator's convergence. "Cat" denotes the concatenate operation. The loss function of the generator consists of the discriminant error (i.e., cross-entropy loss) and reconstruction error (i.e., mean squared error loss). The loss function of the discriminator is the cross-entropy loss.

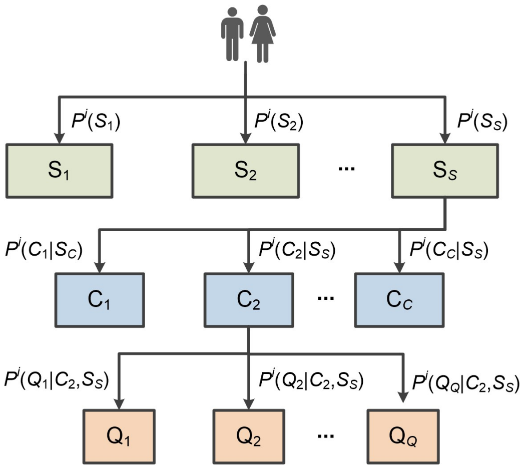


**Figure S2. Schematic representation of the nested logit model.** Individuals hierarchically select alternatives in a nested structure (see Note S2), and the selection process is constrained by conditional probability. At the first layer, the individual selects alternative $S_S$ with probability $P^i(S_S)$. Then, in nest $S_S$, alternative $C_C$ is selected with probability $P^i(C_C|S_S)$. Further, in the selected nest $C_C$, alternative $Q_Q$ is selected with probability $P^i(Q_Q|C_C,S_S)$.

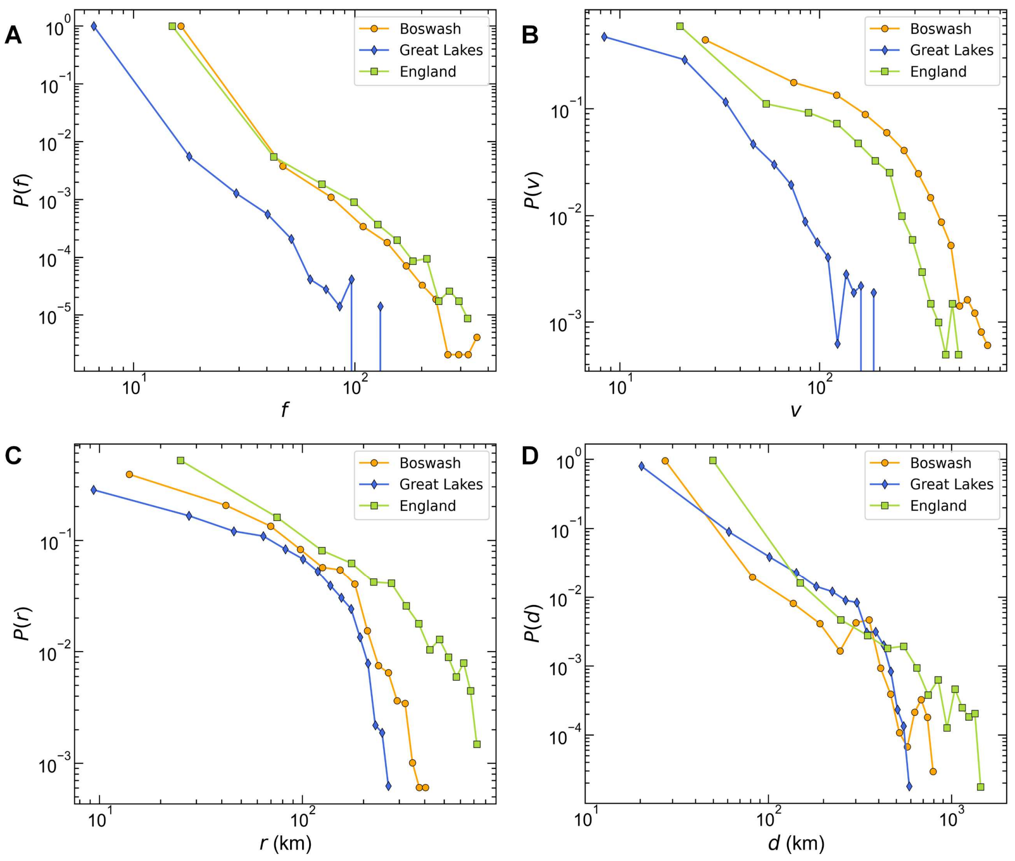


**Figure S3. Empirical statistics of human mobility patterns in three urban agglomerations.** (**A**) Frequency distribution of individuals visiting a location. (**B**) Probability distribution of $v$ different locations visited in total. (**C**) Radius of gyration distribution. (**D**) Travel distance distribution at multiple spatial scales. All distributions are calculated from actual individual travel traces, and individuals move at multiple spatial scales within urban agglomerations, including intra- and inter-county.

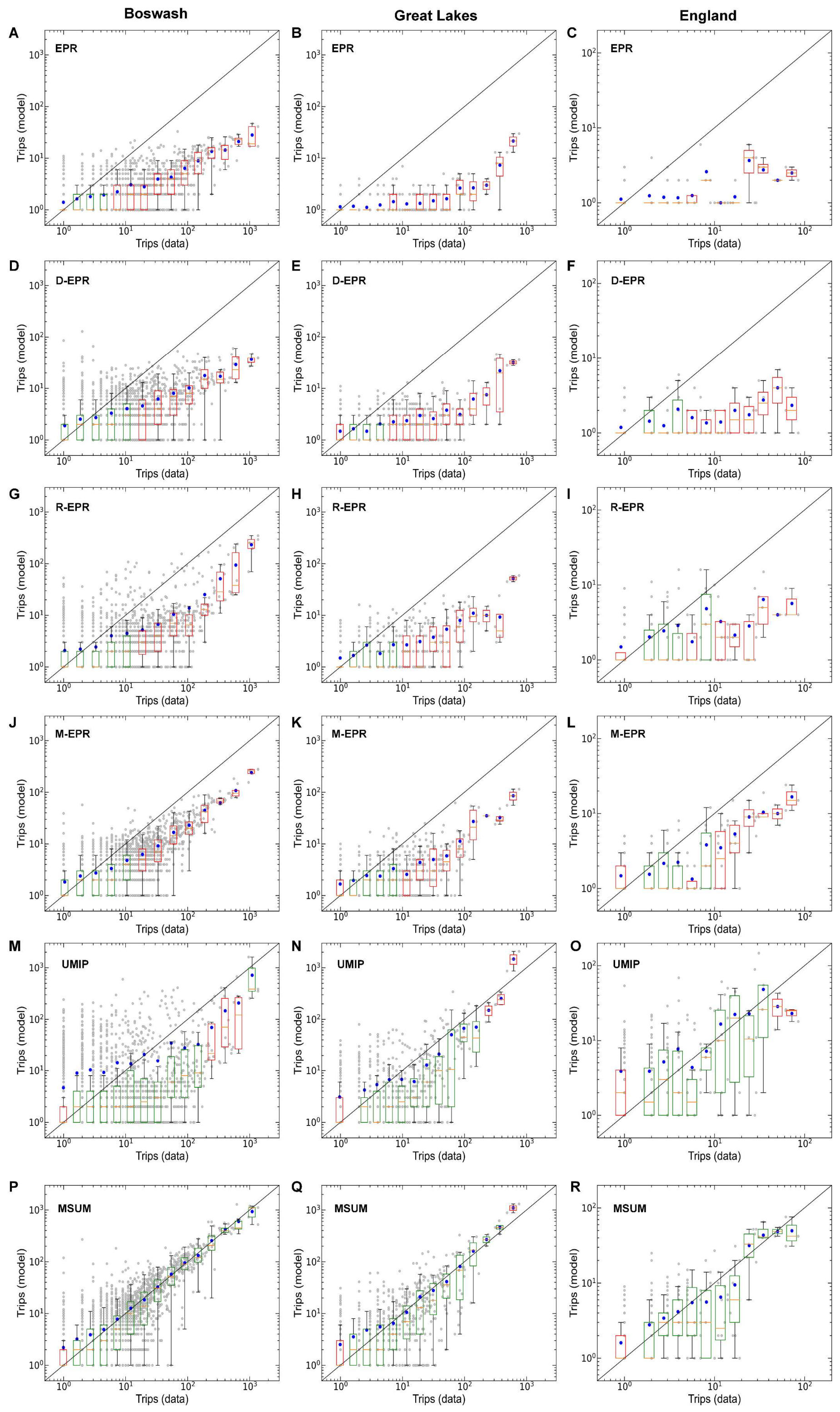


**Figure S4. Paired comparisons of predicted and real trips.** Gray points indicate observed and predicted location pairs. Blue points indicate the average number of predicted trips in different bins. The boxplot indicates the distribution of the number of predicted trips in different bins of the number of observed trips. A box is green if the black line $y = x$ is between the 5th and 95th percentiles of the box, and red otherwise. To characterize the model's prediction performance, all distributions are calculated by aggregating simulated and actual individual traces.

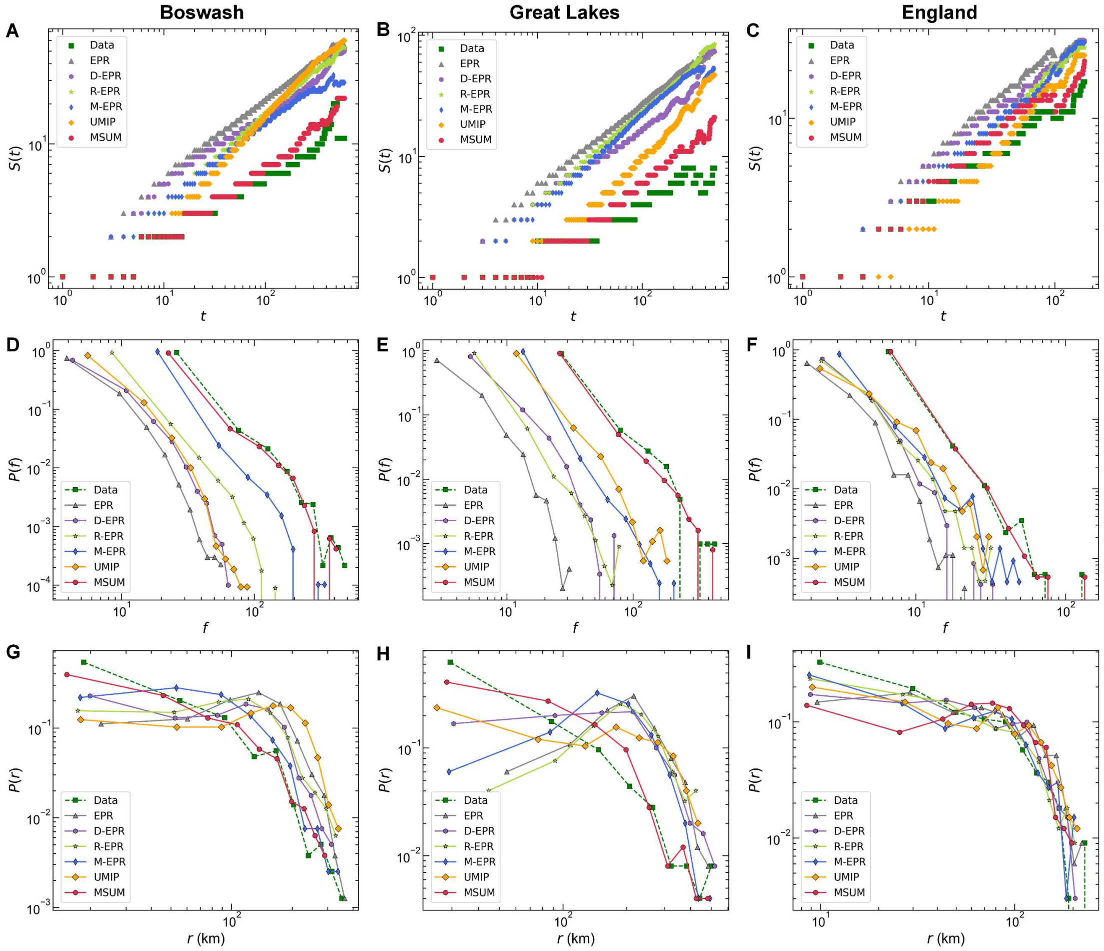


**Figure S5. Single-scale human mobility prediction in urban agglomeration at the individual level.** (**A–C**) Distribution of the total number of counties visited in $t$ trips. (**D–F**) Frequency distribution of individuals visiting a county. (**G–I**) Radius of gyration distribution. All distributions are calculated from the simulated and the actual individual traces. The calculated distributions focus on the individuals' travel characteristics between counties (i.e., single spatial scale).

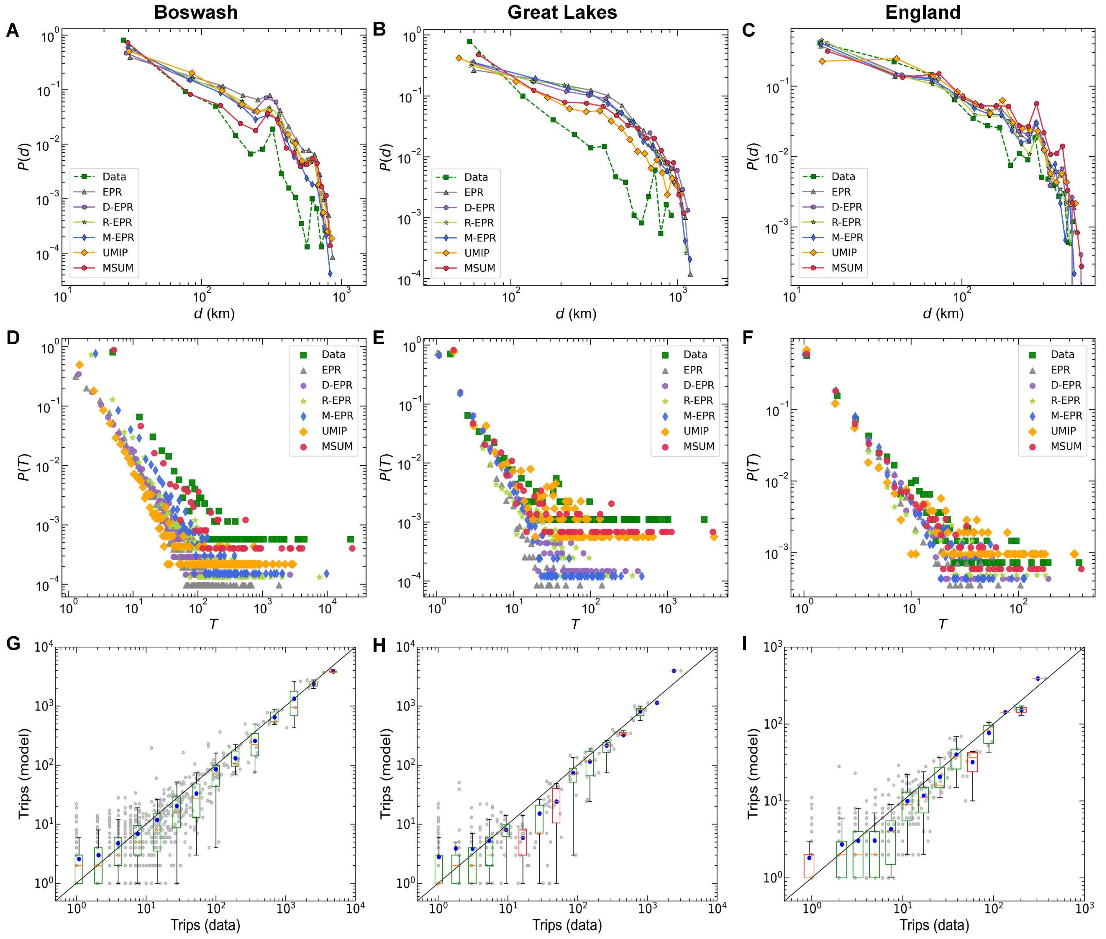


**Figure S6. Single-scale human mobility prediction in urban agglomeration at the population level.** (**A–C**) Predicted and real distributions of travel distance. (**D–F**) Predicted and real distributions of the number of trips between two counties. (**G–I**) Paired comparisons of predicted and real trips. Gray points indicate observed and predicted county pairs. Blue points indicate the average number of predicted trips in different bins. The boxplot indicates the distribution of the number of predicted trips in different bins of the number of observed trips. A box is green if the black line $y = x$ is between the 5th and 95th percentiles of the box, and red otherwise. To characterize the model's single-scale human mobility (i.e., inter-county travel) prediction performance at the population level, all distributions are calculated by aggregating simulated and actual individual traces, respectively.

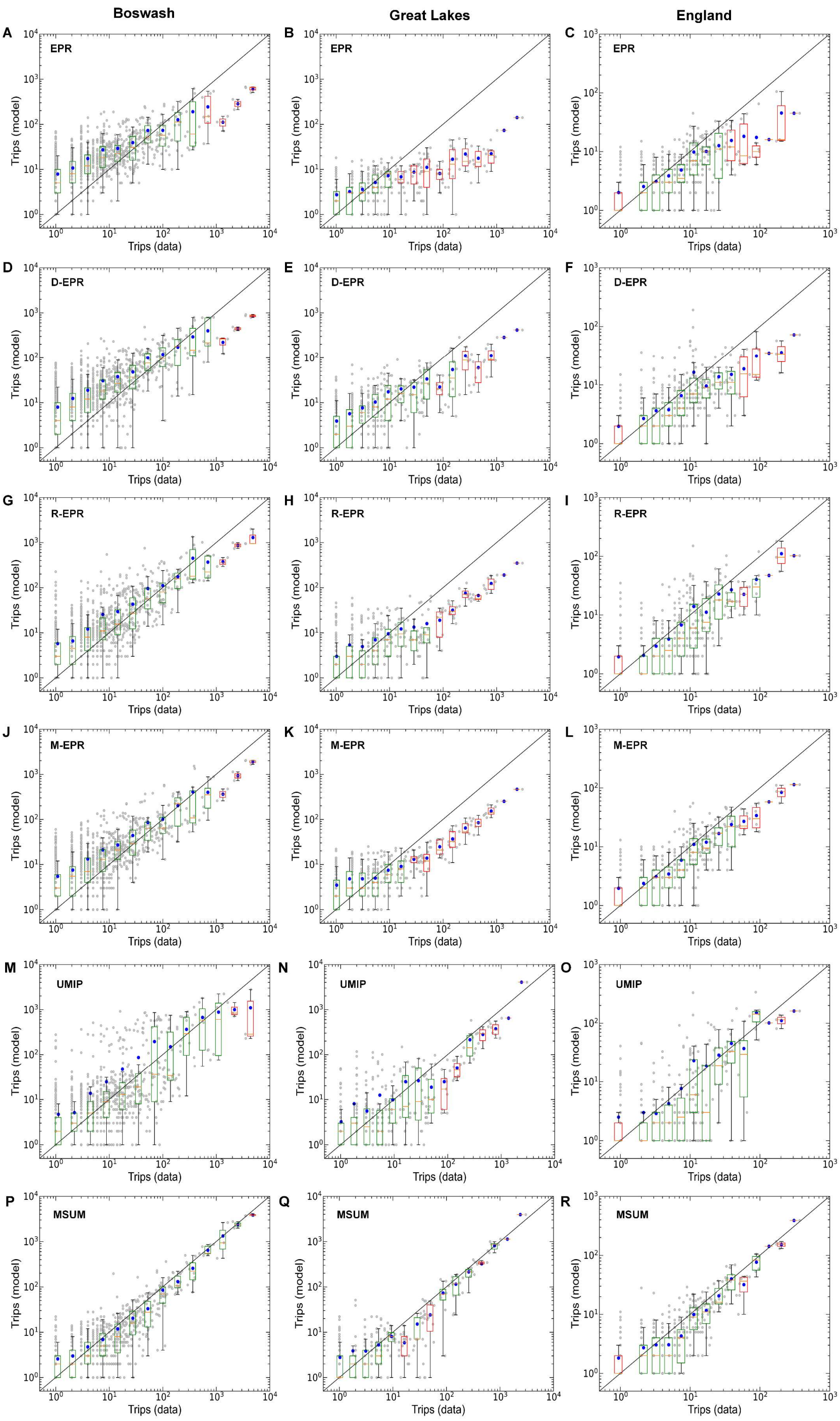


**Figure S7. Paired comparisons of predicted and real trips at the county scale.** Gray points indicate observed and predicted county pairs. Blue points indicate the average number of predicted trips in different bins. The boxplot indicates the distribution of the number of predicted trips in different bins of the number of observed trips. A box is green if the black line $y = x$ is between the 5th and 95th percentiles of the box, and red otherwise. To characterize the model's human mobility prediction performance at a single spatial scale, all distributions are calculated by aggregating simulated and actual individual traces, respectively.

## Supplemental Experimental Procedures

### Baseline models

To verify the accuracy and robustness of graph generative adversarial network (GGAN), three widely used models are selected as the baseline for performance comparison, including the gravity model (GM) [8], radiation model (RM) [9], and population-weighted opportunities model (PWO) [10].

GM model assumes that the travel volume between two locations is proportional to their population and decays as the power of the distance between them. We use the common origin-constrained form of GM, which is defined as follows:

$$T_{ij}^{GM} = T_{i,:} \cdot \frac{Pop_j^{\gamma_1} d_{ij}^{-\gamma_2}}{\sum_k Pop_k^{\gamma_1} d_{ik}^{-\gamma_2}} \tag{15}$$

where $T_{i,:}$ denotes the total travel flows from location $i$. $Pop_j$ denotes the population of location $j$. $d_{ij}$ denotes the distance between location $i$ and location $j$. $\gamma_1$ and $\gamma_2$ are parameters to be estimated.

RM model is inspired by the radiation and absorption of particles to make trip distribution predictions based on the population between locations without parameter estimation, and is defined as follows:

$$T_{ij}^{RM} = T_{i,:} \cdot \frac{Pop_i Pop_j}{(Pop_i + s_{ij})(Pop_i + s_{ij} + Pop_j)} \tag{16}$$

where $s_{ij}$ denotes the number of intervention opportunities (i.e., the total population) between location $i$ and location $j$.

PWO model assumes that the attractiveness of the destination declines with distance, and is inversely proportional to the population $S_{ji}$ in the circle centered at the destination with radius $d_{ij}$, minus a finite-size correction term:

$$T_{ij}^{PWO} = T_{i,:} \cdot \frac{Pop_j(1/S_{ji} - 1/P_{total})}{\sum_{k \neq i} Pop_k(1/S_{ki} - 1/P_{total})} \tag{17}$$

$$S_{ji} = Pop_i + s_{ji} + Pop_j \tag{18}$$

where $P_{total}$ is the total population of all locations.

Moreover, to verify the prediction performance of the multi-scale unified model (MSUM) at multiple spatial scales in urban agglomerations, the exploration and preferential return [11] (EPR) model, three EPR variant models, i.e., gravity EPR [12] (D-EPR), recency EPR [13] (R-EPR), and memory EPR [14] (M-EPR), and universal model of individual and population (UMIP) [15] are used for performance comparison.

In EPR model, for each new movement, an individual explores a new location with probability $\rho S^{-\gamma}$, or chooses to return to a previously visited location with probability $1 - \rho S^{-\gamma}$, where $S$ represents the total number of locations that the individual has visited. $\rho$ and $\gamma$ are the model parameters, and 0.6 and 0.21 are adopted in this paper, respectively, which is based on the empirical observations in previous study [4,11]. In the return phase, the probability of an individual visiting a location is proportional to the frequency of his/her previous visits. In the exploration phase, the individual chooses a location to travel according to the distance distribution among the locations.

D-EPR model optimizes the exploration phase of the EPR model. When exploring a new location, the individual chooses a new location to travel with selection probability $p_{ij} = \frac{1}{SP}\frac{Pop_i Pop_j}{d_{ij}^2}$ based on the gravity model, where $SP = \sum_{i,j\neq i} p_{ij}$ is a normalization factor.

R-EPR model optimizes the return phase of the EPR model by considering recency influence. R-EPR model represents the return probability of an individual through ranking, that is, people usually consider recently-visited locations to travel. In the return phase, the individual chooses to rank candidate locations based on the *frequency* with probability $\alpha$ or the *recency* with probability $1-\alpha$. In the first case, the individual selects a return destination with probability $Rank_f(l_i)^{-1-\gamma}$ based on the historical visit frequency, where $Rank_f(l_i)$ represents the frequency-based ranking of location $l_i$. In the latter case, the individual will choose the $i$-th last visited location to return to with probability $Rank_r(l_i)^{-\eta}$ selecting from a Zipf distribution, where $Rank_r(l_i)$ represents the recency-based ranking of location $l_i$. Parameters $\alpha$, $\gamma$, and $\eta$ are set to 0.1, 0.21, and 1.6, respectively, based on the empirical observations in the previous study [13].

M-EPR model optimizes the return phase of the EPR model by adding a memory limit to EPR. In the M-EPR model, only the historical memory of previous $M$ days affects the individual's travel choice behavior in the return phase. In our study, we set $M$ to be 92.17% of the total movement steps, which approximates the time ratio in the original study [14].

UMIP model combines individual memory effect and population-induced competition to achieve the unified individual and population mobility prediction.

$$p_{ij} \propto \frac{Pop_j}{S_{ji}}\left(1+\frac{\lambda}{r_j}\right) \tag{19}$$

where $p_{ij}$ denotes the transition probability from location $i$ to $j$. The individual memory effect (i.e., $1+\frac{\lambda}{r_j}$) is quantified by the rank of visits to different locations. $r_j$ denotes that location $j$ is the $r$-th newly visited location of the current individual. $\lambda$ denotes the model parameter used to characterize the strength of the memory effect, which is estimated from empirical data and takes values of 30 (Boswash), 30 (Great Lakes), and 60 (England), respectively. The population-induced competition (i.e., $\frac{Pop_j}{S_{ji}}$) is quantified by the population between the origin and the destination, and the specific calculation method is the same as that of PWO.

**Measures of Effectiveness**

Three widely used measures of effectiveness are used to quantify the model prediction performance, i.e., common part of commuters [4] (CPC), mean absolute error (MAE), and root mean squared error (RMSE), which are defined as follows:

$$\mathrm{CPC} = \sum_{i}^{N}\sum_{j\neq i}^{N}\frac{2\times\min(T_{ij}, T'_{ij})}{T_{ij}+T'_{ij}} \tag{20}$$

$$\mathrm{MAE} = \frac{1}{N(N-1)}\sum_{i}^{N}\sum_{j\neq i}^{N}|T_{ij}-T'_{ij}| \tag{21}$$

$$\mathrm{RMSE} = \sqrt{\frac{1}{N(N-1)}\sum_{i}^{N}\sum_{j\neq i}^{N}\left(T_{ij} - T'_{ij}\right)^2} \tag{22}$$

where $T_{ij}$ and $T'_{ij}$ denote observed and predicted travel flows, from location $i$ to location $j$, respectively. $N$ denotes the total number of locations.

**Performance comparison of single-scale mobility prediction between MSUM and baseline models**

By aggregating all individual simulation traces at the county level, we can compare the mobility prediction performance of MSUM with that of baseline models (i.e., EPR, D-EPR, R-EPR, and M-EPR) at a single spatial scale. Figures S5-S7 present the model comparison results at individual and population levels. At the same time, Table S6 quantifies the prediction performance of each model using CPC, RMSE, and MAE. At the individual level, MSUM can describe individual mobility patterns consistent with empirical distributions, whereas the baseline models have poor prediction accuracy in individual mobility patterns. At the population level, MSUM can accurately predict the inter-county travel flow, and has a stable prediction performance under different travel flow intensities. Meanwhile, as shown in Figure S7, the baseline models underestimate travel flow, especially in the range of $10^2$ through $10^3$. Therefore, the proposed MSUM can achieve superior performance in the single-scale human mobility prediction task.

## Supplemental References


1. Williams, H.C. (1977). On the formation of travel demand models and economic evaluation measures of user benefit. *Environ. Plan. A. 9*, 285–344. https://doi.org/10.1068/a090285
2. Nair, V., and Hinton, G.E. (2010). Rectified linear units improve restricted Boltzmann machines. In *Proc. 27th International Conference on Machine Learning*, 807–814. https://dl.acm.org/doi/10.5555/3104322.3104425
3. Creswell, A., White, T., Dumoulin, V., Arulkumaran, K., Sengupta, B., and Bharath, A.A. (2018). Generative adversarial networks: An overview. *IEEE Signal Process. Mag. 35*, 53–65. https://doi.org/10.1109/MSP.2017.2765202
4. Barbosa, H. et al. (2018). Human mobility: Models and applications. *Phys. Rep. 734*, 1–74. https://doi.org/10.1016/j.physrep.2018.01.001
5. Davis, C. (1962). The norm of the Schur product operation. *Numer. Math. 4*, 343–344. https://doi.org/10.1007/BF01386329
6. Yoon, J., Jordon, J., and Schaar, M. (2018). Gain: Missing data imputation using generative adversarial nets. In *Proc. International Conference on Machine Learning*, pp. 5689–5698. http://proceedings.mlr.press/v80/yoon18a.html
7. Goodfellow, I. et al. (2014). Generative adversarial nets. In *Proc. 28th Conference on Neural Information Processing Systems 2*, 2672–2680. https://dl.acm.org/doi/10.5555/2969033.2969125
8. Zipf, G.K. (1946). The $P_1$ $P_2$/D hypothesis: On the intercity movement of persons. *Am. Sociol. Rev. 11*, 677–686. https://doi.org/10.2307/2087063
9. Simini, F., González, M.C., Maritan, A., and Barabási, A.L. (2012). A universal model for mobility and migration patterns. *Nature 484*, 96–100. https://doi.org/10.1038/nature10856
10. Yan, X.Y., Zhao, C., Fan, Y., Di, Z.R., and Wang, W.X. (2014). Universal predictability of mobility patterns in cities. *J. R. Soc. Interface 11*, 20140834. https://doi.org/10.1098/rsif.2014.0834
11. Song, C., Koren, T., Wang, P., and Barabási, A.L. (2010). Modelling the scaling properties of human mobility. *Nat. Phys. 6*, 818–823. https://doi.org/10.1038/nphys1760
12. Pappalardo, L. et al. (2015). Returners and explorers dichotomy in human mobility. *Nat. Commun. 6*, 8166. https://doi.org/10.1038/ncomms9166
13. Barbosa, H., de Lima-Neto, F.B., Evsukoff, A., and Menezes, R. (2015). The effect of recency to human mobility. *EPJ Data Sci. 4*, 21. http://dx.doi.org/10.1140/epjds/s13688-015-0059-8
14. Alessandretti, L., Sapiezynski, P., Sekara, V., Lehmann, S., and Baronchelli, A. (2018). Evidence for a conserved quantity in human mobility. *Nat. Hum. Behav. 2*, 485–491. https://doi.org/10.1038/s41562-018-0364-x
15. Yan, X.Y., Wang, W.X., Gao, Z.Y., and Lai, Y.C. (2017). Universal model of individual and population mobility on diverse spatial scales. *Nat. Commun. 8*, 1639. https://doi.org/10.1038/s41467-017-01892-8
